# Predicting Absolute Rate Constants for Huisgen Reactions of Unsaturated Iminium Ions with Diazoalkanes

**DOI:** 10.1002/anie.202003029

**Published:** 2020-05-11

**Authors:** Jingjing Zhang, Quan Chen, Robert J. Mayer, Jin‐Dong Yang, Armin R. Ofial, Jin‐Pei Cheng, Herbert Mayr

**Affiliations:** ^1^ Center of Basic Molecular Science (CBMS) Department of Chemistry Tsinghua University Beijing 100084 P. R. China; ^2^ Department Chemie Ludwig-Maximilians-Universität München Butenandtstr. 5–13 81377 München Germany; ^3^ State Key Laboratory of Elemento-organic Chemistry College of Chemistry Nankai University Tianjin 300071 P. R. China

**Keywords:** diazoalkanes, electrophiles, kinetics, nucleophiles, organocatalysis

## Abstract

The kinetics and stereochemistry of the reactions of iminium ions derived from cinnamaldehydes and MacMillan's imidazolidinones with diphenyldiazomethane and aryldiazomethanes were investigated experimentally and with DFT calculations. The reactions of diphenyldiazomethane with iminium ions derived from MacMillan's second‐generation catalysts gave 3‐aryl‐2,2‐diphenylcyclopropanecarbaldehydes with yields >90 % and enantiomeric ratios of ≥90:10. Predominantly 2:1 products were obtained from the corresponding reactions with monoaryldiazomethanes. The measured rate constants are in good agreement with the rate constants derived from the one‐center nucleophilicity parameters *N* and *s*_N_ of diazomethanes and the one‐center electrophilicity parameters *E* of iminium ions as well as with quantum chemically calculated activation energies.

## Graphical abstract

**Better than a rough estimate**: The rates of Huisgen (3+2)‐cycloadditions of diazoalkanes with α,β‐unsaturated iminium ions can be predicted by Equation (1) using the tabulated one‐center nucleophilicities of diazoalkanes (*N*, *s*_N_) and the one‐center electrophilicity parameters *E* of the iminium ions. A rationalization for this observation is obtained by DFT calculations, which show that competing concerted and stepwise cycloadditions have similar Gibbs activation energies.

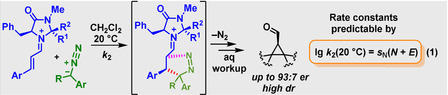

## Introduction

The prediction of rate constants for chemical reactions is of fundamental importance for designing synthetic transformations since their magnitude implies whether a certain reaction can be expected to take place under certain conditions. For this reason, the investigation of relationships between structures and rates of chemical reactions has been in the focus of research in physical organic chemistry for decades.^1^ Brønsted,^2^ Hammett,^3^ and Winstein–Grunwald^4^ correlations are among the best‐known relationships, which can be used to calculate unknown rate constants from known data within a reaction series. The applicability of these linear free energy correlations to cycloadditions is limited, however, and Frontier Orbital Theory has most commonly been employed to derive trends in cycloaddition rates.5a, 5b, 5c, 5d, 5e Though quantum chemical calculations nowadays allow one to calculate rates of organic reactions with high accuracy, they are rarely employed in early stages of synthesis planning, when new steps are usually designed heuristically5f by analogy with known reactions and not by time‐consuming calculations of reaction pathways.

In recent years, we have developed a set of one‐bond electrophilicities *E* and a set of one‐bond nucleophilicity parameters *N* and *s*_N_ for predicting rate constants for reactions of electrophiles with nucleophiles on the basis of Equation (1).^6^

(1) $$\mathrm{lg}\;k_{20{\;}^{\circ }C}=s{}_{N}\left(N+E\right)$$

Though Equation (1) has been developed for reactions, in which one and only one new bond is formed in the rate‐determining step, we have recently reported that it also predicts the rate constants for concerted cycloadditions that proceed with highly asynchronous bond formation.^7^

Huisgen reactions (1,3‐dipolar cycloadditions) represent the most general method for the synthesis of five‐membered heterocycles.^8^ Catalytic asymmetric versions have been developed in recent years,^9^ some of which proceed via chiral iminium ions or enamines.^10^ We have now investigated the kinetics of the reactions of iminium ions with diazoalkanes in order to examine whether the previously reported electrophilicity parameters of unsaturated iminium ions can assist the development of organocatalytic variants of Huisgen cycloadditions with electron‐rich 1,3‐dipoles.

## Results and Discussion

**Scheme 1:**
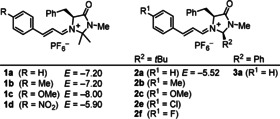
Iminium hexafluorophosphates used in this work (with electrophilicities *E* from ref. 12).

Iminium hexafluorophosphates (**1**–**3**)PF_6_ (Scheme 1) were obtained as crystalline salts by treatment of the corresponding imidazolidinonium hexafluorophosphates with cinnamaldehydes in methanol/dichloromethane solution at ambient temperature, following literature procedures.^11^

Combination of the iminium hexafluorophosphates **1 a**, **2 a**, or **3 a** with 1.5 equivalents of diphenyldiazomethane (**4**) in different solvents and subsequent workup with aqueous phosphate buffer gave 2,2,3‐triphenylcyclopropanecarbaldehyde (**5 a**)^13^ in variable yields and enantioselectivities. As shown in Table 1, the reaction of **1 a** with **4** afforded good yields of the cyclopropanecarbaldehyde **5 a** in dichloromethane, DMF, and acetonitrile, but not in methanol and THF solutions. Iminium ion **2 a**, derived from the MacMillan second‐generation catalyst, gave generally higher enantioselectivities than **1 a** and **3 a** (Table 1). Under comparable conditions, the enantioselectivities were slightly higher in acetonitrile than in dichloromethane (cf. entries 1 vs. 4 and 7 vs. 10) and increased when the reactions were carried out at lower temperatures (cf. entries 1 vs. 2 and 9 vs. 10).

**Table 1:** Reactions of iminium hexafluorophosphates **1 a**, **2 a**, and **3 a** with diphenyldiazomethane **4** ^[a]^ under different conditions. 
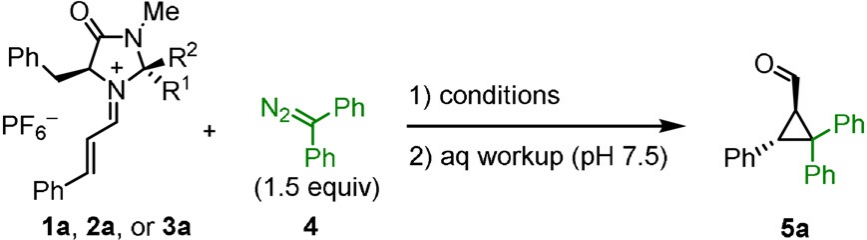

Entry	Iminium salt	Solvent	*T* (^o^C)	Time	Yield (%)^[b]^	*er* ^[c]^
1	**1 a**	CH_2_Cl_2_	r.t.	4 h	69	67:33
2	**1 a**	CH_2_Cl_2_	−20	24 h	55	70:30
3	**1 a**	DMF	r.t.	2 h	81	71:29
4	**1 a**	MeCN	r.t.	2 h	88	71:29
5	**1 a**	MeOH	r.t.	12 h	<10	–
6	**1 a**	THF	r.t.	12 h	<10	–
7	**2 a**	CH_2_Cl_2_	−40	48 h	60	87:13
8	**2 a**	CH_2_Cl_2_	−60	12 h	60	86:14
9	**2 a**	MeCN	r.t.	5 min	84	81:19
10	**2 a**	MeCN	−40	4 h	93	90:10
11	**3 a**	MeCN	−70	5 min	73	62:38

[a] Iminium hexafluorophosphates **1 a**, **2 a**, or **3 a** (0.20 mmol) and **4** (0.30 mmol) in 4 mL of solvent. [b] Yields of isolated products after purification by column chromatography. [c] Determined by chiral HPLC.

**Figure 1:**
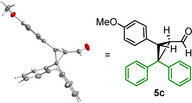
Single‐crystal X‐ray structure of **5 c** (ellipsoids are shown at the 20 % probability level).

Table 2 shows that variation of the 4‐substituents in the phenyl rings of the iminium ions **1** and **2** did not significantly affect the yields and enantioselectivities. All products were characterized by NMR spectroscopic methods and HRMS. The structure of cyclopropanecarbaldehyde **5 c** was furthermore confirmed by single‐crystal X‐ray structure analysis (Figure 1).

**Table 2:** Asymmetric cyclopropanation of iminium hexafluorophosphates **1** ^[a]^ and **2** ^[b]^ with diphenyldiazomethane **4**. 
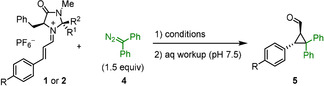

	Iminium ion	R	**5**	Yield (%)^[c]^	*er* ^[d]^	
	**1 a**	H	**5 a**	69	67:33	
	**1 b**	Me	**5 b**	60	65:35	
	**1 c**	OMe	**5 c**	72	68:32	
	**1 d**	NO_2_	**5 d**	58	69:31	
	**2 a**	H	**5 a**	93	90:10	
	**2 b**	Me	**5 b**	90	90:10	
	**2 c**	OMe	**5 c**	95	93:7	
	**2 e**	Cl	**5 e**	92	91:9	
	**2 f**	F	**5 f**	88	90:10

[a] Conditions: **1** (0.20 mmol) and **4** (0.30 mmol) in dichloromethane (4 mL) at 20 °C. [b] Conditions: **2** (0.20 mmol) and **4** (0.30 mmol) in acetonitrile (4 mL) at −40 °C. [c] Yields of isolated products after purification by column chromatography. [d] Determined by chiral HPLC.

**Scheme 2:**
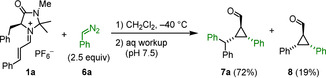
Reaction of **1 a** with phenyldiazomethane (**6 a**).

As depicted in Scheme 2, the reactions of iminium ions with monoaryldiazomethanes took another course. Only 19 % yield of the 1:1‐product **8** was obtained, while the major product **7 a** was formed from a reaction of iminium ion **1 a** with two molecules of phenyldiazomethane (**6 a**). Since the ratio **7 a**/**8** did not change during the reaction, and use of equimolar amounts of the reactants just reduced the overall yield, we can exclude that **7 a** was formed by the reaction of the 1:1 product **8** with **6 a**. While **8** has been described previously in the literature,^14^ the structure of **7 a** was assigned by comparison of its NMR spectra with those of **7 b**, for which crystallographic data are available (see below).

**Figure 2:**
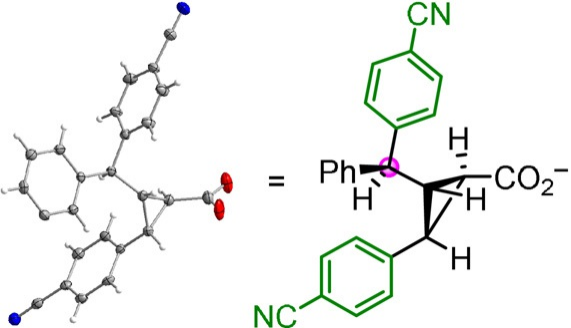
Single‐crystal X‐ray structure of the potassium cyclopropanecarboxylate **9** obtained by oxidation of **7 b** (K^+^ counterion omitted for clarity; thermal ellipsoids are shown at the 50 % probability level).

**Scheme 3:**
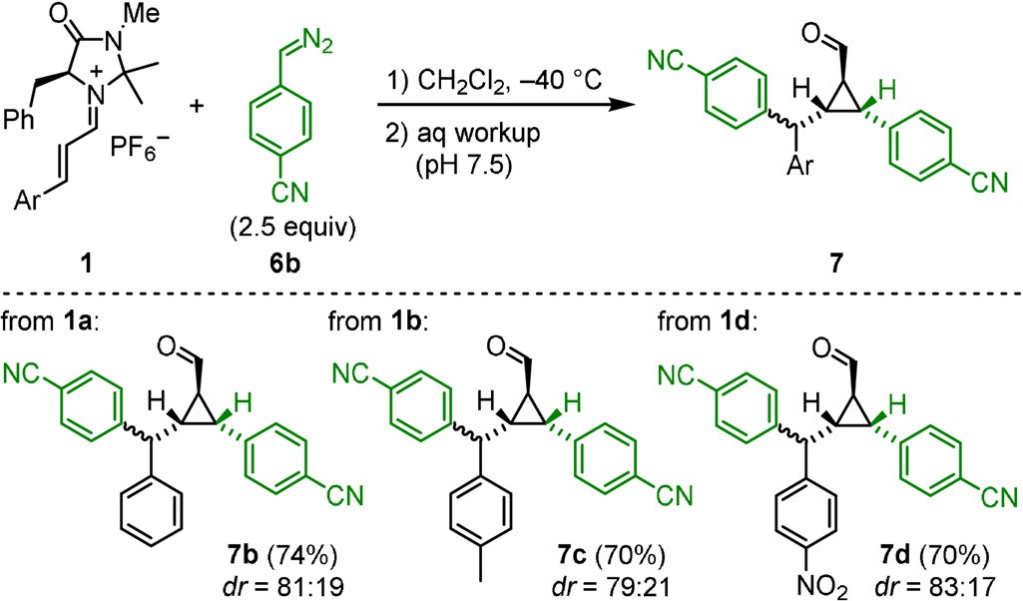
Reactions of iminium hexafluorophosphates **1** with (4‐cyanophenyl)diazomethane **6 b**.

Treatment of other iminium hexafluorophosphates **1** with (4‐cyanophenyl)diazomethane (**6 b**) under the same conditions led to the exclusive formation of the 2:1 products **7 b**–**d**, while not even traces of 1:1 products were detected (Scheme 3). In order to unequivocally assign the structures of the 2:1 products **7**, aldehyde **7 b** was oxidized with a mixture of NaClO_2_/NaH_2_PO_4_ under phase‐transfer conditions (isopentane/water) to yield the corresponding carboxylic acid, whose potassium salt **9** gave crystals suitable for X‐ray structure analysis (Figure 2).

**Figure 3:**
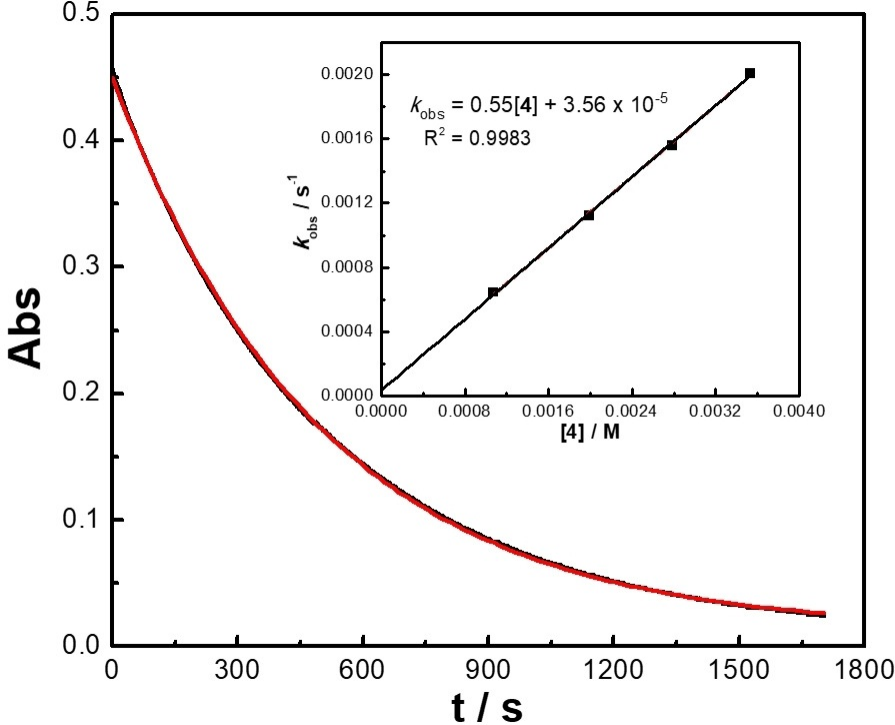
Monoexponential time‐dependent decay of the absorbance (Abs, at 400 nm) for the reaction of **2 a** (2.87×10^−5^ m) with **4** (3.53×10^−3^ m) in dichloromethane at 20 °C. Inset: Correlation of *k*_obs_ (s^−1^) with the concentrations of **4**.

The kinetics of the reactions of the iminium ions **1** and **2 a** with diphenyldiazomethane (**4**) and the monoaryldiazomethanes **6** were determined photometrically by monitoring the disappearance of the colored iminium ions **1** and **2 a** in dichloromethane at 20 °C under pseudo‐first‐order conditions using >10 equiv of the diazomethanes **4** and **6**, following previously described procedures.^12^ As illustrated for the reaction of **2 a** with **4** in Figure 3, the first‐order rate constant *k*_obs_ (s^−1^) was derived from the exponential decay of the UV/Vis absorption of the iminium ion **2 a** at 400 nm. The inset of Figure 3 shows that the second‐order rate constant *k*_2_^exp^ (m ^−1^ s^−1^) is given by the slope of the plot of *k*_obs_ (s^−1^) vs. the concentration of **4**. The same method was used for determining the second‐order rate constants for the reactions with the monoaryldiazomethanes **6**. Since the diazoalkanes **6** were always used in high excess, the evaluation of the kinetic measurements was not affected by the fact that two equivalents of **6** were consumed per iminium ion.

Table 3 compares the resulting second‐order rate constants *k*_2_^exp^ with the rate constants *k*_2_^eq1^, which were calculated by Equation (1) from the previously determined one‐center electrophilicities *E* (Scheme 1) and the one‐center nucleophilicity parameters *N* and *s*_N_ (Table 3, left column). As shown in the right column of Table 3, the agreement between experimental rate constants and predictions by Equation (1) is similar to that for electrophile–nucleophile combinations in which only one new bond is formed in the rate‐determining step.^6^ In order to elucidate the reason for this remarkable agreement, we performed DFT calculations at the (SMD=DCM)//B3LYP‐D3(BJ)/def2‐SVP level of theory.^16^

**Table 3:** Experimental (*k*_2_^exp^) and calculated (*k*_2_^eq1^) second‐order rate constants for the reactions of iminium ions **1** and **2 a** with diazomethanes **4** and **6 a**–**c** (CH_2_Cl_2_, 20 °C).

R(R^1^)CN_2_^[a]^	Iminium ion	*k*_2_^exp^ (m ^−1^ s^−1^)	*k*_2_^eq1^ (m ^−1^ s^−1^)	*k*_2_^exp^/*k*_2_^eq1^
Ph_2_CN_2_ (**4**)	**1 a**	1.48×10^−1^	1.75×10^−2^	8.5
*N*=5.29, *s*_N_=0.92	**1 b**	6.48×10^−2^	1.75×10^−2^	3.7
	**1 c**	1.76×10^−2^	3.21×10^−3^	5.5
	**1 d**	4.73×10^−1^	2.75×10^−1^	1.7
	**2 a**	5.54×10^−1^	6.14×10^−1^	0.90
				
PhCHN_2_ (**6 a**)	**1 a**	2.07×10^3^	6.09×10^1^	34
*N*=9.35, *s*_N_=0.83	**1 b**	6.11×10^2^	6.09×10^1^	10
	**1 c**	1.35×10^2^	1.32×10^1^	10
				
(4‐NC‐C_6_H_4_)CHN_2_ (**6 b**)	**1 a**	2.69×10^1^	2.33	12
*N*=7.66, *s*_N_=0.80	**1 b**	1.51×10^1^	2.33	6.5
	**1 c**	2.94	5.35×10^−1^	5.5
				
(4‐Br‐C_6_H_4_)CHN_2_ (**6 c**)	**1 a**	4.56×10^2^	2.34×10^1^	19
*N*=8.87, *s*_N_=0.82	**1 b**	1.98×10^2^	2.34×10^1^	8.5
	**1 c**	4.70×10^1^	5.17	9.1

[a] *N* and *s*_N_ from refs. 7, 15

**Figure 4:**
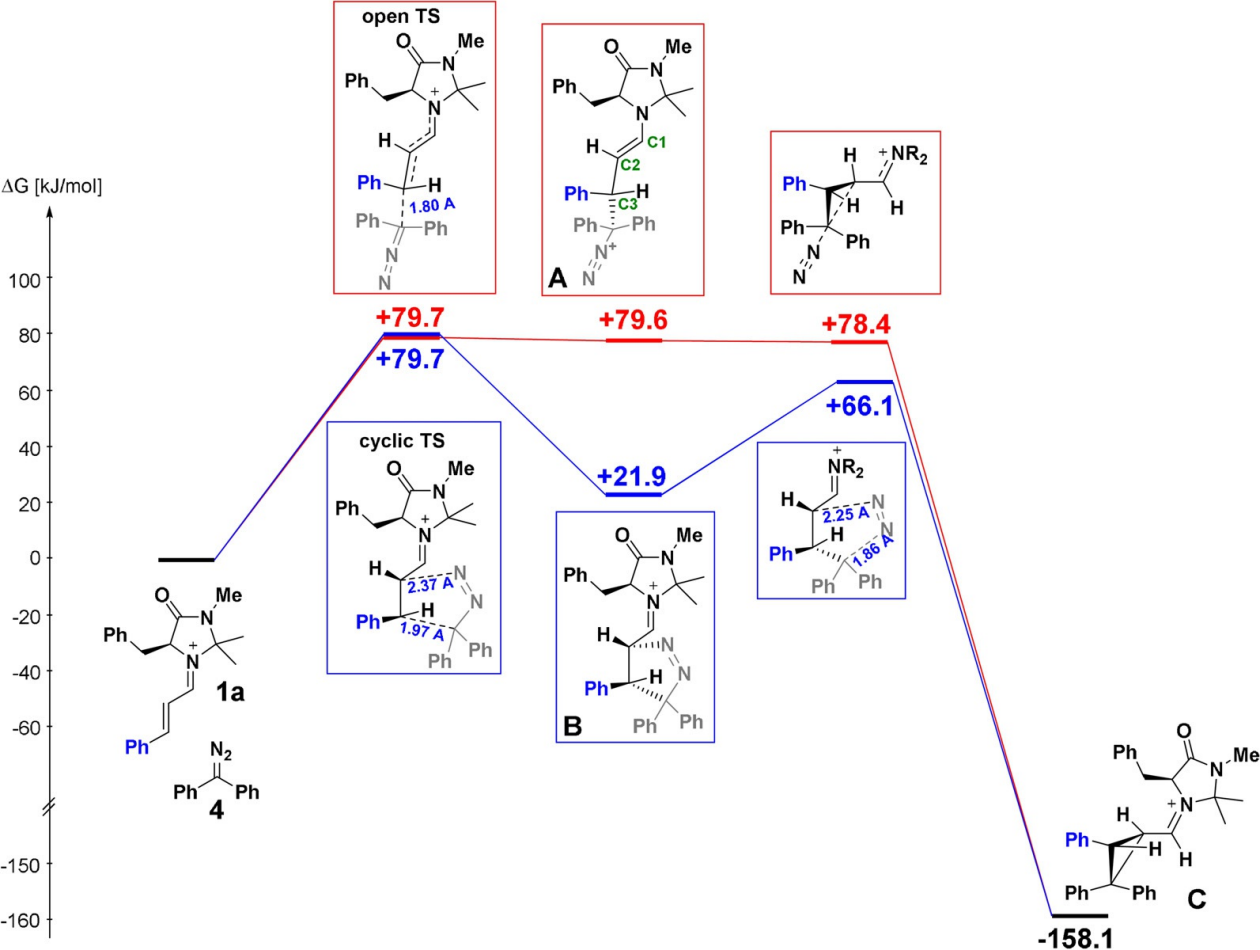
Gibbs energy profile for the reaction of the iminium ion **1 a** with diphenyldiazomethane (**4**) at the (SMD=DCM)//B3LYP‐D3(BJ)/def2‐SVP level of theory.

Figure 4 shows the attack of diphenyldiazomethane (**4**) at the bottom face of the iminium ion **1 a**, the well‐known preferred site of nucleophilic attack at **1 a**.^17^ Two reaction pathways are depicted: The reaction via an open transition state (red) leads to diazonium ion **A**, an intermediate on a very shallow hypersurface, which subsequently undergoes an intramolecular nucleophilic substitution with loss of nitrogen and formation of cyclopropane **C**. The alternative path (blue) yields the Δ^1^‐pyrazoline **B** through a concerted cycloaddition with the same barrier as that for the path in which only one new bond is formed in the transition state (red).

The low activation energy for nitrogen expulsion from **B** yielding cyclopropane **C** explains why hydrolysis products of the pyrazoline **B** have not been observed. Both pathways lead to the same stereoisomer **C**, in agreement with the experimentally observed structure of **5 c** (Figure 1). Figure S4 (Supporting Information) shows that 180° rotation around the C2−C3 bond in **A** and subsequent cyclization with cyclopropane formation proceeds through a transition state that is 13 kJ mol^−1^ higher in energy than that for the direct cyclization of **A**, in line with the fact that stereoisomers of **5 a**–**5 d** with formyl and aryl group in *cis*‐position were not observed (Table 2).

The calculated Gibbs activation energies for both pathways (79.7 kJ mol^−1^) are in good agreement with the experimentally determined Δ*G* ^≠^_exp_=76.4 kJ mol^−1^ (from Table 3) as well as with the activation energy derived from the one‐bond reactivity parameters *E*, *N*, and *s*_N_ (Δ*G* ^≠^=81.6 kJ mol^−1^, from Table 3). Correlation (1) is thus suitable to calculate absolute values for the second‐order rate constants of these cycloadditions, but does not differentiate stepwise from concerted cycloadditions with highly asynchronous bond formation.

**Figure 5:**
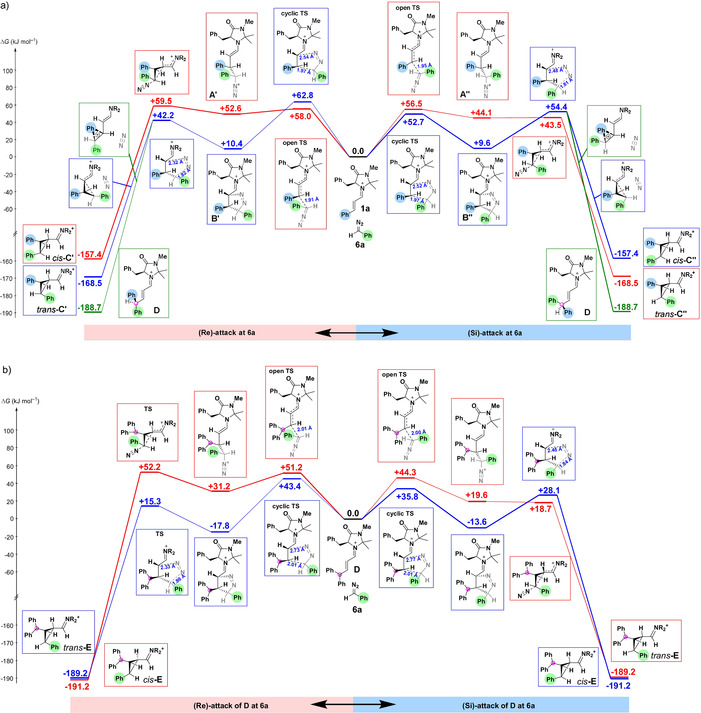
Gibbs energy profile for the reactions of iminium ion **1 a** (a) and of iminium ion **D** (b) with phenyldiazomethane (**6 a**) at the (SMD=DCM)//B3LYP‐D3(BJ)/def2‐SVP level of theory.

In contrast to diphenyldiazomethane (**4**), phenyldiazomethane (**6 a**) has two heterotopic faces, and the left part of Figure 5 a describes the *Re*‐attack at **6 a**, while the right part shows the *Si*‐attack. As in the reactions with **4** (Figure 4), the pathways via open transition states, which yield the diazonium ions **A′** and **A′′**, are marked in red, while the paths via cyclic transition states, which yield the Δ^1^‐pyrazolines **B′** and **B′′**, are labeled in blue. The similar lengths of the new CC bonds in the transition states of the stepwise (red, 1.91 and 1.95 Å) and concerted cycloadditions (blue, more advanced bond=1.97 Å) and the comparable activation energies again show the close similarity of both pathways. Though the energy differences are very small, Figure 5 a suggests that the concerted pathway (blue) to give pyrazoline **B′′** should be preferred in the case of *Si*‐attack (Δ*G* ^≠^=52.7 kJ mol^−1^, right side of Figure 5 a), while the stepwise process with formation of diazonium ion **A′** should be kinetically favored in the case of the *Re*‐attack (Δ*G* ^≠^=58.0 kJ mol^−1^, left side of Figure 5 a).

The next steps differ from those in Figure 4. Whereas intermediates **A** and **B** obtained from diphenyldiazomethane (**4**) are exclusively converted into the cyclopropane **C**, N_2_‐elimination from the pyrazolines **B′** and **B′′** obtained from phenyldiazomethane (**6 a**) proceeds predominantly with phenyl migration to give the conjugated iminium ion **D**, while cyclopropane formation represents the minor pathway.

According to Figure 5 a, the blue pathway on the right, which yields *cis*‐**C′′**, is the energetically most favorable of the four cyclopropane‐forming processes, in line with the isolation of cyclopropane **8** (Scheme 2) with the two phenyl groups in *cis* position.

Figure 5 b explains why hydrolysis products of iminium ion **D** were not observed. Iminium **D** is generated in the presence of phenyldiazomethane (**6 a**), and reacts with **6 a** much faster than iminium ion **1 a**. This is true for all four reaction pathways of **D**, *Re*‐ and *Si*‐attack, open and cyclic transition states.

Among these pathways, *Si*‐attack via a cyclic transition state (blue path, right in Figure 5 b) is kinetically preferred. Since iminium ion **D** is not stabilized by phenyl conjugation as its precursor **1 a**, the reaction of **6 a** with **D** is much more exergonic than the corresponding reaction with **1 a** (−13.6 vs. +9.6 kJ mol^−1^), and 73 % of this difference in reaction Gibbs energies is reflected by the Gibbs activation energies (35.8 vs. 52.7 kJ mol^−1^). As a consequence, iminium ion **D**, once formed through phenyl migration from **B′′**, reacts immediately with a second molecule of the nucleophilic diazo compound **6 a** and thus accounts for the predominant formation of 2:1 products.

According to this analysis, the rate‐determining step for the formation of the 2:1 products **7**, hydrolysis products of *cis*‐**E**, is the formation of **B′′** (Δ*G* ^≠^=52.7 kJ mol^−1^, Figure 5 a) or the N_2_ elimination from **B′′** (Δ*G* ^≠^=54.4 kJ mol^−1^ relative to reactants **1 a** and **6 a**), again in excellent agreement with the experimental value (Δ*G* ^≠^=53.1 kJ mol^−1^, from Table 3).

Let us now consider the reaction of iminium ion **1 a** with (4‐cyanophenyl)diazomethane (**6 b**) which gave **7 b** as the major stereoisomer (Scheme 3 and Figure 2). The stereoselectivity of the formation of **7 b** can be rationalized by replacing the green phenyl group in Figure 5 by a 4‐cyanophenyl group. The benzhydryl carbon now becomes a center of chirality (marked by red circles) with (*S*)‐configuration in iminium ion **D** on the bottom right of Figure 5 a and (*R*)‐configuration in the corresponding structure **D** on the bottom left. The (*S*)‐configuration of this carbon in the carboxylate **9** derived from aldehyde **7 b** (Figure 2) again confirms the preferred operation of the blue pathway in Figure 5 a, right, i.e., concerted cycloaddition with *Si*‐attack.

While the concerted cycloaddition with *Si*‐attack at **6 a** is only slightly preferred in the reaction with iminium ion **1 a** (Figure 5 a), it is the clearly preferred pathway in the reaction with iminium ion **D** (Figure 5 b, blue pathway, right). The resulting *cis*‐position of the phenyl and benzhydryl groups in *cis*‐**E** is in line with the observed configuration in the isolated cyclopropanes **7 a**–**7 d** (Scheme 3).

As described in Section 8 of the Supporting Information, attempts to perform these cyclopropanations under organocatalytic conditions with MacMillan's imidazolidinones as catalysts have failed so far, because of deprotonation (i.e., deactivation) of the imidazolidinonium ions by the diazoalkanes. Further attempts to realize enantioselective Huisgen reactions with organocatalysts of higher p*K*_aH_ are presently under investigation.

## Conclusion

The three‐parameter Equation (1), which has been derived for reactions of electrophiles with nucleophiles, in which only one new bond is formed in the rate‐determining step,^6^ has now been shown also to predict absolute rate constants for Huisgen cycloadditions of iminium ions with diazoalkanes. The agreement between calculated and experimental rate constants with a maximum deviation of factor 34 is amazing in view of the 40 orders of magnitude covered by Equation (1). DFT calculations show that stepwise and concerted cycloadditions of these reactants proceed with similar activation energies, which explains why the one‐center electrophilicities *E* and the one‐center nucleophilicity parameters *N* and *s*_N_ 18 are also applicable to concerted cycloadditions that proceed with highly asynchronous bond formation.

## Conflict of interest

The authors declare no conflict of interest.

## Supporting information

As a service to our authors and readers, this journal provides supporting information supplied by the authors. Such materials are peer reviewed and may be re‐organized for online delivery, but are not copy‐edited or typeset. Technical support issues arising from supporting information (other than missing files) should be addressed to the authors.

Supplementary

Supplementary
