# Supplementary material for: Predicting Absolute Rate Constants for Huisgen Reactions of Unsaturated Iminium Ions with Diazoalkanes
Source: Angew Chem Int Ed Engl. 2020 May 11;59(30):12527–33. doi: 10.1002/anie.202003029 (PMC7383640; doi:10.1002/anie.202003029)
Supplement: Supplementary file 1 — Supplementary [file ANIE-59-12527-s001.pdf]

Angewandte Chemie
Eine Zeitschrift der Gesellschaft Deutscher Chemiker

Supporting Information

Predicting Absolute Rate Constants for Huisgen Reactions of Unsaturated Iminium Ions with Diazoalkanes

*Jingjing Zhang+, Quan Chen+, Robert J. Mayer+, Jin-Dong Yang, Armin R. Ofial, Jin-Pei Cheng, and Herbert Mayr**

anie_202003029_sm_miscellaneous_information.pdf
anie_202003029_sm_miscellaneous_information.zip

Author Contributions

R.J.M. performed all quantum chemical calculations.

Supporting Information

Content

1. General Information S2
2. Synthesis of Iminium Hexafluorophosphates **2** S3
3. Reactions of Iminium Hexafluorophosphates with Diphenyldiazomethane (**4**) S5
4. Cyclopropanation of Iminium Hexafluorophosphates **1** with Diphenyldiazomethane (**4**) S5
5. Cyclopropanation of Iminium Hexafluorophosphates **2** with Diphenyldiazomethane (**4**) S7
6. Reactions of Iminium Hexafluorophosphates **1** with Aryldiazomethanes **6** S11
7. Oxidation of **7b** S14
8. Attempts to Catalyze the Reactions of Cinnamaldehyde with Aryldiazomethanes by MacMillan Catalysts S14
9. Kinetics of the Reactions of Iminium Salts with Aryldiazomethanes S15
10. Computational Details S19
11. Copies of NMR Spectra S34
12. Crystallographic Data S49
13. References S52

1. General Information

Chemicals: The iminium hexafluorophosphates **1-3** were prepared by following reported methods through condensation of α,β-unsaturated aldehydes with imidazolidinones.[S 1] Aryldiazomethanes **4** and **6**(**a-c**) were synthesized as described in the literature.[S 2] Dichloromethane and acetonitrile were purchased from J&K Chemical (99.9 %, extra dry, water < 30 ppm, J&K seal) and used without further purification. Other solvents were distilled by standard solvent treatment methods. Unless otherwise mentioned, all chemicals were purchased from commercial sources. Flash chromatography was performed to purify the products. Reaction temperature refers to temperature of an aluminum heating block or a silicon oil bath, which was controlled by an electronic temperature modulator from IKA.

Analysis: NMR spectra were acquired on NMR spectrometers with 400 or 600 MHz for ^{1}H NMR and 101 MHz for ^{13}C NMR. Chemical shifts (δ) were reported in ppm relative to the residual solvent signal. Data for ^{1}H NMR spectra were reported as follows: chemical shift (multiplicity, coupling constants, number of hydrogens). Abbreviations were as follows: s (singlet), d (doublet), t (triplet), q (quartet), m (multiplet), br (broad). The aromatic NMR signals of **7a**, **7b**, and **8** were assigned by two-dimensional NMR spectroscopy (HSQC, HMBC, COSY). High-resolution mass spectral data were performed on a Thermo Scientific Q Exactive (positive mode) at the Mass Spectrometry Facility. Enantiomeric ratios (er) were determined by analytical liquid chromatography (HPLC) analysis on a Shimadzu chromatograph (Daicel chiral columns Chiralpak IA, IC, ID, IE, IF (4.6 × 250 mm)).

Kinetics: The rates of the reactions were determined spectrophotometrically using stopped-flow (Applied Photophysics SX.18MV-R and Hi-Tech SF-61DX2) and diode array spectometer systems (J&M TIDAS DAD 2062) by following the disappearance of the iminium ions **1**–**3** in CH_2Cl_2. The temperature of the solutions was kept constant (20.0 ± 0.1 °C) by using a circulating bath thermostat. All kinetic investigations were performed with a high excess of the nucleophiles **4** and **6** over the iminium ions **1**–**3** to achieve first-order kinetics (for example, [**4**]/[**1**] > 10). Rate constants k_{obs} (s^{-1}) were obtained by fitting the single exponential decay $A_t = A_0\exp(-k_{obs}t) + C$ to the observed time-dependent absorbance. Second-order rate constants k_2 (M^{-1} s^{-1}) were derived from the slopes of the linear correlations of k_{obs} (s^{-1}) with concentrations of the aryldiazomethanes **4** and **6**.

2. Synthesis of Iminium Hexafluorophosphates 2

Solutions of the corresponding cinnamaldehydes (15 mmol in 4 mL of CH_2Cl_2) were added dropwise at ambient temperature to solutions of (2*S*,5*S*)-5-benzyl-2-(*tert*-butyl)-2,3-dimethylimidazolidin-4-one·HPF_6 (3.90 g, 10.0 mmol) in methanol (15 mL). The mixtures were stirred for 2 h, and the precipitates were isolated by filtration, washed with cold methanol (10.0 mL), diethylether (20 mL), and dried in the vacuum for 12 h.

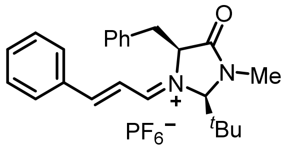

2a

2a: Yield: 91%. **^{1}H NMR** (400 MHz, CD_3CN) δ 8.47 (d, J = 10.9 Hz, 1 H), 8.03 (d, J = 15.0 Hz, 1 H), 7.73–7.65 (m, 1 H), 7.59–7.41 (m, 8 H), 7.35–7.25 (m, 1 H), 6.27 (dd, J = 15.0, 10.8 Hz, 1 H), 5.26 (s, 1 H), 4.89 (dd, J = 8.9, 4.8 Hz, 1 H), 3.58 (dd, J = 14.9, 4.8 Hz, 1 H), 3.38–3.17 (m, 1 H), 3.12 (s, 3 H), 1.27 (s, 9 H). **^{13}C NMR** (101 MHz, CD_3CN) δ 171.9, 167.1, 166.3, 136.1, 135.3, 133.0, 131.3, 129.8, 129.5, 129.5, 128.2, 116.8, 90.3, 64.7, 37.5, 37.3, 31.6, 25.9. **HRMS (ESI)** calcd for $C_{24}H_{29}N_2O^+$ (M^+) 361.2274, found 361.2265.
NMR spectroscopic data are in agreement with those in the literature.[S1c,d]

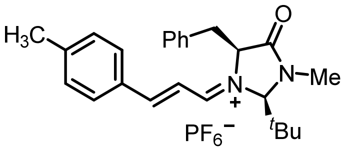

2b

2b: Yield: 87%. **^{1}H NMR** (400 MHz, CD_3CN) δ 8.42 (d, J = 10.9 Hz, 1 H), 8.00 (d, J = 14.9 Hz, 1 H), 7.50–7.46 (m, 4 H), 7.42–7.35 (m, 4 H), 7.30 (t, J = 7.2 Hz, 1 H), 6.21 (dd, J = 14.9, 10.9 Hz, 1 H), 5.23 (s, 1 H), 4.84 (dd, J = 8.8, 4.8 Hz, 1 H), 3.57 (dd, J = 14.9, 4.8 Hz, 1 H), 3.26 (dd, J = 14.9, 8.9 Hz, 1 H), 3.11 (s, 3 H), 2.46 (s, 3 H), 1.26 (s, 9 H). **^{13}C NMR** (101 MHz, CD_3CN) δ 171.5, 167.2, 166.5, 147.8, 136.2, 131.6, 130.6, 130.3, 129.8, 129.4, 128.2, 115.7, 90.1, 64.6, 37.6, 37.3, 31.6, 25.8, 21.2. **HRMS (ESI)** calcd for $C_{25}H_{31}N_2O^+$ (M^+) 375.2431, found 375.2425.

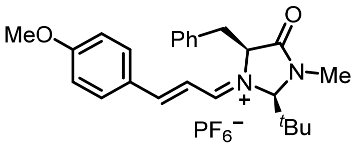

2c

2c: Yield: 92%. **^{1}H NMR** (400 MHz, CD_3CN) δ 8.32 (d, J = 11.1 Hz, 1 H), 7.95 (d, J = 14.7 Hz, 1 H), 7.55–7.42 (m, 6 H), 7.32 (t, J = 7.2 Hz, 1 H), 7.08 (d, J = 8.9 Hz, 2 H), 6.06 (dd, J = 14.7, 11.0 Hz, 1 H), 5.20 (s, 1 H), 4.79 (dd, J = 8.9, 4.7 Hz, 1 H), 3.95 (s, 3 H), 3.55 (dd, J = 14.9, 4.7 Hz, 1 H), 3.24 (dd, J = 14.9, 9.0 Hz, 1 H), 3.10 (s, 3 H), 1.25 (s, 9 H). **^{13}C NMR** (101 MHz, CD_3CN) δ 170.6, 167.5, 166.3, 166.1, 136.4, 134.5, 129.8, 129.4, 128.1, 126.1, 115.4, 113.8, 89.9, 64.4, 56.0, 37.7, 37.2, 31.6, 25.8. **HRMS (ESI)** calcd for $C_{25}H_{31}N_2O_2^+$ (M^+) 391.2380, found 391.2368.

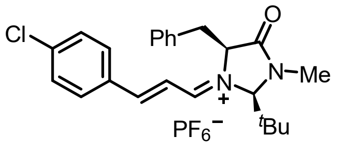

2e

2e: Yield: 90%. **^{1}H NMR** (400 MHz, CD_3CN) δ 8.46 (d, *J* = 10.8 Hz, 1 H), 7.97 (d, *J* = 15.0 Hz, 1 H), 7.57 (d, *J* = 8.6 Hz, 2 H), 7.49–7.43 (m, 6 H), 7.29 (t, *J* = 7.2 Hz, 1 H), 6.20 (dd, *J* = 15.0, 10.8 Hz, 1 H), 5.26 (s, 1 H), 4.88 (dd, *J* = 9.1, 4.7 Hz, 1 H), 3.58 (dd, *J* = 14.9, 4.7 Hz, 1 H), 3.26 (dd, *J* = 14.9, 9.2 Hz, 1 H), 3.11 (d, *J* = 6.6 Hz, 3 H), 1.26 (s, 9 H). **^{13}C NMR** (101 MHz, CD_3CN) δ 171.9, 167.0, 164.3, 140.9, 136.0, 132.6, 131.7, 129.9, 129.8, 129.5, 128.3, (117.3, superimposed by the CD_3CN resonance), 90.4, 64.7, 37.5, 37.3, 31.7, 25.9. **HRMS (ESI)** calcd for $C_{24}H_{28}ClN_2O^+$ (M^+) 395.1885, found 395.1880.

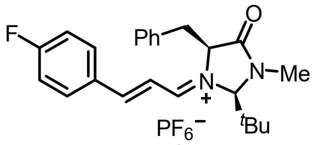

2f

2f: Yield: 75%. **^{1}H NMR** (400 MHz, CD_3CN) δ 8.45 (d, *J* = 10.8 Hz, 1 H), 8.00 (d, *J* = 15.0 Hz, 1 H), 7.54–7.27 (m, 9 H), 6.16 (dd, *J* = 15.0, 10.8 Hz, 1 H), 5.26 (s, 1 H), 4.87 (dd, *J* = 9.0, 4.7 Hz, 1 H), 3.58 (dd, *J* = 14.9, 4.8 Hz, 1 H), 3.27 (dd, *J* = 14.9, 9.1 Hz, 1 H), 3.11 (s, 3 H), 1.26 (s, 9H). **^{13}C NMR** (101 MHz, CD_3CN) δ 171.8, 167.1, 166.7 (d, *J* = 259 Hz), 164.7, 136.1, 134.2 (d, *J* = 10.1 Hz), 129.9, 129.5, 128.2, 117.0, 116.8, 116.6 (d. *J* = 2.0 Hz), 90.3, 64.7, 37.5, 37.3, 31.6, 25.9. **HRMS (ESI)** calcd for $C_{24}H_{28}FN_2O^+$ (M^+) 379.2180, found 379.2174.

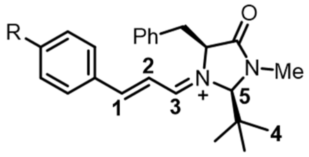

^{1}H NMR	H_1	H_2	H_3	H_4	H_5
**2a**	8.03	6.27	8.47	1.27	5.26
**2b**	8.00	6.21	8.42	1.26	5.23
**2c**	7.95	6.06	8.32	1.25	5.20
**2e**	7.97	6.20	8.46	1.26	5.26
**2f**	8.00	6.16	8.45	1.26	5.26

Figure S1. The summary of assignments of partial ^{1}H NMR signals for iminiums ions **2**.

3. Reactions of Iminium Hexafluorophosphates with Diphenyldiazomethane (4)

Solutions of diphenyldiazomethane **4** (0.30 mmol in 2 mL of different solvents) were slowly added to solutions of the iminium hexafluorophosphates **1a**, **2a**, or **3a** (0.20 mmol in 2 mL of the same solvent) at the temperature specified in the following Table. The solutions were stirred for the indicated time at the specified temperature before aqueous phosphate buffer solution (pH 7.5) was added. After stirring for 30 min, the solutions were extracted with CH_2Cl_2 (3 × 3 mL) and dried over $MgSO_4$. After evaporation of the solvents under reduced pressure, the cyclopropane **5a** was purified by column chromatography (silica gel, EtOAc/hexane 2/100 v/v). Table 1 (main text) lists the results of the individual experiments.

4. Cyclopropanation of Iminium Hexafluorophosphates 1 with Diphenyldiazomethane (4)

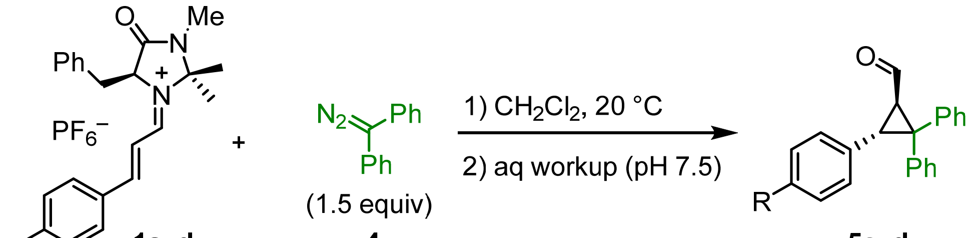

Solutions of diphenyldiazomethane **4** (0.30 mmol in 2 mL of CH_2Cl_2) were slowly added to solutions of the iminium hexafluorophosphates **1a**–**d** (0.20 mmol in 2 mL of CH_2Cl_2) at ambient temperature and stirred for 4 h.
Then, aqueous phosphate buffer solution (5 mL, pH 7.5) was added, and the mixture was stirred for 30 min. The mixture was extracted with CH_2Cl_2 (3 × 3 mL) and dried over $MgSO_4$. After evaporation of the solvents under reduced pressure, the products **5** were purified by column chromatography (silica gel, EtOAc/hexane 2/100 v/v).

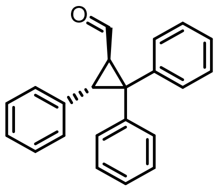

5a

2,2,3-Triphenylcyclopropane-1-carbaldehyde (**5a**): Yield 69%. **^{1}H NMR** (400 MHz, $CDCl_3$) δ 8.90 (d, J = 6.5 Hz, 1 H), 7.50 (d, J = 8.4 Hz, 2 H), 7.33 (t, J = 7.6 Hz, 2 H), 7.23 (t, J = 7.4 Hz, 1 H), 7.18–7.04 (m, 8 H), 6.95–6.93 (m, 2 H), 3.76 (d, J = 5.7 Hz, 1 H), 3.15–3.12 (m, 1 H). **^{13}C NMR** (101 MHz, $CDCl_3$) δ 199.9, 141.4, 139.6, 135.1, 129.5, 129.4, 129.0, 128.4, 128.1, 128.1, 127.4, 126.9, 126.7, 48.7, 40.9, 35.5. **HRMS (ESI)** calcd for $C_{22}H_{19}O^+$ (M + H^+) 299.1430, found 299.1423.

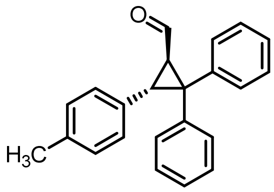

5b

2,2-Diphenyl-3-(*p*-tolyl)cyclopropane-1-carbaldehyde (**5b**): Yield 60%. **^{1}H NMR** (400 MHz, $CDCl_3$) δ 8.87 (d, *J* = 6.6 Hz, 1 H), 7.49 (d, *J* = 7.3 Hz, 2 H), 7.34–7.30 (m, 2 H), 7.22 (t, *J* = 7.4 Hz, 1 H), 7.15–7.05 (m, 5 H), 6.95 (d, *J* = 7.9 Hz, 2 H), 6.81 (d, *J* = 8.0 Hz, 2 H), 3.72 (d, *J* = 5.7 Hz, 1 H), 3.10–3.07 (m, 1 H), 2.24 (s, 3 H). **^{13}C NMR** (101 MHz, $CDCl_3$) δ 200.1, 141.5, 139.7, 136.3, 131.9, 129.52, 129.46, 129.0, 128.8, 128.4, 127.9, 127.4, 126.9, 48.6, 41.2, 35.4, 21.0. **HRMS (ESI)** calcd for $C_{23}H_{21}O^+$ (M + H$^+$) 313.1587, found 313.1578.

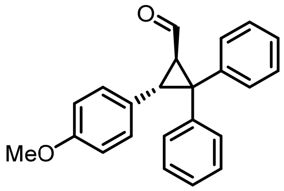

5c

3-(4-Methoxyphenyl)-2,2-diphenylcyclopropane-1-carbaldehyde (**5c**): Yield 72%. **^{1}H NMR** (400 MHz, $CDCl_3$) δ 8.86 (d, *J* = 6.5 Hz, 1 H), 7.49 (d, *J* = 7.3 Hz, 2 H), 7.34–7.30 (m, 2 H), 7.22 (t, *J* = 7.4 Hz, 1 H), 7.17–7.04 (m, 5 H), 6.85 (d, *J* = 8.6 Hz, 2 H), 6.68 (d, *J* = 8.7 Hz, 2 H), 3.72–3.70 (m, 4 H), 3.07–3.04 (m, 1 H). **^{13}C NMR** (101 MHz, $CDCl_3$) δ 200.1, 158.4, 141.5, 139.7, 129.5, 129.4, 129.1, 129.0, 128.4, 127.3, 127.0, 126.8, 113.5, 55.2, 48.4, 41.2, 35.0. **HRMS (ESI)** calcd for $C_{23}H_{21}O_2^+$ (M + H$^+$) 329.1536, found 329.1538.

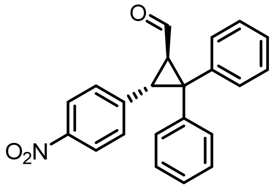

5d

3-(4-Nitrophenyl)-2,2-diphenylcyclopropane-1-carbaldehyde (**5d**): Yield 58%. **^{1}H NMR** (400 MHz, $CDCl_3$) δ 8.99 (d, *J* = 8.0 Hz, 1 H), 8.03 (d, *J* = 8.0 Hz, 2 H), 7.51–7.49 (m, 2 H), 7.39–7.37 (m, 2 H), 7.35–7.26 (m, 1 H), 7.18–7.09 (m, 7 H), 3.85 (d, *J* = 5.7 Hz, 1 H), 3.27–3.24 (m, 1 H). **^{13}C NMR** (101 MHz, $CDCl_3$) δ 198.6, 146.7, 143.2, 140.4, 138.6, 129.3, 129.2, 129.1, 128.8, 128.7, 127.8, 127.5, 123.3, 48.6, 41.2, 34.8. **HRMS (ESI)** calcd for $C_{22}H_{18}NO_3^+$ (M + H$^+$) 344.1281, found 344.1284.

5. Cyclopropanation of Iminium Hexafluorophosphates 2 with Diphenyldiazomethane (4)

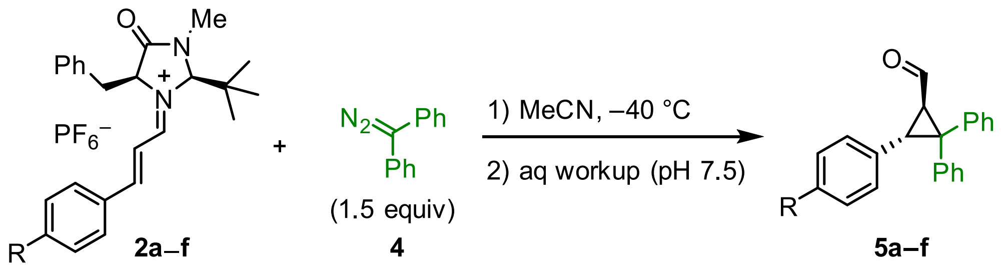

Precooled solutions of diphenyldiazomethane **4** (0.30 mmol in 2 mL of MeCN) were slowly added to solutions of the iminium hexafluorophosphates **2a–f** (0.20 mmol in 2 mL of MeCN) at -40 °C and stirred for 4 h. Then, aqueous phosphate buffer solution (5 mL, pH 7.5) was added and stirred for 30 min. The mixture was extracted with CH_2Cl_2 (3 × 3 mL) and dried over $MgSO_4$. After evaporation of the solvents under reduced pressure, the products **5a–f** were purified by column chromatography (silica gel, EtOAc/hexane 2/100 v/v). NMR spectroscopic analysis of the products showed the exclusive formation of the cyclopropanes **5** with *trans* configuration.

For an unequivocal interpretation of the chromatograms, analogous reactions were performed using racemic mixtures of the iminium hexafluorophosphates *rac*-**2a–f** to synthesize *rac*-**5a–f**.

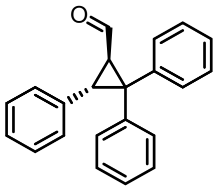

5a

5a: Yield 93%.

The enantiomeric purity of **5a** was determined by HPLC analysis in comparison with racemic product (Chiralpak ID, 100:1 hexane: *i*-PrOH, 1.0 mL/min, 220 nm): t_{minor} = 17.5 min, t_{major} = 18.7 min.

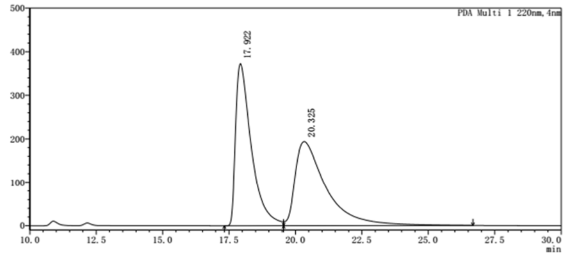

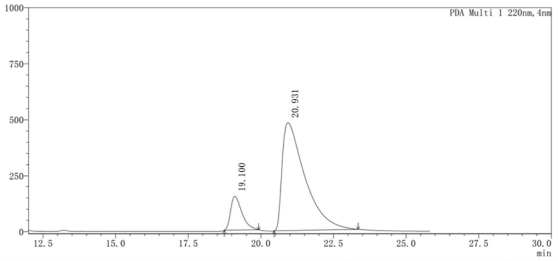

Entry	Retention time	Peak width (height 50%)	Height	Area	Area%
1	17.922	0.602	372506	15395989	48.368
2	20.325	1.130	193606	15790358	50.632

Entry	Retention time	Peak width (height 50%)	Height	Area	Area%
1	19.100	0.409	150499	3362619	10.261
2	20.931	0.807	482654	29408500	89.739

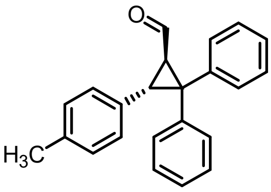

5b

5b: Yield 90%.

The enantiomeric purity of **5b** was determined by HPLC analysis in comparison with racemic product (Chiralpak ID, 100:1 hexane: *i*-PrOH, 1.0 mL/min, 220 nm): t_{minor} = 18.6 min, t_{major} = 20.6 min.

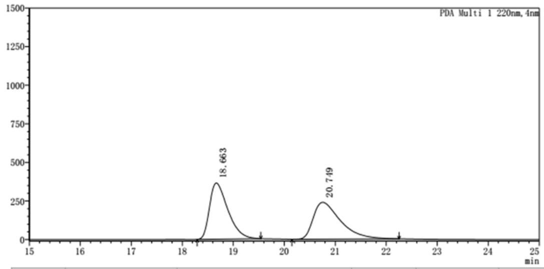

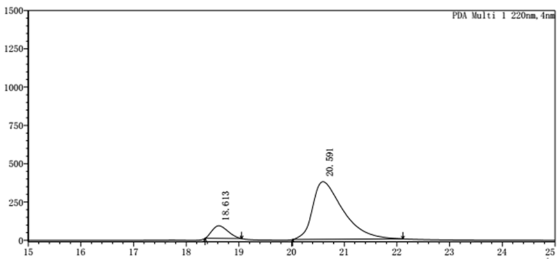

Entry	Retention time	Peak width (height 50%)	Height	Area	Area%
1	18.663	0.383	364105	9106857	50.724
2	20.749	0.554	238775	8846942	49.276

Entry	Retention time	Peak width (height 50%)	Height	Area	Area%
1	18.613	0.342	81983	1721994	10.214
2	20.591	0.596	376005	15137319	89.786

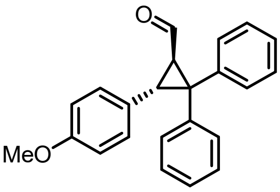

5c

5c: Yield 95%.

The enantiomeric purity of **5c** was determined by HPLC analysis in comparison with racemic product (Chiralpak ID, 100:1 hexane: *i*-PrOH, 1.0 mL/min, 220 nm): t_{minor} = 36.6 min, t_{major} = 41.8 min.

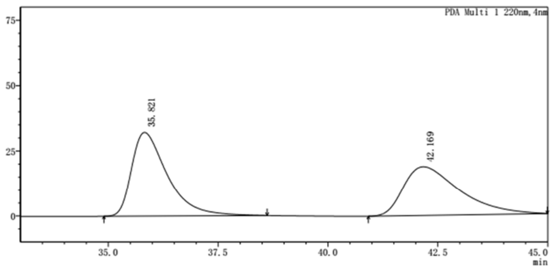

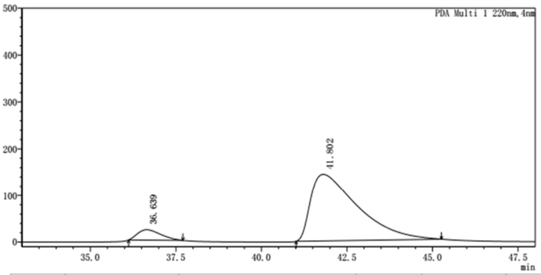

Entry	Retention time	Peak width (height 50%)	Height	Area	Area%
1	35.821	0.837	32156	1830215	52.268
2	42.169	1.346	18766	1671405	47.732

Entry	Retention time	Peak width (height 50%)	Height	Area	Area%
1	36.639	0.764	22093	1043019	7.090
2	41.802	1.447	143091	13668151	92.910

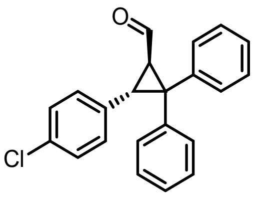

5e

3-(4-Chlorophenyl)-2,2-diphenylcyclopropane-1-carbaldehyde (**5e**): Yield 92%. **^{1}H NMR** (400 MHz, $CDCl_3$) δ 8.90 (d, *J* = 6.3 Hz, 1 H), 7.47 (d, *J* = 7.5 Hz, 2 H), 7.34–7.31 (m, 2 H), 7.25–7.20 (m, 1 H), 7.18–7.05 (m, 7 H), 6.86 (d, *J* = 8.3 Hz, 2 H), 3.71 (d, *J* = 5.6 Hz, 1 H), 3.10–3.07 (m, 1 H). **^{13}C NMR** (101 MHz, $CDCl_3$) δ 199.4, 141.0, 139.2, 133.7, 132.6, 129.4, 129.3, 129.3, 129.1, 128.6, 128.3, 127.5, 127.1, 48.7, 41.0, 34.8. **HRMS (ESI)** calcd for $C_{22}H_{17}ClNaO^+$ (M + Na$^+$) 355.0860, found 355.0846.

The enantiomeric purity of **5e** was determined by HPLC analysis in comparison with racemic product (Chiralpak ID, 100:1 hexane:*i*-PrOH, 1.0 mL/min, 220 nm): t_{minor} = 19.7min, t_{major} = 22.9 min.

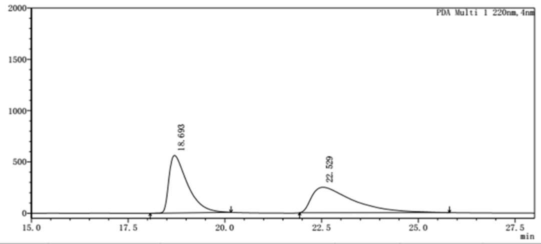

Entry	Retention time	Peak width (height 50%)	Height	Area	Area%
1	18.693	0.497	560676	18733568	50.287
2	22.529	1.091	247669	18519757	48.713

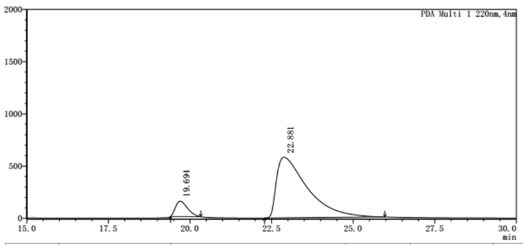

Entry	Retention time	Peak width (height 50%)	Height	Area	Area%
1	19.694	0.409	147581	3787947	8.941
2	22.881	0.970	580647	38579673	91.059

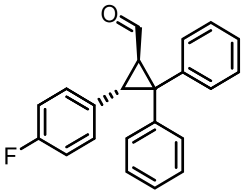

5f

3-(4-Fluorophenyl)-2,2-diphenylcyclopropane-1-carbaldehyde (**5f**): Yield 88%. **^{1}H NMR** (400 MHz, $CDCl_3$) δ 8.93 (d, *J* = 6.4 Hz, 1 H), 7.52 (d, *J* = 7.1 Hz, 2 H), 7.38–7.34 (m, 2 H), 7.27 (d, *J* = 8.0 Hz, 1 H), 7.14 (m, 5 H), 6.97–6.91 (m, 2 H), 6.87 (t, *J* = 8.7 Hz, 2 H), 3.77 (d, *J* = 5.7 Hz, 1 H), 3.14–3.11 (m, 1H). **^{13}C NMR** (101 MHz, $CDCl_3$) δ 199.6, 162.7 (d, *J* = 246 Hz), 141.1, 139.4, 130.8 (d, *J* = 3.1 Hz), 129.6 (d, *J* = 8.1 Hz), 129.4, 129.4, 129.1, 128.5, 127.5, 127.0, 115.0 (d, *J* = 22.2 Hz), 48.5, 41.0, 34.7. **HRMS (ESI)** calcd for $C_{22}H_{18}FO^+$ (M + H$^+$) 317.1336, found 317.1326.

The enantiomeric purity of **5f** was determined by HPLC analysis in comparison with racemic product (Chiralpak ID, 100:1 hexane:*i*-PrOH, 1.0 mL/min, 220 nm): t_{minor} = 18.6 min, t_{major} = 20.7 min.

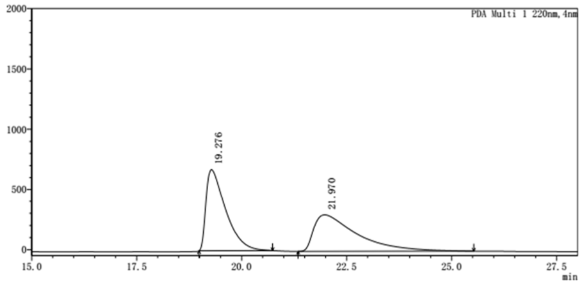

Entry	Retention time	Peak width (height 50%)	Height	Area	Area%
1	19.276	0.487	674351	21850448	50.210
2	21.970	1.030	301828	21667625	49.790

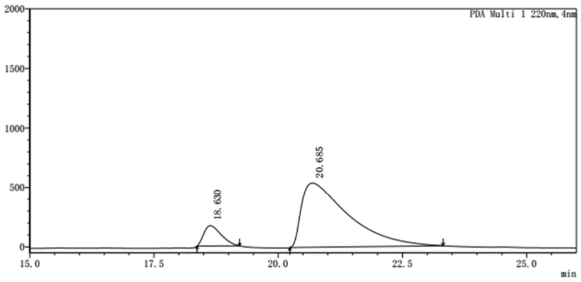

Entry	Retention time	Peak width (height 50%)	Height	Area	Area%
1	18.630	0.391	169630	4141960	10.442
2	20.685	0.969	542208	35524390	89.558

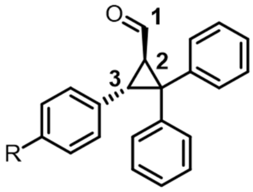

^{1}H NMR	H_1	H_2	H_3
**5a**	8.90	3.15-3.12	3.76
**5b**	8.87	3.10-3.07	3.72
**5c**	8.86	3.07-3.04	3.71
**5d**	8.99	3.27–3.24	3.85
**5e**	8.90	3.10-3.07	3.71
**5f**	8.93	3.14-3.11	3.77

Figure S2. The summary of assignments of partial ^{1}H NMR signals for the aldehydes **5**.

6. Reactions of Iminium Hexafluorophosphates 1 with Aryldiazomethanes 6

General Procedure: Solutions of the aryldiazomethanes **6** (0.50 mmol in 2 mL of CH_2Cl_2) were slowly added to solutions of the iminium hexafluorophosphates **1a**, **1b**, or **1d** (0.20 mmol in 2 mL of CH_2Cl_2) at –40 °C and stirred while the color of the aryldiazomethane slowly faded. After the solution became pale yellow (about 30 min), the temperature was raised to room temperature and the aq phosphate buffer solution (5 mL) was added. After stirring for 30 min, the mixture was extracted with CH_2Cl_2 (3 × 3 mL) and dried over $MgSO_4$. After evaporation of the solvent under reduced pressure, the product was purified by column chromatography (silica gel, EtOAc/hexane 10/100 v/v).

Reaction of iminium hexafluorophosphate 1a with phenyldiazomethane 6a

Following General Procedure, **1a** (96 mg, 0.20 mmol) was combined with **6a** (59 mg, 0.50 mmol) to give **7a** (45 mg, 72%) and **8** (19%).

Under the conditions of the ESI method, the aldehydes **7a** and **8** oxidized to the corresponding carboxylates [that is, (M) for the aldehydes converted to ($M[O]^-$) for the carboxylates] that were detected in the HRMS.

Molecular Weight: 312.4120

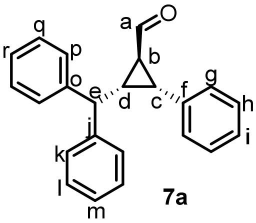

7a

2-Benzhydryl-3-phenylcyclopropane-1-carbaldehyde (**7a**): Yield 72%. **^{1}H NMR** (599 MHz, $CDCl_3$) δ 9.57 (d, J = 3.9 Hz, 1 H, H^a), 7.29–7.24 (m, 5 H, $H^{g,i,l}$), 7.22 – 7.11 (m, 6 H, $H^{k,m,p,r}$), 7.10–7.05 (m, 2 H, H^h), 6.90–6.86 (m, 2 H, H^q), 3.40 (d, J = 11.0 Hz, 1 H, H^e), 3.15 (dd, J = 9.5, 5.1 Hz, 1 H, H^c), 2.64 (ddd, J = 11.0, 9.5, 4.8 Hz, 1 H, H^d), 2.51 (ddd, J = 5.0, 5.0, 3.9 Hz, 1 H, H^b). **^{13}C NMR** (151 MHz, $CDCl_3$) δ 200.0 (C^a), 144.1 (C^o), 143.4 (C^j), 134.7 (C^f), 129.1 (2 C, C^h), 128.8 (2 C, C^l), 128.4 (4 C, $C^{g,p}$), 128.0 (2 C, C^q), 128.0 (2 C, C^k), 127.2 (C^i), 126.7 (C^r), 126.5 (C^m), 48.3 (C^e), 35.0 (C^d), 34.8 (C^b), 33.5 (C^c). **HRMS (ESI)** calcd for $C_{23}H_{19}O_2^-$ ($M[O]^-$): calcd. 327.1391, found 327.1392.

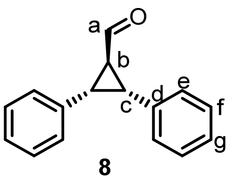

8

2,3-Diphenylcyclopropane-1-carbaldehyde (**8**): Yield 19%. **^{1}H NMR** (599 MHz, $CDCl_3$) δ 9.65 (d, J = 4.1 Hz, 1 H, H^a), 7.22–7.11 (m, 6 H, $H^{e,g}$), 6.98–6.93 (m, 4 H, H^f), 3.19 (d, J = 5.2 Hz, 2 H, H^c), 2.84 (td, J = 5.2, 4.1 Hz, 1 H, H^b). **^{13}C NMR** (151 MHz, $CDCl_3$) δ 199.8 (C^a), 135.0 (C^d), 129.1 (C^f), 128.2 (C^e), 126.9 (C^g), 37.0 (C^b), 33.6 (C^c). **HRMS (ESI)** calcd for $C_{16}H_{13}O_2^-$ ($M[O]^-$) 237.0921, found 237.0923.

NMR spectroscopic data are in agreement with those in the literature.[S3]

Reaction of iminium hexafluorophosphate 1a with (4-cyanophenyl)diazomethane 6b

Following General Procedure, **1a** (96 mg, 0.20 mmol) was combined with **6b** (72 mg, 0.50 mmol) to give **7b** (54 mg, 74%) as a mixture of two diastereoisomers, dr = 81:19.

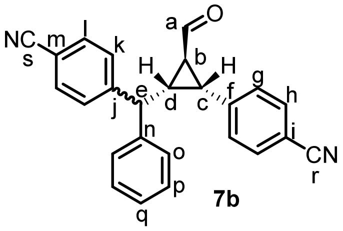

7b

4-((2-((4-Cyanophenyl)(phenyl)methyl)-3-formylcyclopropyl)benzonitrile (**7b**): Yield 76%. *-signals of major isomer. #-signals of minor isomer.

^{1}H NMR (599 MHz, $CDCl_3$) δ 9.68 (d, *J* = 3.0 Hz, 1 H, H^{a}),# 9.65 (d, *J* = 3.3 Hz, 1 H, H^{a}),* 7.57 (d, *J* = 8.3 Hz, 2 H, H^{l}),* 7.57 (d, *J* = 8.3 Hz, 2 H, H^{h}),# 7.53 (d, *J* = 8.3 Hz, 2 H, H^{h}),* 7.48 (d, *J* = 8.3 Hz, 2 H, H^{l}),# 7.31–7.29 (m, 2 H, H^{o}),# 7.27–7.24 (m, 1 H, H^{q}),# 7.25 (d, *J* = 6.9 Hz, 2 H, H^{k}),* 7.23–7.19 (m, 3 H, H^{o,q}),* 7.16 (d, *J* = 8.3 Hz, 2 H, H^{g}),# 7.14 (d, *J* = 8.3 Hz, 2 H, H^{g}),* 7.10 (d, *J* = 7.3 Hz, 2 H, H^{p}),# 6.98 (d, *J* = 8.4 Hz, 2 H, H^{k}),# 6.88–6.81 (m, 2 H, H^{p}),* 3.34 (d, *J* = 11.0 Hz, 1 H, H^{e}),# 3.29 (d, *J* = 11.1 Hz, 1 H, H^{e}),* 3.16 (dd, *J* = 9.6, 5.1 Hz, 1 H, H^{c}),*,# 2.69 (ddd, *J* = 11.0, 9.6, 4.8 Hz, 1 H, H^{d}),* 2.72–2.65 (m, 1 H, H^{d}),# 2.63 (ddd, *J* = 5.1, 5.1, 3.0 Hz, 1 H, H^{b}),# 2.58 (ddd, *J* = 5.0, 5.0, 3.3 Hz, 1 H, H^{b})*. **^{13}C NMR** (151 MHz, $CDCl_3$) δ 198.6 (C^{a}),# 198.5 (C^{a}),* 148.8 (C^{j}),* 148.0 (C^{j}),# 142.0 (C^{n}),# 141.0 (C^{n}),* 140.2 (C^{f}),# 140.0 (C^{f}),* 132.8 (2 C, C^{l}),* 132.4 (2 C, C^{l}),# 132.3 (2 C, C^{h}),# 132.1 (2 C, C^{h}),* 129.8 (2 C, C^{g}),* 129.5 (2 C, C^{g}),# 129.2 (2 C, C^{o}),# 128.9 (2 C, C^{o}),* 128.5 (2 C, C^{k}),* 128.5 (2 C, C^{k}),# 127.73 (2 C, C^{p}),* 127.65 (2 C, C^{p}),# 127.54 (C^{q}),# 127.47 (C^{q}),* 118.7 (C^{r}),* 118.6 (C^{s}),* 118.6 (C^{s}),# 118.5 (C^{r}),# 111.5 (C^{i}),# 111.4 (C^{i}),* 111.02 (C^{m}),* 110.95 (C^{m}),# 49.0 (C^{e}),* 48.6 (C^{e}),# 34.7 (C^{d}),# 34.4 (C^{b}),* 34.3 (C^{d}),* 34.2 (C^{b}),# 33.1 (C^{c}),# 32.9 (C^{c}).* **HRMS (ESI)** calcd for $C_{25}H_{17}N_2O^-$ (M – H^+) 361.1346, found 361.1352.

Reaction of iminium hexafluorophosphate 1b with (4-cyanophenyl)diazomethane 6b

Following General Procedure, **1b** (98 mg, 0.20 mmol) was combined with **3b** (72 mg, 0.50 mmol) to give **7c** (53 mg, 70%) as a mixture of two diastereoisomers, dr = 79:21.

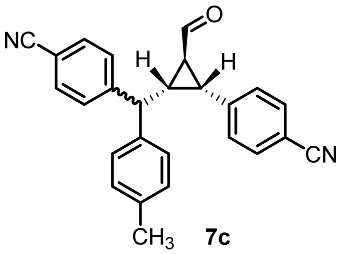

7c

4-(2-((4-Cyanophenyl)(*p*-tolyl)methyl)-3-formylcyclopropyl)benzonitrile (**7c**): Yield 70%. Major isomer: **^{1}H NMR** (400 MHz, $CDCl_3$) δ 9.66 (d, *J* = 3.3 Hz, 1 H), 7.59–7.55 (m, 4 H), 7.27 (d, *J* = 8.2 Hz, 2 H), 7.18 (d, *J* = 8.2 Hz, 2 H), 7.04 (d, *J* = 7.9 Hz, 2 H), 6.75 (d, *J* = 8.0 Hz, 2 H), 3.27 (d, *J* = 11.1 Hz, 1 H), 3.18 (dd, *J* = 9.5, 5.1 Hz, 1 H), 2.75–2.66 (m, 1 H), 2.57 (dd, *J* = 8.4, 5.0 Hz, 1 H), 2.32 (s, 3 H). **HRMS (ESI)** calcd for $C_{26}H_{20}N_2NaO^+$ (M + Na^+) 399.1468, found 399.1458.

Reaction of iminium hexafluorophosphate 1d with (4-cyanophenyl)diazomethane 6b

Following General Procedure, **1d** (105 mg, 0.20 mmol) was combined with **6b** (72 mg, 0.50 mmol) to give **7d** (57 mg, 0.14 mmol, 70%) as a mixture of two diastereoisomers, dr = 83:17.

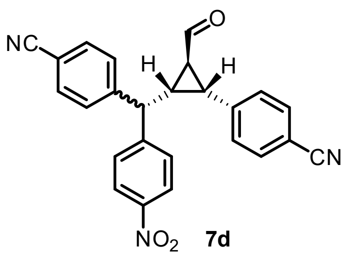

7d

4-(2-((4-Cyanophenyl)(4-nitrophenyl)methyl)-3-formylcyclopropyl)benzonitrile (**7d**): Yield 70%. Major isomer: **^{1}H NMR** (400 MHz, $CDCl_3$) δ 9.73 (d, J = 2.1 Hz, 1 H), 8.08 (d, J = 8.5 Hz, 2 H), 7.63–7.57 (m, 4 H), 7.26–7.24 (m, 2 H), 7.17 (d, J = 7.9 Hz, 2 H), 7.03 (d, J = 8.4 Hz, 2 H), 3.44 (d, J = 10.9 Hz, 1 H), 3.19 (dd, J = 9.2, 5.2 Hz, 1 H), 2.78–2.61 (m, 2 H). **HRMS (ESI)** calcd for $C_{25}H_{17}N_3NaO_3^+$ (M + Na^+) 430.1162, found 430.1154.

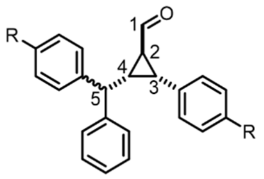

^{1}H NMR	H_1	H_2	H_3	H_4	H_5
**7a**	9.57	2.51	3.15	2.64	3.40
**7b**	9.68	2.58	3.16	2.69	3.34
**7c**	9.66	2.57	3.18	2.75-2.66	3.27
**7d**	9.73	2.78-2.61	3.19	2.78-2.61	3.44

Figure S3. The summary of assignments of partial ^{1}H NMR signals for the cyclopropane-1-carbaldehydes **7**.

7. Oxidation of 7b

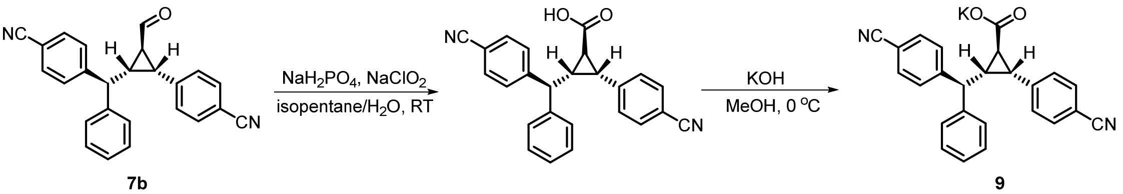

A solution of NaH_2PO_4 (71 mg) and $NaClO_2$ (69 mg) in water (1.0 mL) was added dropwise to a solution of **7b** (29 mg) in isopentane (0.50 mL). The mixture was stirred at room temperature overnight and then poured into brine. The crude mixture was extracted with ethyl acetate and the organic layers were combined and dried over Na_2SO_4. The crude product was purified by flash chromatography (ethyl acetate:pentane = 2:1) and dissolved in 1 mL of MeOH. After addition of 46 µL of a methanolic solution of KOH (1.74 M) at 0 °C the mixture was kept at room temperature for 1 h. After evaporation of the solvent an oily mixture remained which was dissolved in EtOH/THF to yield crystals of **9** suitable for single crystal XRD analysis.

8. Attempts to Catalyze the Reactions of Cinnamaldehyde with Aryldiazomethanes by MacMillan Catalysts

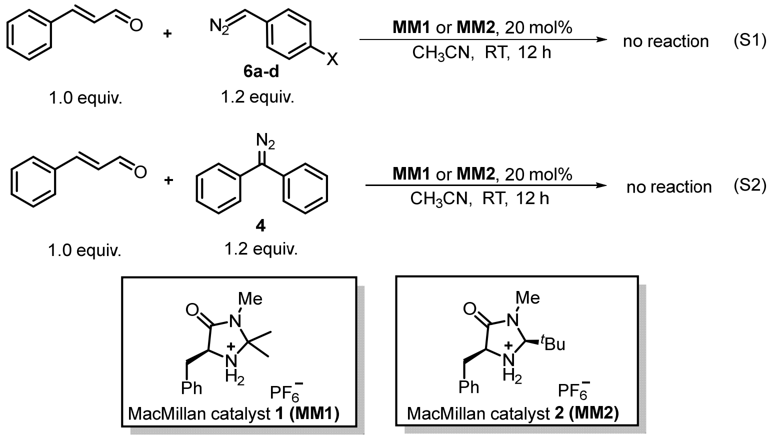

To a solution of cinnamaldehyde (0.20 mmol) and MacMillan catalyst (**MM1** or **MM2**, 0.04 mmol, 20 mol%) in CH_3CN (2.0 mL), aryldiazomethanes **6a-d** or **4** (1.2 equiv) were added at ambient temperature and stirred for 12 h. TLC analysis showed mostly non-converted starting materials and the absence of products **5**, **7**, and **8**.

In order to explore the reason for the failure of these reactions, the aryldiazomethanes were combined with an equimolar amount of the imidazolidinone hexafluorophosphates **MM1** and **MM2.** Immediate disappearance of the color of the aryldiazomethanes and evolution of gas was observed indicating that the aryldiazomethanes deprotonate and thus deactivate the imidazolidinone hexafluorophosphates **MM1** and **MM2.**

9. Kinetics of the Reactions of Iminium Salts with Aryldiazomethanes

Table S1. Kinetics of the reaction of **1a** with **6a** in CH_2Cl_2 at 20 °C (stopped-flow, λ = 370 nm).

**[1a]**/M	**[6a]**/M	**[6a]**/**[1a]**	k_{obs}/s^{-1}
3.76×10^{-5}	6.52×10^{-4}	17.3	1.17
3.76×10^{-5}	9.77×10^{-4}	26.0	1.82
3.76×10^{-5}	1.30×10^{-3}	34.6	2.56
3.76×10^{-5}	1.63×10^{-3}	43.3	3.16
$k_2 = 2.07 \times 10^3$ M^{-1} s^{-1}			

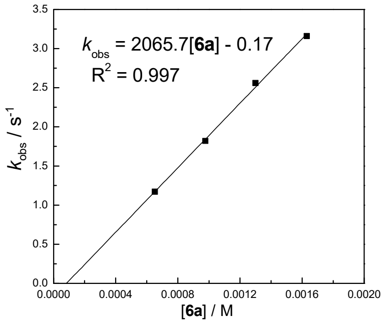

Table S2. Kinetics of the reaction of **1a** with **6b** in CH_2Cl_2 at 20 °C (J&M, λ = 370 nm).

**[1a]**/M	**[6b]**/M	**[1a]**/**[6b]**	k_{obs}/s^{-1}
8.77×10^{-5}	1.17×10^{-3}	13.3	2.57×10^{-2}
8.73×10^{-5}	1.55×10^{-3}	17.8	3.73×10^{-2}
8.70×10^{-5}	1.93×10^{-3}	22.2	4.58×10^{-2}
8.66×10^{-5}	2.31×10^{-3}	26.6	5.69×10^{-2}
$k_2 = 2.69 \times 10^1$ M^{-1} s^{-1}			

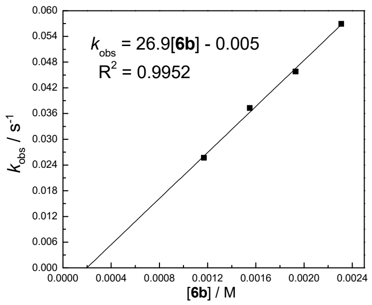

Table S3. Kinetics of the reaction of **1a** with **6c** in CH_2Cl_2 at 20 °C (stopped-flow, λ = 370 nm).

**[1a]**/M	**[6c]**/M	**[1a]**/**[6c]**	k_{obs}/s^{-1}
1.48×10^{-4}	1.54×10^{-3}	10.4	6.90×10^{-1}
1.48×10^{-4}	2.05×10^{-3}	13.9	9.31×10^{-1}
1.48×10^{-4}	2.57×10^{-3}	17.3	1.20
1.48×10^{-4}	3.08×10^{-3}	20.8	1.44
1.48×10^{-4}	3.60×10^{-3}	24.3	1.61
$k_2 = 4.56 \times 10^2$ M^{-1} s^{-1}			

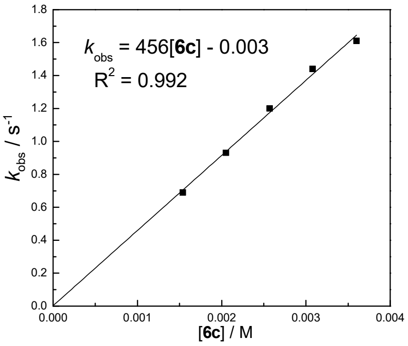

Table S4. Kinetics of the reaction of **1b** with **6a** in CH_2Cl_2 at 20 °C (stopped-flow, λ = 388 nm).

**[1b]**/M	**[6a]**/M	**[6a]**/**[1b]**	k_{obs}/s^{-1}
2.03×10^{-5}	6.52×10^{-4}	32.1	3.54×10^{-1}
2.03×10^{-5}	9.77×10^{-4}	48.1	5.38×10^{-1}
2.03×10^{-5}	1.30×10^{-3}	64.2	7.35×10^{-1}
2.03×10^{-5}	1.63×10^{-3}	80.2	9.44×10^{-1}
2.03×10^{-5}	1.95×10^{-3}	96.3	1.15
$k_2 = 6.11 \times 10^2$ M^{-1} s^{-1}			

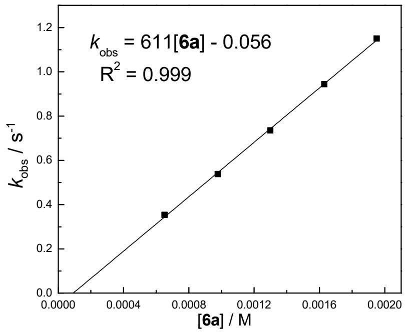

Table S5. Kinetics of the reaction of **1b** with **6b** in CH_2Cl_2 at 20 °C (J&M, λ = 388 nm).

[**1b**]/M	[**6b**]/M	[**6b**]/[**1b**]	k_{obs}/s^{-1}
1.11×10^{-4}	1.51×10^{-3}	13.6	1.93×10^{-2}
1.11×10^{-4}	1.88×10^{-3}	17.0	2.30×10^{-2}
1.10×10^{-4}	2.25×10^{-3}	20.4	3.04×10^{-2}
1.10×10^{-4}	2.61×10^{-3}	23.9	3.64×10^{-2}
1.09×10^{-4}	2.98×10^{-3}	27.3	4.04×10^{-2}
$k_2 = 1.51 \times 10^{1}$ M^{-1} s^{-1}			

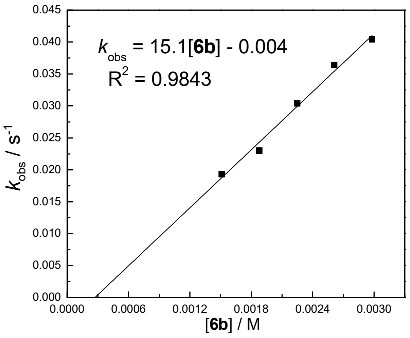

Table S6. Kinetics of the reaction of **1b** with **6c** in CH_2Cl_2 at 20 °C (stopped-flow, λ = 388 nm).

[**1b**]/M	[**6c**]/M	[**6c**]/[**1b**]	k_{obs}/s^{-1}
3.14×10^{-5}	1.07×10^{-3}	33.9	2.27×10^{-1}
3.14×10^{-5}	1.60×10^{-3}	50.9	3.60×10^{-1}
3.14×10^{-5}	2.13×10^{-3}	67.8	4.74×10^{-1}
3.14×10^{-5}	2.66×10^{-3}	84.8	5.47×10^{-1}
3.14×10^{-5}	3.20×10^{-3}	108	6.60×10^{-1}
$k_2 = 1.98 \times 10^{2}$ M^{-1} s^{-1}			

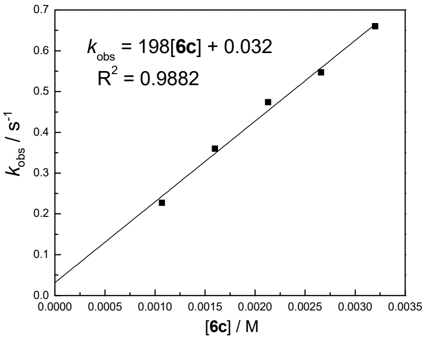

Table S7. Kinetics of the reaction of **1c** with **6a** in CH_2Cl_2 at 20 °C (stopped-flow, λ = 426 nm).

[**1c**]/M	[**6a**]/M	[**6a**]/[**1c**]	k_{obs}/s^{-1}
1.38×10^{-5}	6.52×10^{-4}	47.3	8.35×10^{-2}
1.38×10^{-5}	9.77×10^{-4}	71.0	1.26×10^{-1}
1.38×10^{-5}	1.30×10^{-3}	94.7	1.67×10^{-1}
1.38×10^{-5}	1.63×10^{-3}	118	2.13×10^{-1}
1.38×10^{-5}	1.95×10^{-3}	142	2.59×10^{-1}
$k_2 = 1.35 \times 10^{2}$ M^{-1} s^{-1}			

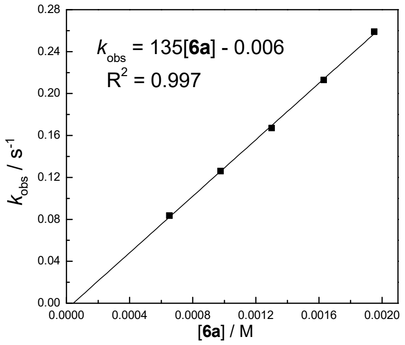

Table S8. Kinetics of the reaction of **1c** with **6b** in CH_2Cl_2 at 20 °C (J&M, λ = 426 nm).

[**1c**]/M	[**6b**]/M	[**6b**]/[**1c**]	k_{obs}/s^{-1}
3.39×10^{-5}	1.49×10^{-3}	43.9	3.63×10^{-3}
3.38×10^{-5}	1.85×10^{-3}	54.9	4.71×10^{-3}
3.36×10^{-5}	2.21×10^{-3}	65.9	5.50×10^{-3}
3.35×10^{-5}	2.57×10^{-3}	76.9	6.61×10^{-3}
3.33×10^{-5}	2.93×10^{-3}	87.8	7.97×10^{-3}
$k_2 = 2.94$ M^{-1} s^{-1}			

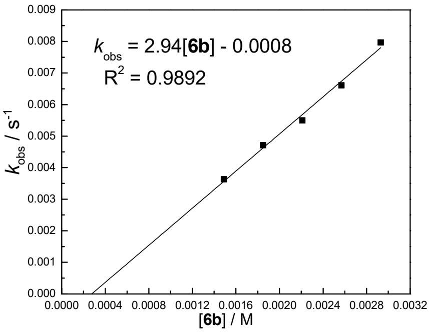

Table S9. Kinetics of the reaction of **1c** with **6c** in CH_2Cl_2 at 20 °C (stopped-flow, λ = 426 nm).

[**1c**]/M	[**6c**]/M	[**6c**]/[**1c**]	k_{obs}/s^{-1}
4.78×10^{-5}	2.03×10^{-3}	42.5	8.14×10^{-2}
4.78×10^{-5}	2.54×10^{-3}	53.1	1.06×10^{-1}
4.78×10^{-5}	3.05×10^{-3}	63.7	1.30×10^{-1}
4.78×10^{-5}	3.55×10^{-3}	74.3	1.47×10^{-1}
4.78×10^{-5}	4.06×10^{-3}	85.0	1.80×10^{-1}
$k_2 = 4.70 \times 10^{1}$ M^{-1} s^{-1}			

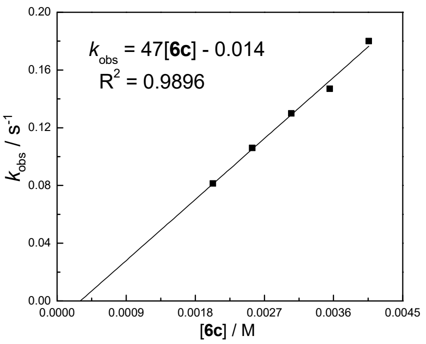

Table S10. Kinetics of the reaction of **1a** with **4** in CH_2Cl_2 at 20 °C (UV-Vis, λ = 400 nm).

[**1a**]/M	[**4**]/M	[**4**]/[**1a**]	k_{obs}/s^{-1}
9.48×10^{-5}	4.26×10^{-3}	44.9	4.91×10^{-4}
9.48×10^{-5}	4.38×10^{-3}	46.2	5.08×10^{-4}
9.48×10^{-5}	5.10×10^{-3}	53.8	5.91×10^{-4}
9.48×10^{-5}	5.79×10^{-3}	61.1	7.23×10^{-4}
$k_2 = 1.48 \times 10^{-1}$ M^{-1} s^{-1}			

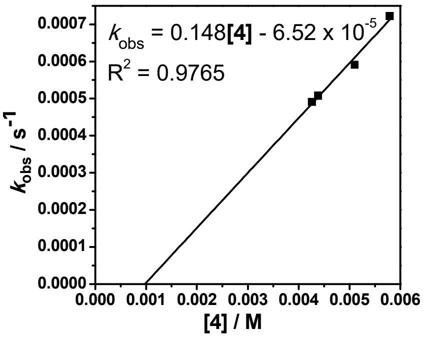

Table S11. Kinetics of the reaction of **1b** with **4** in CH_2Cl_2 at 20 °C (UV-Vis, λ = 410 nm).

[**1b**]/M	[**4**]/M	[**4**]/[**1b**]	k_{obs}/s^{-1}
3.25×10^{-5}	2.18×10^{-3}	67.1	1.36×10^{-4}
3.25×10^{-5}	2.70×10^{-3}	83.1	1.63×10^{-4}
3.25×10^{-5}	3.30×10^{-3}	102	2.12×10^{-4}
3.25×10^{-5}	3.87×10^{-3}	119	2.42×10^{-4}
$k_2 = 6.48 \times 10^{-2}$ M^{-1} s^{-1}			

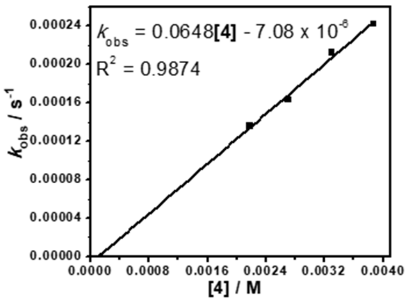

Table S12. Kinetics of the reaction of **1c** with **4** in CH_2Cl_2 at 20 °C (UV-Vis, λ = 425 nm).

[**1c**]/M	[**4**]/M	[**4**]/[**1c**]	k_{obs}/s^{-1}
1.14×10^{-5}	2.65×10^{-3}	23.2	4.95×10^{-5}
1.14×10^{-5}	3.17×10^{-3}	27.8	5.74×10^{-5}
1.14×10^{-5}	3.53×10^{-3}	31.0	6.33×10^{-5}
1.14×10^{-5}	4.06×10^{-3}	35.6	7.44×10^{-5}
$k_2 = 1.76 \times 10^{-2}$ M^{-1} s^{-1}			

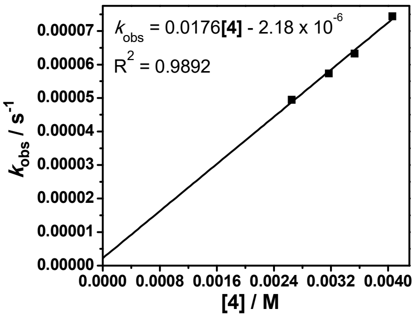

Table S13. Kinetics of the reaction of **1d** with **4** in CH_2Cl_2 at 20 °C (UV-Vis, λ = 390 nm).

**[1d]**/M	**[4]**/M	**[4]**/**[1d]**	k_{obs}/s^{-1}
7.79×10^{-5}	1.35×10^{-3}	17.3	1.03×10^{-3}
7.79×10^{-5}	1.58×10^{-3}	20.3	1.14×10^{-3}
7.79×10^{-5}	1.71×10^{-3}	22.0	1.21×10^{-3}
7.79×10^{-5}	1.96×10^{-3}	25.2	1.32×10^{-3}
$k_2 = 4.73 \times 10^{-1}$ M^{-1} s^{-1}			

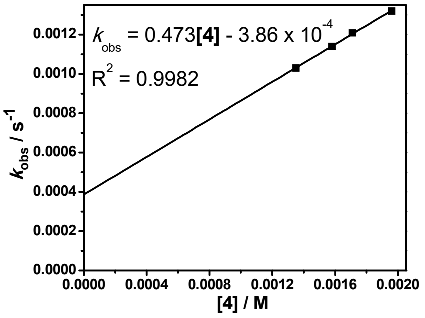

Table S14. Kinetics of the reaction of **2a** with **4** in CH_2Cl_2 at 20 °C (UV-Vis, λ = 400 nm).

**[2a]**/M	**[4]**/M	**[4]**/**[2a]**	k_{obs}/s^{-1}
2.87×10^{-5}	1.07×10^{-3}	37.3	6.45×10^{-4}
2.87×10^{-5}	1.99×10^{-3}	69.3	1.12×10^{-3}
2.87×10^{-5}	2.78×10^{-3}	96.9	1.56×10^{-3}
2.87×10^{-5}	3.53×10^{-3}	123	2.01×10^{-3}
$k_2 = 5.54\times 10^{-1}$ M^{-1} s^{-1}			

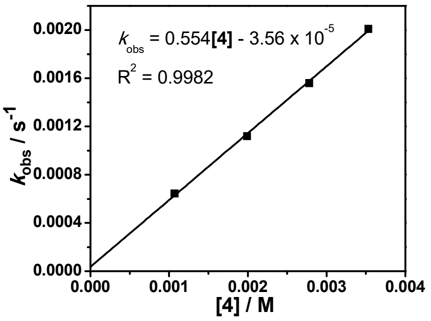

10. Computational Details

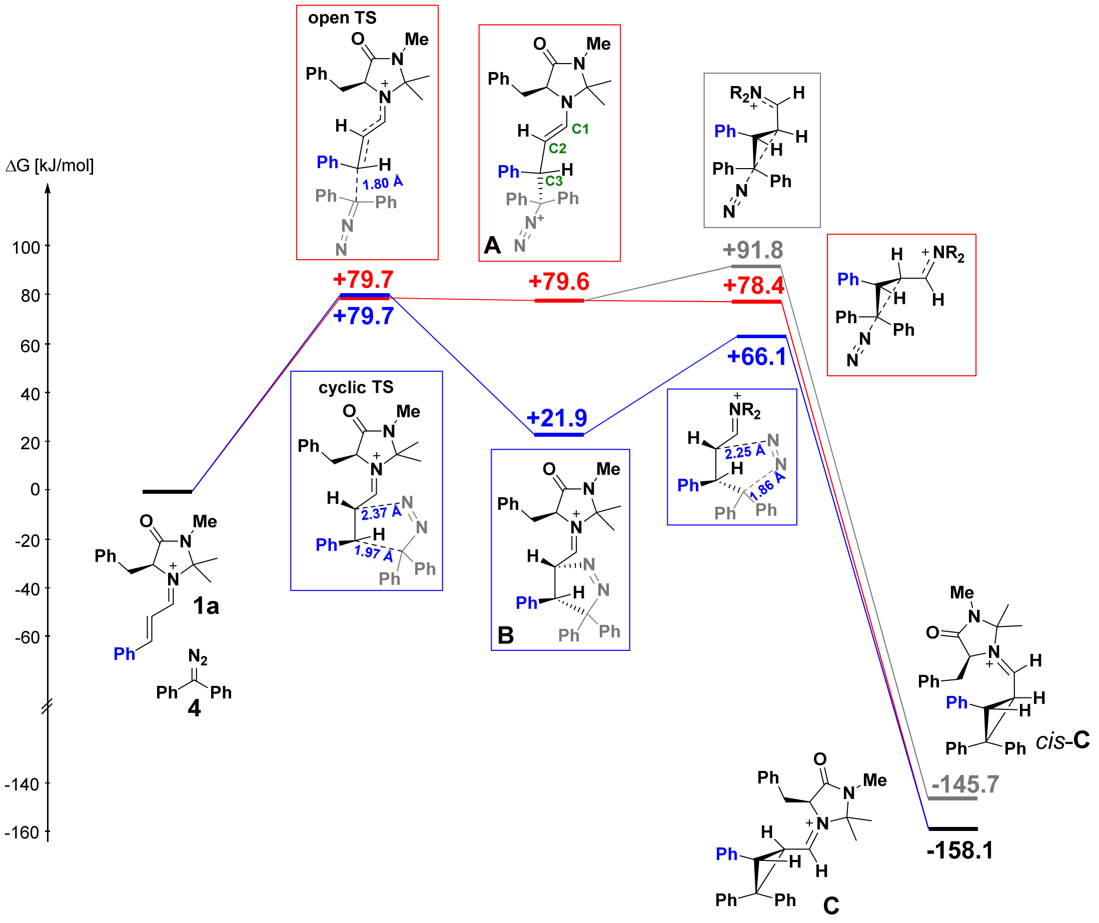

Figure S4. Gibbs energy profile for the reaction of iminium ion **1a** with diphenyldiazomethane (**4**) at the (SMD=DCM)//B3LYP-D3BJ/def2svp level of theory, which additionally shows the transition state for the formation of *cis*-configured **C** (in grey) that was not observed experimentally.

Initially, all structures were subjected to a conformational search with the OPLS3[S4] force field as implemented in MacroModel[S5] with the MCMM method in gas phase. All subsequent quantum-chemical calculations were performed with the Gaussian 16 A.03 set of codes.[S6] All structures were optimized at the B3LYP-D3BJ/def2svp[S7] level of theory considering solvation by the SMD model for dichloromethane[S8] and dispersion corrections by the method of Grimme.[S9] Thermal corrections at 298 K were obtained at the same level of theory from vibrational frequencies and are unscaled. Transition states were validated by the presence of one imaginary frequency and IRC calculations. All conformers were Boltzmann weighted. Finally, a free energy correction +7.91 kJ mol^{-1} was applied to all free energies to consider the conversion from gas phase (1 atm) to liquid phase (1 M).

Table S15.

1a + 4				
	Filename	E_{tot}	G_{298}	Weighting
**1a**	REDO_1a_1	-1037.381312	-1037.007311	0.42631
	REDO_1a_2	-1037.382784	-1037.007301	0.42156
	REDO_1a_4	-1037.376233	-1037.006339	0.15213
	Weighted		-1037.007159	
**4a**	REDO_4_1	-610.487969	-610.331291	
Nitrogen	nitrogen	-109.434977	-109.447736	
cyclic TS (**1a** + **4a**)	REDO_f_7_ts1_3	-1647.862340	-1647.305094	0.50105
	REDO_f_7_ts1_4	-1647.862340	-1647.305090	0.49895
	Weighted		-1647.305092	
open TS (**1a** + **4a**)	REDO_f_7_ts1	-1647.861751	-1647.305283	0.93263
	REDO_f_17_ts1	-1647.858594	-1647.302702	0.06042
	REDO_f_7_ts1_rts1	-1647.855823	-1647.300662	0.00695
	Weighted		-1647.305095	
**A**	REDO_f_1	-1647.858990	-1647.304095	0.13647
	REDO_f_2	-1647.855015	-1647.299492	0.00104
	REDO_f_7	-1647.861878	-1647.305629	0.69409
	REDO_f_8	-1647.848235	-1647.293747	0.00000
	REDO_f_9	-1647.856407	-1647.299075	0.00067
	REDO_f_17	-1647.856922	-1647.301943	0.01394
	REDO_f_18	-1647.853827	-1647.298735	0.00046
	REDO_f_19	-1647.853227	-1647.297666	0.00015
	REDO_f_22	-1647.857732	-1647.304195	0.15168
	REDO_f_7_rot_4	-1647.860752	-1647.299837	0.00150
	Weighted		-1647.305127	
**B**	REDO_h_2	-1647.885454	-1647.322526	0.00673
	REDO_h_3	-1647.886739	-1647.323083	0.01216
	REDO_h_7	-1647.881870	-1647.318721	0.00012
	REDO_h_9	-1647.881089	-1647.318037	0.00006
	REDO_h_12	-1647.882520	-1647.321155	0.00157
	REDO_h_13	-1647.880806	-1647.319244	0.00021
	REDO_f_5	-1647.889663	-1647.327218	0.97456
	REDO_f_6	-1647.884739	-1647.322164	0.00459
	Weighted		-1647.327100	
TS (**A** to **C**)	f_7_cp_ts1	-1647.860941	-1647.305651	0.97847

	f_17_cp_ts1	-1647.856428	-1647.302052	0.02153
	Weighted		-1647.305574	
TS (**A** to *cis*-**C**)	REDO_f_17_rot_fr_ts1	-1647.854669	-1647.299849	0.28765
	REDO_f_7_rot_fr_ts1_2	-1647.860524	-1647.300704	0.71235
	Weighted		-1647.300458	
TS (**B** to **C**)	pes_h_2_190_230_ts1	-1647.865729	-1647.310562	0.88559
	REDO_h_3_fr_ts1	-1647.861544	-1647.304875	0.00213
	REDO_h_7_fr_ts1	-1647.861271	-1647.307534	0.03573
	REDO_f_5_fr_ts1	-1647.865910	-1647.308252	0.07654
	Weighted		-1647.310265	
**C**	REDO_trans_c_1	-1538.495054	-1537.942677	0.00135
	REDO_trans_c_2	-1538.500634	-1537.948147	0.44635
	REDO_trans_c_3	-1538.497242	-1537.946387	0.06905
	REDO_trans_c_6	-1538.495077	-1537.945425	0.02492
	REDO_trans_c_10	-1538.500634	-1537.948152	0.44873
	REDO_trans_c_11	-1538.494701	-1537.944524	0.00959
	Weighted		-1537.947917	
cis-**C**	REDO_g_1	-1538.486880	-1537.934050	0.00003
	REDO_g_3	-1538.496483	-1537.943634	0.78235
	REDO_g_4	-1538.484624	-1537.932861	0.00001
	REDO_g_5	-1538.494604	-1537.941277	0.06429
	REDO_g_6	-1538.484286	-1537.932877	0.00001
	REDO_g_7	-1538.492713	-1537.940099	0.01845
	REDO_g_8	-1538.494607	-1537.941976	0.13486
	Weighted		-1537.943193	

$\Delta G^{\ddagger}$ (cyclic TS)	79.7	kJ/mol
$\Delta G^{\ddagger}$ (open TS)	79.7	kJ/mol
$\Delta_r G^0$ (**A**)	79.6	kJ/mol
$\Delta_r G^0$ (**B**)	21.9	kJ/mol
$\Delta G^{\ddagger}$ (**A** to **C**)	78.4	kJ/mol
$\Delta G^{\ddagger}$ (**A** to *cis*-**C**)	91.8	kJ/mol
$\Delta G^{\ddagger}$ (**B** to **C**)	66.1	kJ/mol
$\Delta_r G^0$ (**C**)	-158.1	kJ/mol
$\Delta_r G^0$ (*cis*-**C**)	-145.7	kJ/mol

Table S16.

1a + 6a (Si attack)				
	Filename	E_{tot}	G_{298}	Weighting
**1a**	REDO_1a_1	-1037.381312	-1037.007311	0.42631
	REDO_1a_2	-1037.382784	-1037.007301	0.42156
	REDO_1a_4	-1037.376233	-1037.006339	0.15213
	Weighted		-1037.007159	
**6a**	REDO_3a_1	-379.564956	-379.482241	
Nitrogen	nitrogen	-109.434977	-109.447736	
cyclic TS (**1a** + **6a**, Si)	REDO_a_ss_7_ts1_rts_1	-1416.942942	-1416.464017	0.06749
	REDO_a_ss_7_ts1_rts_2	-1416.946195	-1416.466494	0.93251
	Weighted		-1416.466326	
open TS (**1a** + **6a**, Si)	REDO_a_ss_5_ts1	-1416.933741	-1416.454867	0.00002
	REDO_a_ss_7_ts1	-1416.943418	-1416.464910	0.99079
	REDO_REDO_a_ss_54_ts1	-1416.936190	-1416.457283	0.00030
	REDO_REDO_a_ss_56_ts1	-1416.934600	-1416.455893	0.00007
	REDO_REDO_a_ss_57_ts1	-1416.940983	-1416.460456	0.00881
	Weighted		-1416.464868	
**A''**	REDO_a_ss_1	-1416.946507	-1416.466094	0.01663
	REDO_a_ss_2	-1416.941796	-1416.463487	0.00105
	REDO_a_ss_3	-1416.941779	-1416.463348	0.00090
	REDO_a_ss_4	-1416.943406	-1416.463556	0.00113
	REDO_a_ss_6	-1416.940895	-1416.458561	0.00001
	REDO_a_ss_7	-1416.948239	-1416.469886	0.92616
	REDO_a_ss_8	-1416.945209	-1416.465370	0.00771
	REDO_a_ss_9	-1416.938977	-1416.461190	0.00009
	REDO_a_ss_11	-1416.939364	-1416.460517	0.00004
	REDO_a_ss_12	-1416.942668	-1416.462569	0.00040
	REDO_a_ss_14	-1416.946508	-1416.466079	0.01636
	REDO_a_ss_20_2	-1416.943753	-1416.465220	0.00658
	REDO_a_ss_27	-1416.943330	-1416.462887	0.00055
	REDO_a_ss_56	-1416.939506	-1416.461668	0.00015
	REDO_a_ss_57	-1416.945506	-1416.466368	0.02224
	Weighted		-1416.469588	
**B''**	d_sss_jj_prod	-1416.968736	-1416.473996	0.00006
	REDO_d_sss_2	-1416.967404	-1416.479118	0.01279
	REDO_d_sss_4_2	-1416.967104	-1416.480721	0.07001
	REDO_d_sss_5	-1416.966000	-1416.480559	0.05893

	REDO_d_sss_7	-1416.968657	-1416.483084	0.85768
	REDO_d_sss_8	-1416.963138	-1416.474981	0.00016
	REDO_d_sss_9	-1416.962810	-1416.475792	0.00038
	Weighted		-1416.482715	
TS (**A''** to *trans*-**C''**)	a_ss_12_cp_ts1	-1416.941836	-1416.464674	0.00354
	a_ss_20_cp_ts1	-1416.942141	-1416.465826	0.01199
	a_ss_7_cp_ts1	-1416.946475	-1416.469966	0.96578
	REDO_REDO_a_ss_54_frozen	-1416.944526	-1416.465438	0.00795
	REDO_REDO_a_ss_57_frozen	-1416.944199	-1416.465723	0.01075
	Weighted		-1416.469816	
TS (**B''** to *cis*-**C''**)	pes_d_endo_233_187_ts1	-1416.945469	-1416.464190	0.08269
	REDO_d_sss_4_n2_ts1	-1416.945108	-1416.466209	0.70310
	REDO_d_sss_5_n2_ts1	-1416.944246	-1416.464463	0.11048
	REDO_d_sss_9_n2_ts1	-1416.940728	-1416.464404	0.10373
	Weighted		-1416.465662	
TS (**B''** to **D**)	pes_d_endo_233_187_ts1_cshift_ts1	-1307.529251	-1307.056030	0.01714
	REDO_d_sss_4_n2_ts1_cshift_ts1	-1307.531369	-1307.059849	0.98286
	Weighted		-1307.059784	
cis-**C''** = *cis*-**C'**	REDO_6_srs_2	-1307.571325	-1307.096561	0.04083
	REDO_6_srs_3	-1307.577054	-1307.098066	0.20138
	REDO_6_srs_8	-1307.570884	-1307.094802	0.00632
	REDO_6_srs_9	-1307.570159	-1307.096270	0.03001
	REDO_6_ssr_1	-1307.575624	-1307.099195	0.66640
	REDO_6_ssr_5	-1307.573669	-1307.096797	0.05245
	REDO_6_ssr_6	-1307.569918	-1307.093965	0.00260
	Weighted		-1307.098605	
trans-**C''** = *trans*-**C'**	REDO_6_trans_1	-1307.579078	-1307.103246	0.56631
	REDO_6_trans_2	-1307.579099	-1307.102764	0.33959
	REDO_6_trans_3	-1307.576343	-1307.100887	0.04644
	REDO_6_trans_4	-1307.576169	-1307.098984	0.00618
	REDO_6_trans_8	-1307.576168	-1307.099021	0.00642
	REDO_6_trans_9	-1307.572932	-1307.100622	0.03506
	Weighted		-1307.102828	
**D**	REDO_b_2	-1307.585962	-1307.111155	0.51892
	REDO_b_3	-1307.583624	-1307.109812	0.12483
	REDO_b_4	-1307.585262	-1307.110427	0.23970

REDO_b_5	-1307.584096	-1307.108638	0.03598
REDO_b_6	-1307.581449	-1307.106698	0.00460
REDO_b_7	-1307.582839	-1307.109185	0.06422
REDO_b_8	-1307.583785	-1307.104616	0.00051
REDO_b_9	-1307.581240	-1307.107541	0.01124
Weighted		-1307.110531	

$\Delta G^{\ddagger}$ (Si, cyclic TS)	52.7	kJ/mol
$\Delta G^{\ddagger}$ (Si, open TS)	56.5	kJ/mol
$\Delta_r G^0$ (**A''**)	44.1	kJ/mol
$\Delta_r G^0$ (**B''**)	9.6	kJ/mol
$\Delta G^{\ddagger}$ (**A''** to *trans*-**C''**)	43.5	kJ/mol
$\Delta G^{\ddagger}$ (**B''** to *cis*-**C''**)	54.4	kJ/mol
$\Delta G^{\ddagger}$ (**B''** to **D**)	-55.5	kJ/mol
$\Delta_r G^0$ (*cis*-**C''**)	-157.4	kJ/mol
$\Delta_r G^0$ (*trans*-**C''**)	-168.5	kJ/mol
$\Delta_r G^0$ (**D**)	-188.7	kJ/mol

Note: The conversion of **B”** to *cis*-**C”** and **D** proceeds via dissociation of nitrogen and a subsequent rearrangement in a flat region of the potential energy surface. A transition state for the aryl shift (conversion to **D**) could be located by completely dissociating nitrogen from the end point of the IRC calculation (see Figure below) which is accordingly highly negative (-55.5 kJ/mol).

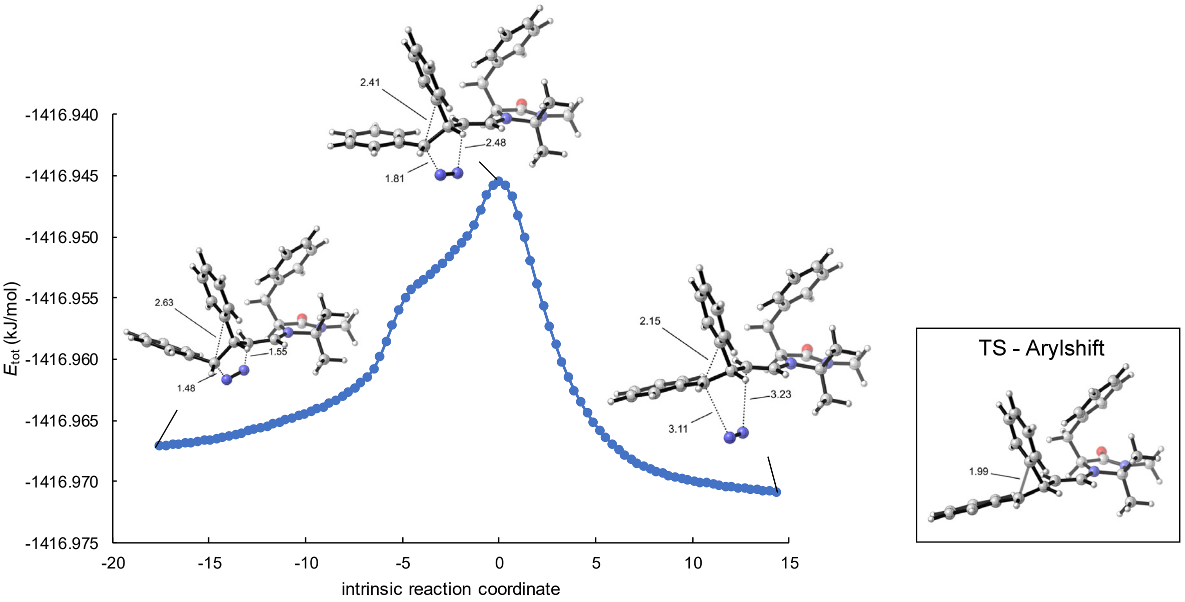

Table S17.

1a + 6a (Re attack)				
	Filename	E_{tot}	G_{298}	Weighting
**1a**	REDO_1a_1	-1037.381312	-1037.007311	0.42631
	REDO_1a_2	-1037.382784	-1037.007301	0.42156
	REDO_1a_4	-1037.376233	-1037.006339	0.15213
	Weighted		-1037.007159	
**6a**	REDO_3a_1	-379.564956	-379.482241	
Nitrogen	nitrogen	-109.434977	-109.447736	
cyclic TS (**1a** + **6a**, Re)	REDO_a_sr_5_ts1_rts1	-1416.944276	-1416.462804	0.644938
	REDO_a_sr_17_ts1_rts1_2	-1416.939845	-1416.460198	0.040689
	REDO_a_sr_19_cts1	-1416.941174	-1416.462127	0.31437
	Weighted		-1416.462485	
open TS (**1a** + **6a**, Re)	REDO_a_sr_3_ts1	-1416.939334	-1416.459932	0.00316
	REDO_a_sr_5_ts1	-1416.943321	-1416.464865	0.59068
	REDO_a_sr_5_ts1_rts3_2	-1416.942561	-1416.463975	0.23004
	REDO_a_sr_7_ts1	-1416.934147	-1416.456684	0.000101
	REDO_a_sr_14_ts1	-1416.935247	-1416.454869	0.000015
	REDO_a_sr_17_ts1	-1416.939429	-1416.459772	0.002670
	REDO_a_sr_17_ts1_rts2	-1416.940338	-1416.462752	0.062875
	REDO_a_sr_20_cts1	-1416.939463	-1416.459571	0.002158
	REDO_a_sr_34_fr_ts1	-1416.942674	-1416.463265	0.108303
	Weighted		-1416.464312	
**A'**	REDO_a_sr_3	-1416.945311	-1416.464163	0.025129
	REDO_a_sr_5	-1416.945975	-1416.464695	0.044175
	REDO_a_sr_5_rot1	-1416.945433	-1416.466972	0.493623
	REDO_a_sr_6	-1416.941133	-1416.461979	0.002480
	REDO_a_sr_7	-1416.936251	-1416.457079	0.000014
	REDO_a_sr_9	-1416.942351	-1416.461471	0.001448
	REDO_a_sr_10	-1416.935491	-1416.455753	0.000003
	REDO_a_sr_14	-1416.939132	-1416.458895	0.000094
	REDO_a_sr_16	-1416.942929	-1416.461273	0.001173
	REDO_a_sr_17	-1416.941395	-1416.466070	0.189745
	REDO_a_sr_18	-1416.933364	-1416.455116	0.000002
	REDO_a_sr_23	-1416.939874	-1416.459852	0.000260
	REDO_a_sr_34	-1416.945502	-1416.466225	0.223554
	REDO_a_sr_35	-1416.941375	-1416.463240	0.009440
	REDO_a_sr_54	-1416.940913	-1416.460784	0.000699
	REDO_a_sr_58	-1416.944281	-1416.463062	0.007816

	REDO_a_sr_94_freq	-1416.940923	-1416.460061	0.000325
	REDO_a_sr_97	-1416.935056	-1416.457364	0.000019
	Weighted		-1416.466360	
**B'**	REDO_6_si_srs_2	-1416.969927	-1416.483081	0.76608
	REDO_6_si_srs_4	-1416.968484	-1416.479834	0.02449
	REDO_6_si_srs_5	-1416.967711	-1416.480101	0.03251
	REDO_6_si_srs_6	-1416.964355	-1416.476538	0.00074
	REDO_6_si_srs_7	-1416.963314	-1416.475622	0.00028
	REDO_6_si_srs_8	-1416.963666	-1416.477679	0.00249
	REDO_6_si_srs_9	-1416.965314	-1416.476472	0.00069
	REDO_6_si_srs_10	-1416.964176	-1416.479695	0.02113
	REDO_6_si_srs_11	-1416.961615	-1416.477273	0.00162
	REDO_6_si_srs_12	-1416.962338	-1416.476624	0.00081
	REDO_6_si_srs_13	-1416.968162	-1416.479496	0.01711
	REDO_a_sr_2	-1416.965572	-1416.476312	0.00059
	REDO_a_sr_4	-1416.966432	-1416.480907	0.07636
	REDO_a_sr_19_2	-1416.965899	-1416.480599	0.05508
	Weighted		-1416.482426	
TS (**A'** to trans-**C'**)	REDO_a_sr_5_rot1_cp_ts1_4	-1416.942622	-1416.463733	1.00000
	Weighted		-1416.463733	
TS (**B'** to cis-**C'**)	exo_pes_244_178_ts1	-1416.950319	-1416.470364	0.98338
	REDO_6_si_srs_3_n2_ts1	-1416.945109	-1416.466513	0.01658
	REDO_6_si_srs_9_n2_ts1	-1416.943179	-1416.460510	0.00003
	REDO_6_si_srs_8_n2_ts1	-1416.936458	-1416.459654	0.00001
	Weighted		-1416.470300	
TS: **B'** to **D**	6_si_srs_9_n2_ts1_cshift_ts1	-1307.527351	-1307.054465	1.00000
	Weighted		-1307.054465	
cis-**C''** = cis-**C'**	REDO_6_srs_2	-1307.571325	-1307.096561	0.04083
	REDO_6_srs_3	-1307.577054	-1307.098066	0.20138
	REDO_6_srs_8	-1307.570884	-1307.094802	0.00632
	REDO_6_srs_9	-1307.570159	-1307.096270	0.03001
	REDO_6_ssr_1	-1307.575624	-1307.099195	0.66640
	REDO_6_ssr_5	-1307.573669	-1307.096797	0.05245
	REDO_6_ssr_6	-1307.569918	-1307.093965	0.00260
	Weighted		-1307.098605	
trans-**C''** = trans-**C'**	REDO_6_trans_1	-1307.579078	-1307.103246	0.56631
	REDO_6_trans_2	-1307.579099	-1307.102764	0.33959
	REDO_6_trans_3	-1307.576343	-1307.100887	0.04644

	REDO_6_trans_4	-1307.576169	-1307.098984	0.00618
	REDO_6_trans_8	-1307.576168	-1307.099021	0.00642
	REDO_6_trans_9	-1307.572932	-1307.100622	0.03506
	Weighted		-1307.102828	
**D**	REDO_b_2	-1307.585962	-1307.111155	0.51892
	REDO_b_3	-1307.583624	-1307.109812	0.12483
	REDO_b_4	-1307.585262	-1307.110427	0.23970
	REDO_b_5	-1307.584096	-1307.108638	0.03598
	REDO_b_6	-1307.581449	-1307.106698	0.00460
	REDO_b_7	-1307.582839	-1307.109185	0.06422
	REDO_b_8	-1307.583785	-1307.104616	0.00051
	REDO_b_9	-1307.581240	-1307.107541	0.01124
	Weighted		-1307.110531	

$\Delta G^{‡}$ (Re, cyclic TS)	62.8	kJ/mol
$\Delta G^{‡}$ (Re, open TS)	58.0	kJ/mol
$\Delta_r G^0$ (**A'**)	52.6	kJ/mol
$\Delta_r G^0$ (**B'**)	10.4	kJ/mol
$\Delta G^{‡}$ (**A'** to *trans*-**C'**)	59.5	kJ/mol
$\Delta G^{‡}$ (**B'** to *cis*-**C'**)	42.2	kJ/mol
$\Delta G^{‡}$ (**B'** to **D**)	-41.5	kJ/mol
$\Delta_r G^0$ (*cis*-**C'**)	-157.4	kJ/mol
$\Delta_r G^0$ (*trans*-**C'**)	-168.5	kJ/mol
$\Delta_r G^0$ (**D**)	-188.7	kJ/mol

Note: Depending on the conformation of **B'**, nitrogen dissociation results in a flat PES from which either cyclopropanation or aryl shift occurs. While some IRC calculations from the cycloreversion TS directly yield the cyclopropane (see Figure below), a transition state for the aryl shift (conversion to **D**) could only be located by completely dissociating nitrogen from the end point of the IRC calculation (see Figure below) which is accordingly highly negative (-41.5 kJ/mol).

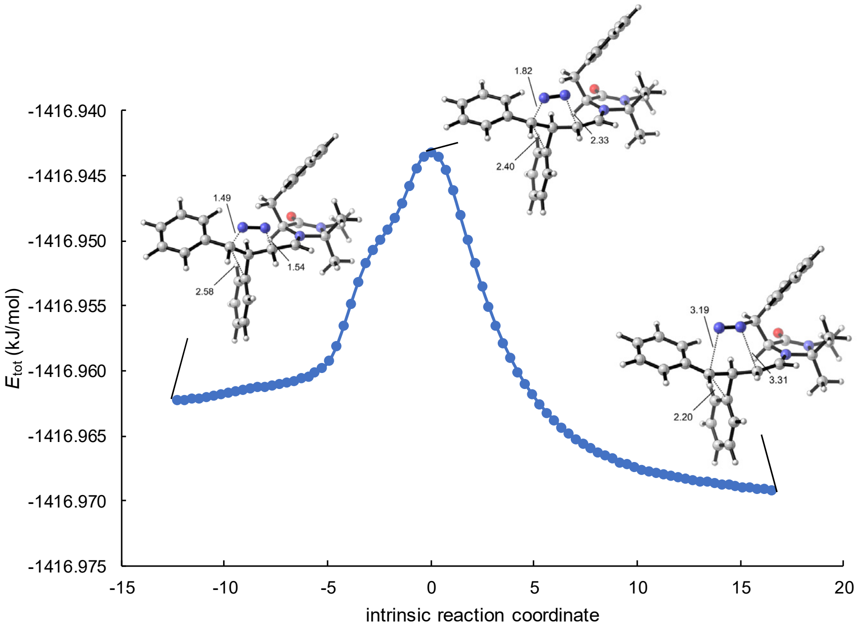

-1416.940
-1416.945
-1416.950
-1416.955
-1416.960
-1416.965
-1416.970
-1416.975
E_{tot} (kJ/mol)
-15
-10
-5
0
5
10
15
20
intrinsic reaction coordinate
1.49
1.54
2.58
1.82
2.33
2.40
3.19
3.31
2.20

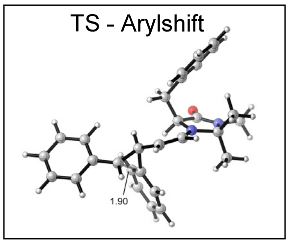

TS - Arylshift
1.90

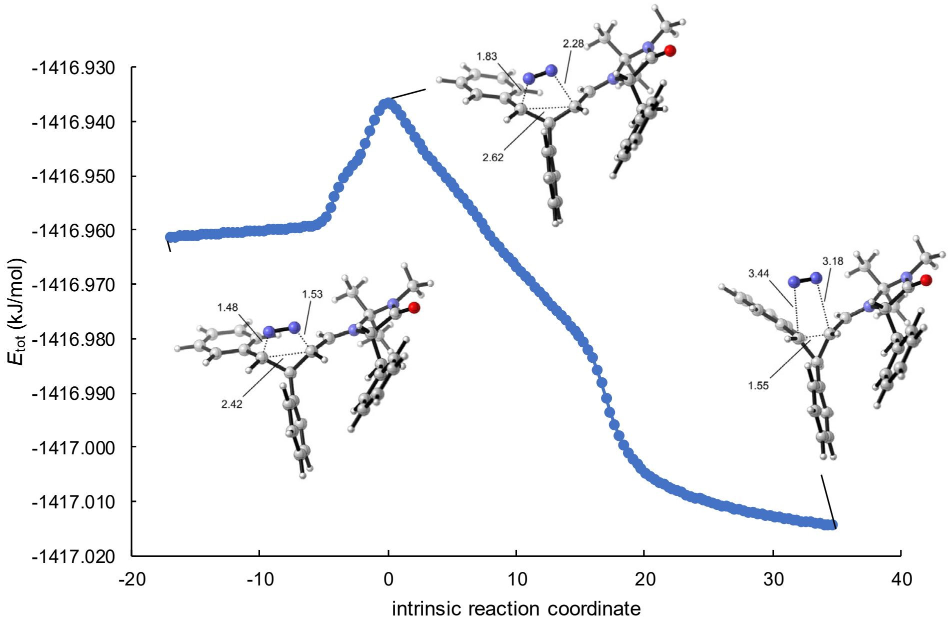

-1416.930
-1416.940
-1416.950
-1416.960
-1416.970
-1416.980
-1416.990
-1417.000
-1417.010
-1417.020
E_{tot} (kJ/mol)
-20
-10
0
10
20
30
40
intrinsic reaction coordinate
1.83
2.28
2.62
1.48
1.53
2.42
3.44
3.18
1.55

Table S18.

D + 6a (Si attack)				
	Filename	E_{tot}	G_{298}	Weighting
**D**	REDO_b_2	-1307.585962	-1307.111155	0.51850
	REDO_b_3	-1307.583624	-1307.109812	0.12473
	REDO_b_4	-1307.585262	-1307.110427	0.23951
	REDO_b_5	-1307.584096	-1307.108638	0.03595
	REDO_b_6	-1307.581449	-1307.106698	0.00460
	REDO_b_7	-1307.582839	-1307.109185	0.06417
	REDO_b_8	-1307.583785	-1307.104616	0.00051
	REDO_b_9	-1307.581240	-1307.107541	0.01123
	REDO_b_10	-1307.581628	-1307.105046	0.00080
	Weighted		-1307.110527	
**6a**	REDO_3a_1	-379.564956	-379.482241	
Nitrogen	nitrogen	-109.434977	-109.447736	
cyclic TS (**D** + **6a**, Si)	REDO_si_c_17_ts1_rts1	-1687.158204	-1686.576126	1.00000
	Weighted		-1686.576126	
open TS (**D** + **6a**, Si)	REDO_si_c_17_ts1	-1687.154666	-1686.572887	0.99833
	REDO_si_c_18_fr_ts1	-1687.149706	-1686.566855	0.00167
	Weighted		-1686.572877	
open IM (Si)	REDO_si_c_1	-1687.161412	-1686.580050	0.069458
	REDO_si_c_3	-1687.156241	-1686.570323	0.000002
	REDO_si_c_5	-1687.151238	-1686.568203	0.000000
	REDO_si_c_6	-1687.150570	-1686.570269	0.000002
	REDO_si_c_7	-1687.150977	-1686.567729	0.000000
	REDO_si_c_8	-1687.156410	-1686.573590	0.000074
	REDO_si_c_9	-1687.156417	-1686.574249	0.000148
	REDO_si_c_10	-1687.159772	-1686.575683	0.000678
	REDO_si_c_17	-1687.164974	-1686.582494	0.92750
	REDO_si_c_18	-1687.158271	-1686.575876	0.00083
	REDO_si_c_21	-1687.158705	-1686.576293	0.00129
	REDO_si_c_25	-1687.157311	-1686.571433	0.00001
	REDO_si_c_27	-1687.153640	-1686.570962	0.00000
	REDO_si_c_38	-1687.151439	-1686.567937	0.00000
	Weighted		-1686.582304	
cyclic IM (Si)	REDO_2d_endo_1	-1687.186117	-1686.591830	0.02553
	REDO_2d_endo_2_2	-1687.185557	-1686.595194	0.90303
	REDO_2d_endo_3	-1687.182892	-1686.592801	0.07144

	Weighted		-1686.594937	
TS (open IM to *trans*-**E**)	REDO_si_c_17_cts1	-1687.164106	-1686.582653	0.99848
	REDO_si_c_18_cts1	-1687.157847	-1686.576530	0.00151
	REDO_si_c_27_cts1	-1687.153151	-1686.571703	0.00001
	Weighted		-1686.582643	
TS (cyclic IM to cis-**E**)	pes_2d_endo_3_240_181_ts1_3	-1687.158625	-1686.574790	0.01041
	pes_2d_endo_1_239_185_ts1	-1687.162053	-1686.579086	0.98959
	Weighted		-1686.579041	
cis-**E**	REDO_5_cis_1	-1577.792207	-1577.214290	0.25631
	REDO_5_cis_4	-1577.788090	-1577.211066	0.00839
	REDO_REDO_5_cis_5	-1577.791056	-1577.212496	0.03822
	REDO_REDO_5_cis_6	-1577.792207	-1577.215229	0.69340
	REDO_REDO_5_cis_9	-1577.788099	-1577.210286	0.00367
	Weighted		-1577.214831	
trans-**E**	REDO_5_trans_1	-1577.782102	-1577.205274	0.00003
	REDO_5_trans_2	-1577.793810	-1577.214163	0.36499
	REDO_REDO_5_trans_5	-1577.793451	-1577.214091	0.33831
	REDO_REDO_5_trans_7	-1577.793809	-1577.213968	0.29667
	Weighted		-1577.214081	

$\Delta G^{\ddagger}$ (Si, cyclic TS)	35.8	kJ/mol
$\Delta G^{\ddagger}$ (Si, open TS)	44.3	kJ/mol
$\Delta_r G^0$ (Si, open IM)	19.6	kJ/mol
$\Delta_r G^0$ (Si, cyclic IM)	-13.6	kJ/mol
$\Delta G^{\ddagger}$ (open IM to *trans*-**E**)	18.7	kJ/mol
$\Delta G^{\ddagger}$ (cyclic IM to *cis*-**E**)	28.1	kJ/mol
$\Delta_r G^0$ (*cis*-**E**)	-191.2	kJ/mol
$\Delta_r G^0$ (*trans*-**E**)	-189.2	kJ/mol

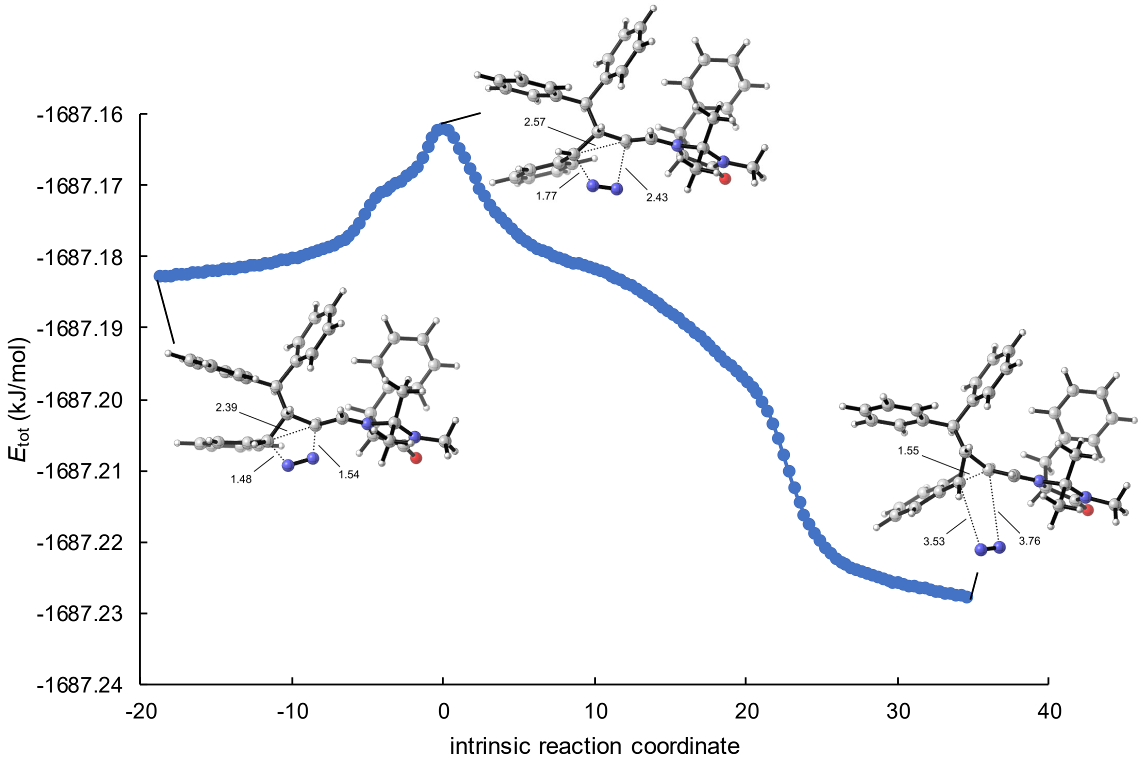
-1687.16
-1687.17
-1687.18
-1687.19
-1687.20
-1687.21
-1687.22
-1687.23
-1687.24
E_{tot} (kJ/mol)
-20
-10
0
10
20
30
40
intrinsic reaction coordinate
2.57
1.77
2.43
2.39
1.48
1.54
1.55
3.53
3.76

Table S19.

D + 6a (Re attack)				
	Filename	E_{tot}	G_{298}	Weighting
**D**	REDO_b_2	-1307.585962	-1307.111155	0.51850
	REDO_b_3	-1307.583624	-1307.109812	0.12473
	REDO_b_4	-1307.585262	-1307.110427	0.23951
	REDO_b_5	-1307.584096	-1307.108638	0.03595
	REDO_b_6	-1307.581449	-1307.106698	0.00460
	REDO_b_7	-1307.582839	-1307.109185	0.06417
	REDO_b_8	-1307.583785	-1307.104616	0.00051
	REDO_b_9	-1307.581240	-1307.107541	0.01123
	REDO_b_10	-1307.581628	-1307.105046	0.00080
	Weighted		-1307.110527	
**6a**	REDO_3a_1	-379.564956	-379.482241	
Nitrogen	nitrogen	-109.434977	-109.447736	
cyclic TS (**D** + **6a**, Re)	REDO_re_c_10_ts1_rts1	-1687.154500	-1686.573213	1.00000
	Weighted		-1686.573213	
open TS (**D** + **6a**, Re)	REDO_re_c_10_ts1	-1687.152449	-1686.570260	0.99861
	REDO_re_c_10_rot1_fr_ts1	-1687.147597	-1686.564057	0.00139
	Weighted		-1686.570251	
open IM (Re)	REDO_re_c_1	-1687.154400	-1686.569959	0.00011
	REDO_re_c_4	-1687.158644	-1686.573880	0.00682
	REDO_re_c_5	-1687.158636	-1686.577974	0.52277
	REDO_re_c_8	-1687.155525	-1686.571213	0.00040
	REDO_re_c_10	-1687.162607	-1686.577872	0.46915
	REDO_re_c_15	-1687.152938	-1686.569078	0.00004
	REDO_re_c_16	-1687.154763	-1686.568940	0.00004
	REDO_re_c_20	-1687.153720	-1686.570928	0.00030
	REDO_re_c_10_rot1	-1687.154659	-1686.571175	0.00039
	REDO_re_c_20_rot1	-1687.142954	-1686.562199	0.00000
	Weighted		-1686.577889	
cyclic IM (Re)	REDO_2d_exo_1	-1687.185552	-1686.594803	0.06848
	REDO_2d_exo_2	-1687.186018	-1686.594996	0.08401
	REDO_2d_exo_4	-1687.185558	-1686.594996	0.08405
	REDO_REDO_2d_exo_6	-1687.184867	-1686.597073	0.75975
	REDO_REDO_2d_exo_7	-1687.181628	-1686.588739	0.00011
	REDO_REDO_2d_exo_8	-1687.180694	-1686.590579	0.00078
	REDO_REDO_2d_exo_9	-1687.183072	-1686.591310	0.00169

	REDO_REDO_2d_exo_10	-1687.181949	-1686.590942	0.00114
	Weighted		-1686.596546	
TS (open IM to trans-**E**)	REDO_re_c_10_rot1_cpts1	-1687.151571	-1686.569869	1.00000
	Weighted		-1686.569869	
TS (cyclic IM to cis-**E**)	pes_2d_exo_2_240_181_ts1_freq	-1687.163765	-1686.583919	0.99991
	REDO_2d_exo_1_n2_ts1	-1687.157723	-1686.575144	0.00009
	Weighted		-1686.583918	
cis-**E**	REDO_5_cis_1	-1577.792207	-1577.214290	0.25631
	REDO_5_cis_4	-1577.788090	-1577.211066	0.00839
	REDO_REDO_5_cis_5	-1577.791056	-1577.212496	0.03822
	REDO_REDO_5_cis_6	-1577.792207	-1577.215229	0.69340
	REDO_REDO_5_cis_9	-1577.788099	-1577.210286	0.00367
	Weighted		-1577.214831	
trans-**E**	REDO_5_trans_1	-1577.782102	-1577.205274	0.00003
	REDO_5_trans_2	-1577.793810	-1577.214163	0.36499
	REDO_REDO_5_trans_5	-1577.793451	-1577.214091	0.33831
	REDO_REDO_5_trans_7	-1577.793809	-1577.213968	0.29667
	Weighted		-1577.214081	

$\Delta G^{\ddagger}$ (Re, cyclic TS)	43.4	kJ/mol
$\Delta G^{\ddagger}$ (Re, open TS)	51.2	kJ/mol
$\Delta_r G^0$ (Re, open IM)	31.2	kJ/mol
$\Delta_r G^0$ (Re, cyclic IM)	-17.8	kJ/mol
$\Delta G^{\ddagger}$ (open IM to *cis*-**E**)	52.2	kJ/mol
$\Delta G^{\ddagger}$ (cyclic IM to *trans*-**E**)	15.3	kJ/mol
$\Delta_r G^0$ (*cis*-**E**)	-191.2	kJ/mol
$\Delta_r G^0$ (*trans*-**E**)	-189.2	kJ/mol

11. Copies of NMR Spectra

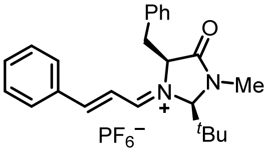

2a

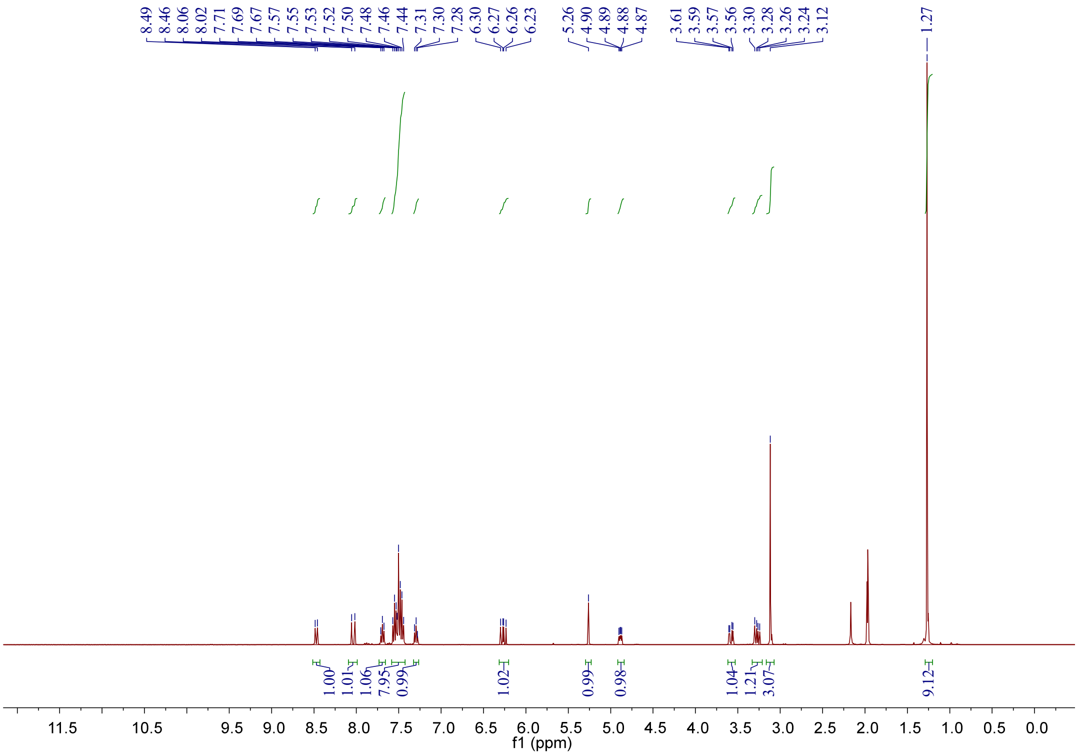

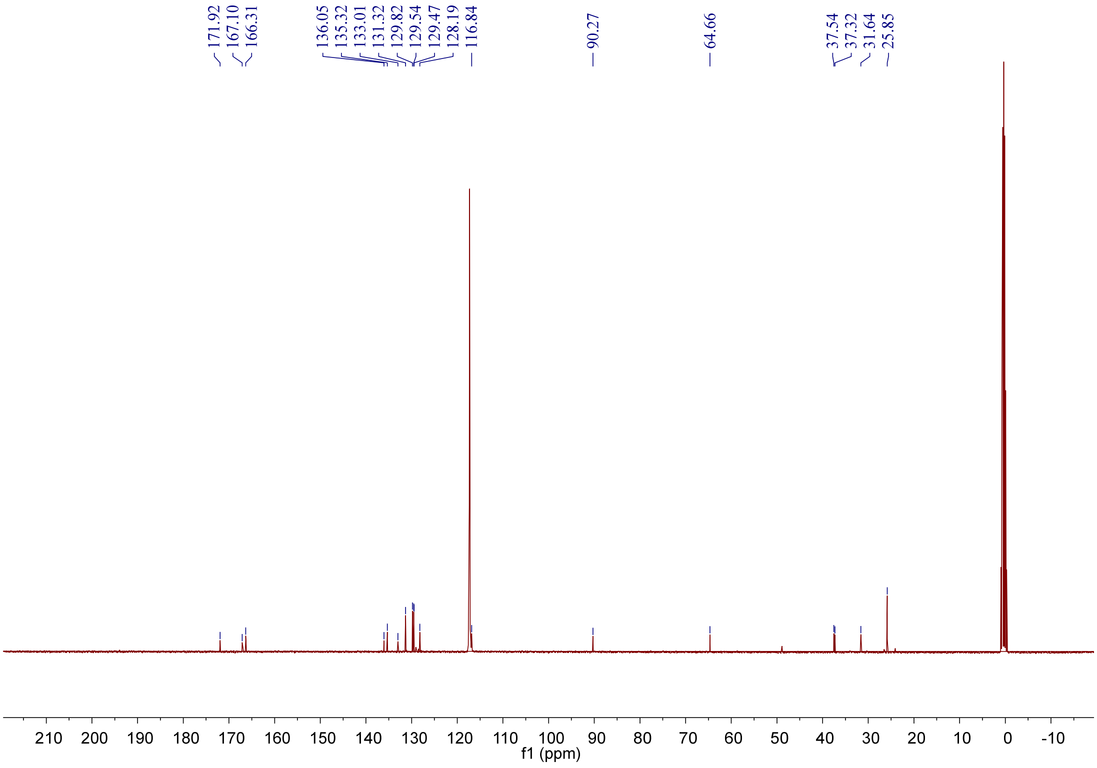

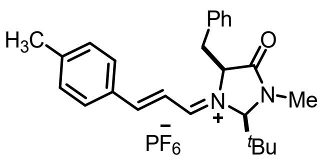
Ph
O
H3C
N
N-Me
PF6
tBu

2b

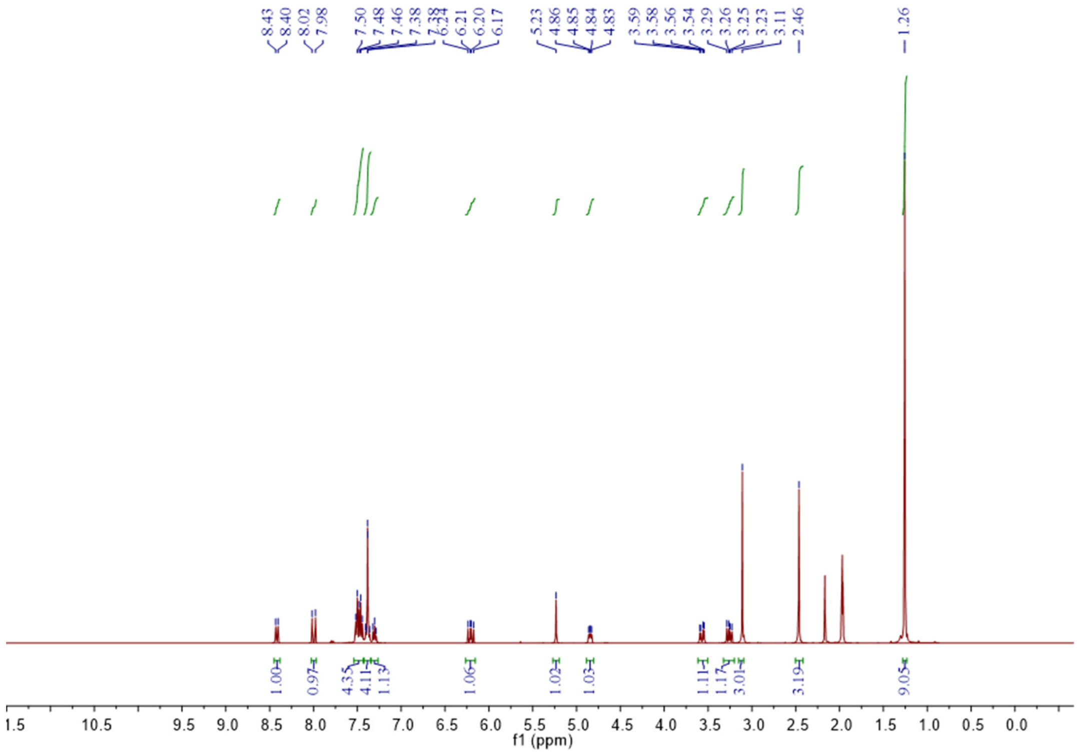
8.43
8.40
8.02
7.98
7.50
7.48
7.46
7.38
7.34
6.24
6.21
6.20
6.17
5.23
4.86
4.85
4.84
4.83
3.59
3.58
3.56
3.54
3.29
3.26
3.25
3.23
3.11
2.46
1.26
1.00
0.97
4.35
4.11
1.13
1.06
1.02
1.03
1.11
1.17
3.01
3.19
9.05
f1 (ppm)

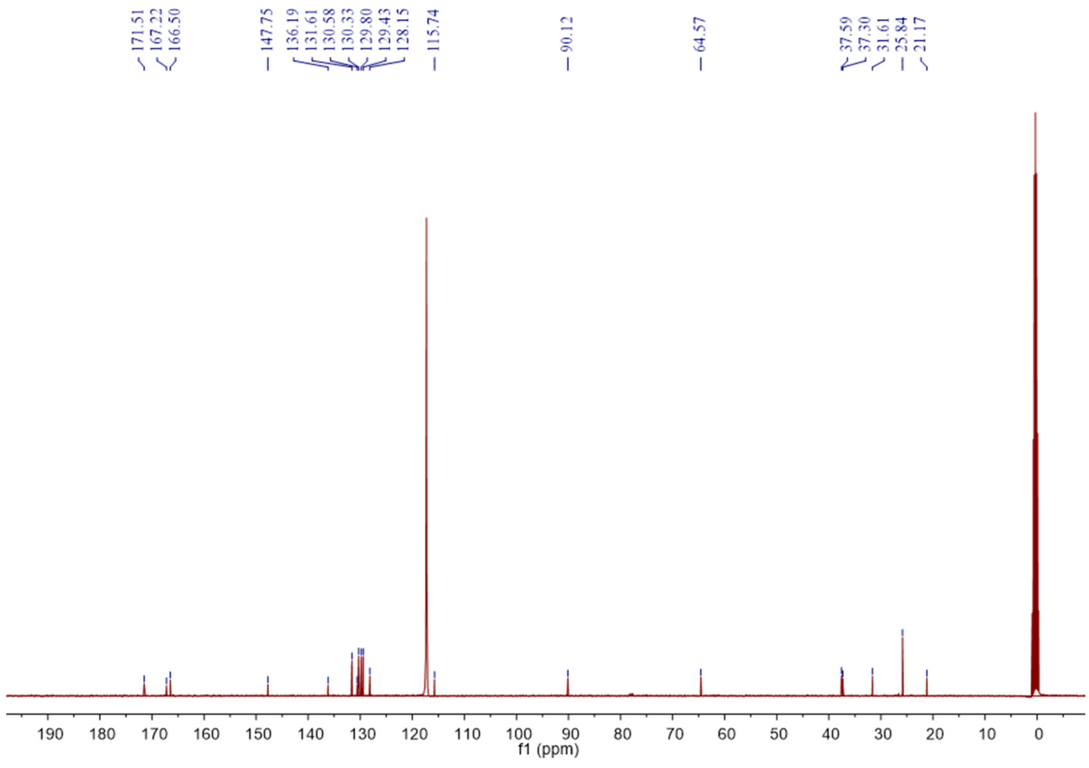
171.51
167.22
166.50
147.75
136.19
131.61
130.58
130.33
129.80
129.43
128.15
115.74
90.12
64.57
37.59
37.30
31.61
25.84
21.17
f1 (ppm)

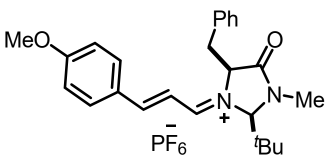
MeO
Ph
O
N
N-Me
+
PF_6^-
^{t}Bu

2c

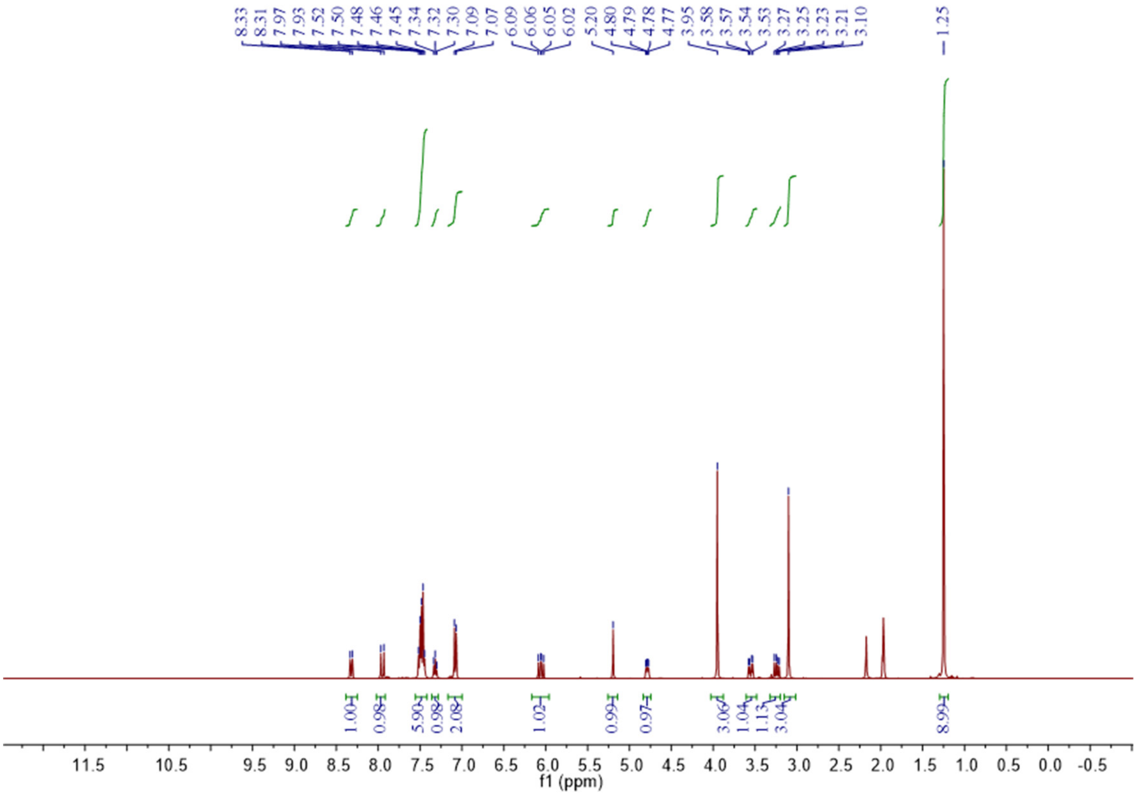
8.33 8.31 7.97 7.93 7.52 7.50 7.48 7.46 7.45 7.34 7.32 7.30 7.09 7.07 6.09 6.06 6.05 6.02 5.20 4.80 4.79 4.78 4.77 3.95 3.58 3.57 3.54 3.53 3.27 3.25 3.23 3.21 3.10 1.25
1.00 0.98 5.90 0.98 2.08 1.02 0.99 0.97 3.06 1.04 1.15 3.04 8.99
11.5 10.5 9.5 9.0 8.5 8.0 7.5 7.0 6.5 6.0 5.5 5.0 4.5 4.0 3.5 3.0 2.5 2.0 1.5 1.0 0.5 0.0 -0.5
f1 (ppm)

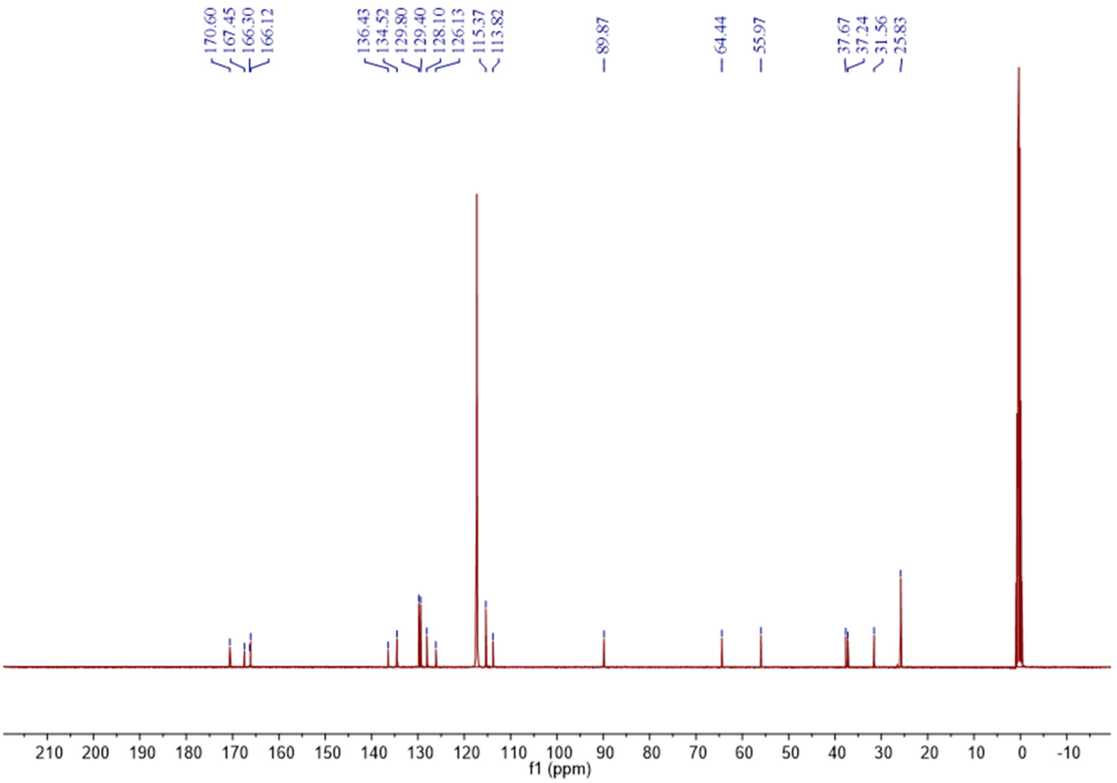
170.60 167.45 166.30 166.12 136.43 134.52 129.80 129.40 128.10 126.13 115.37 113.82 89.87 64.44 55.97 37.67 37.24 31.56 25.83
210 200 190 180 170 160 150 140 130 120 110 100 90 80 70 60 50 40 30 20 10 0 -10
f1 (ppm)

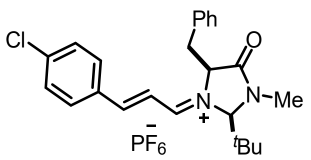
Cl
Ph
O
N
N-Me
+
PF6
tBu

2e

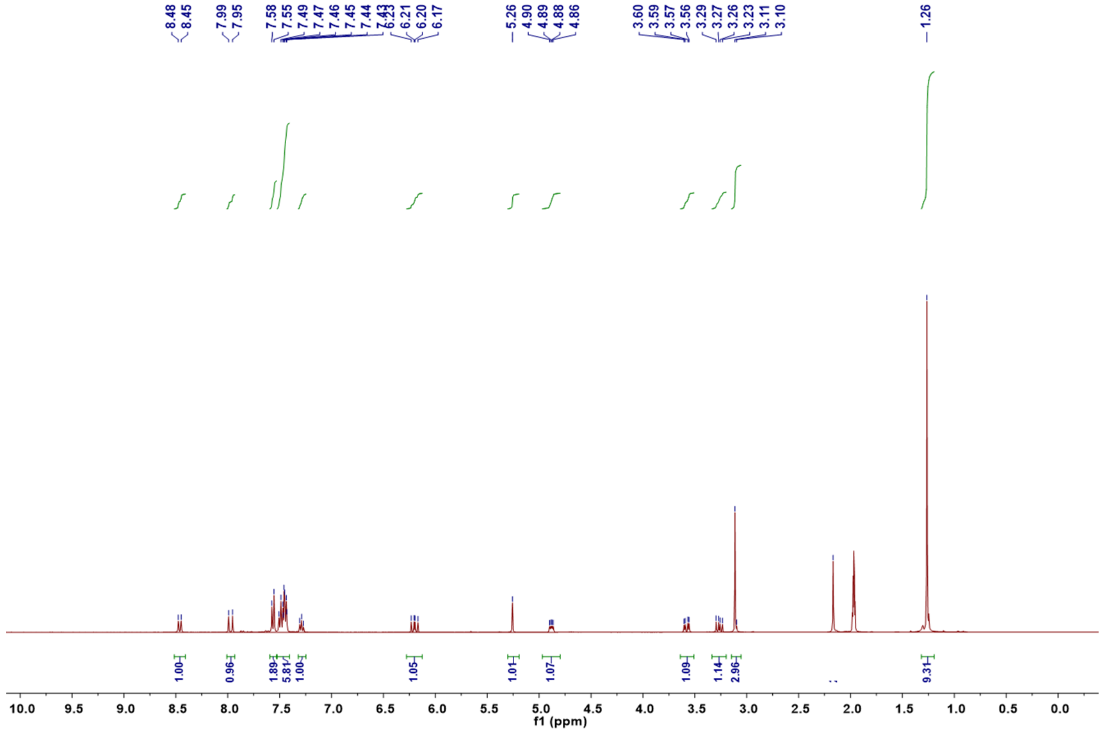
8.48
8.45
7.99
7.95
7.58
7.55
7.49
7.47
7.46
7.45
7.44
7.43
6.23
6.21
6.20
6.17
5.26
4.90
4.89
4.88
4.86
3.60
3.59
3.57
3.56
3.29
3.27
3.26
3.23
3.11
3.10
1.26
1.00
0.96
1.89
5.81
1.00
1.05
1.01
1.07
1.09
1.14
2.96
9.31
f1 (ppm)

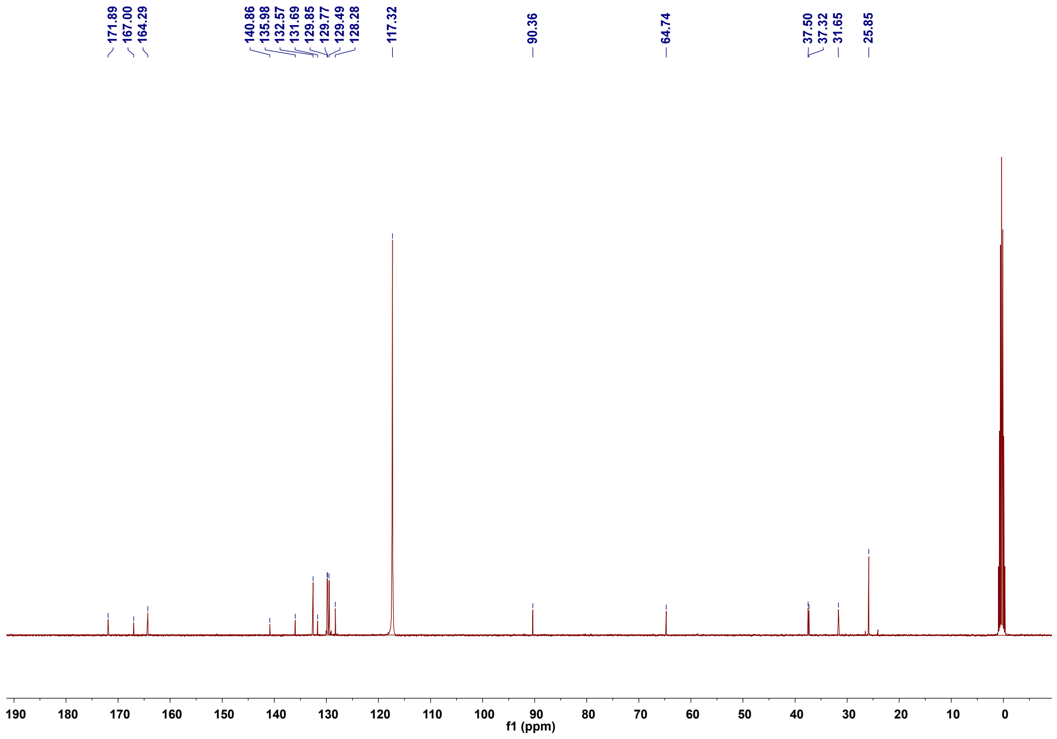
171.89
167.00
164.29
140.86
135.98
132.57
131.69
129.85
129.77
129.49
128.28
117.32
90.36
64.74
37.50
37.32
31.65
25.85
f1 (ppm)

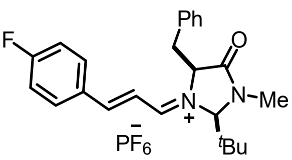
F
Ph
O
N
Me
N
tBu
PF6

2f

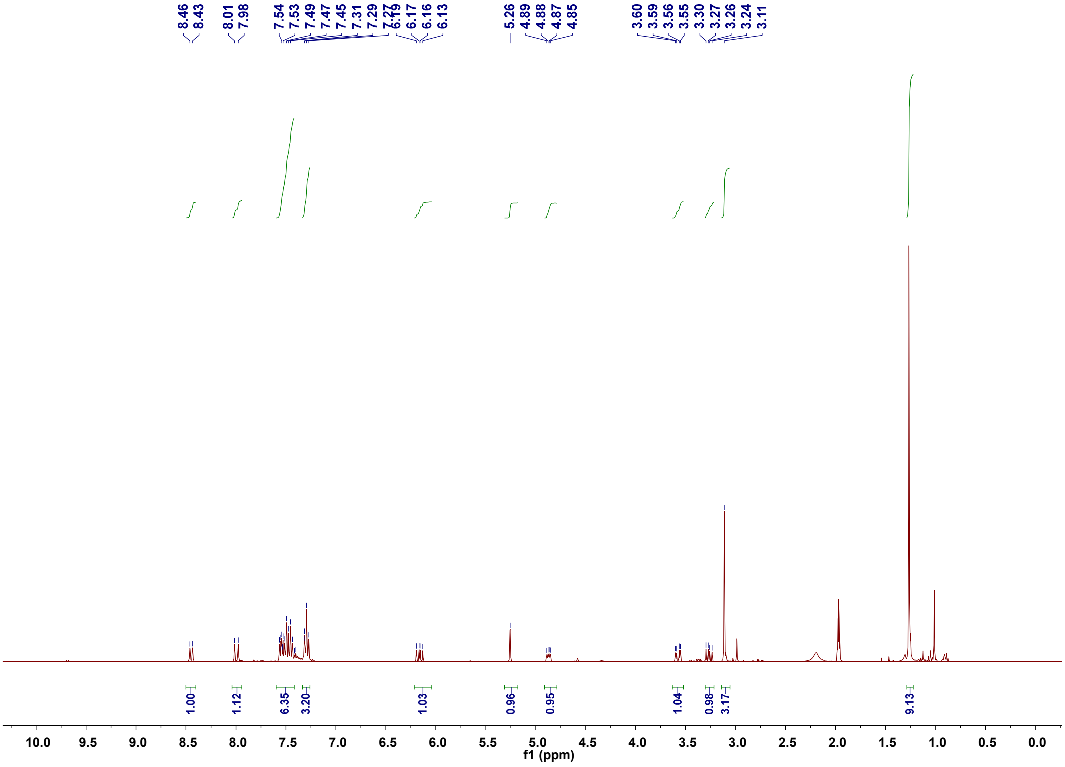
8.46
8.43
8.01
7.98
7.54
7.53
7.49
7.47
7.45
7.31
7.29
6.17
6.16
6.13
5.26
4.89
4.88
4.87
4.85
3.60
3.59
3.56
3.55
3.30
3.27
3.26
3.24
3.11
1.00
1.12
6.35
3.20
1.03
0.96
0.95
1.04
0.98
3.17
9.13
f1 (ppm)

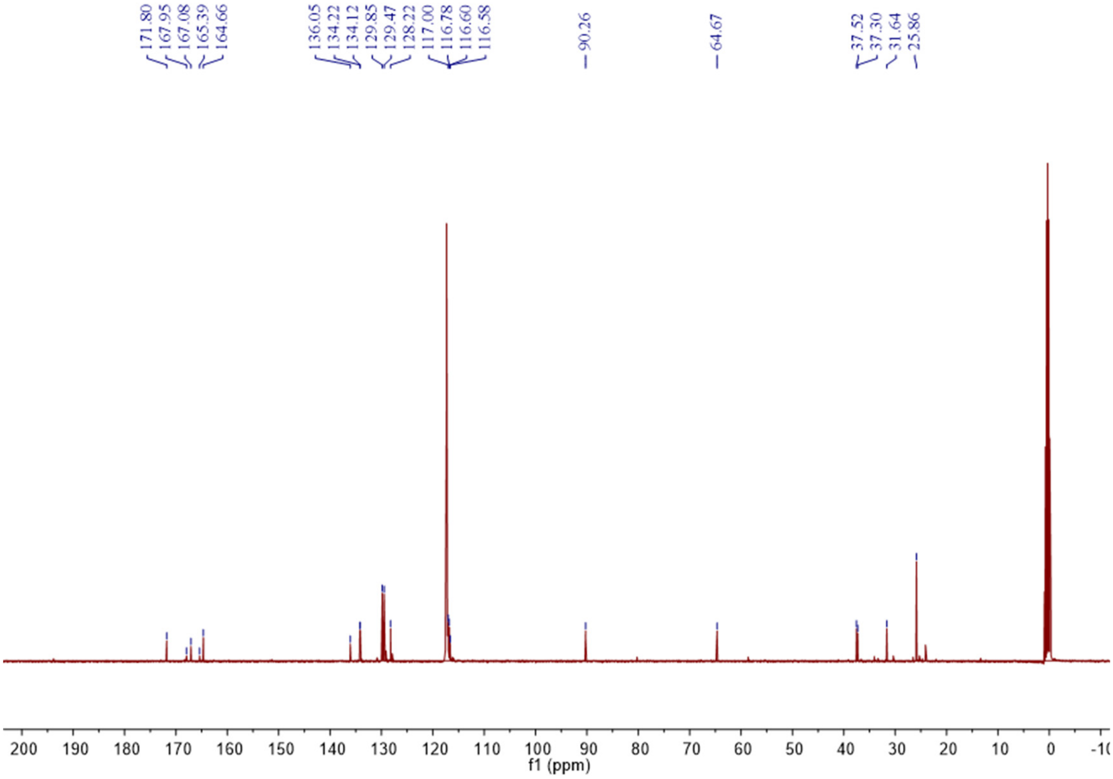
171.80
167.95
167.08
165.39
164.66
136.05
134.22
134.12
129.85
129.47
128.22
117.00
116.78
116.60
116.58
90.26
64.67
37.52
37.30
31.64
25.86
f1 (ppm)

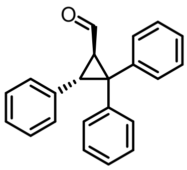
O

5a

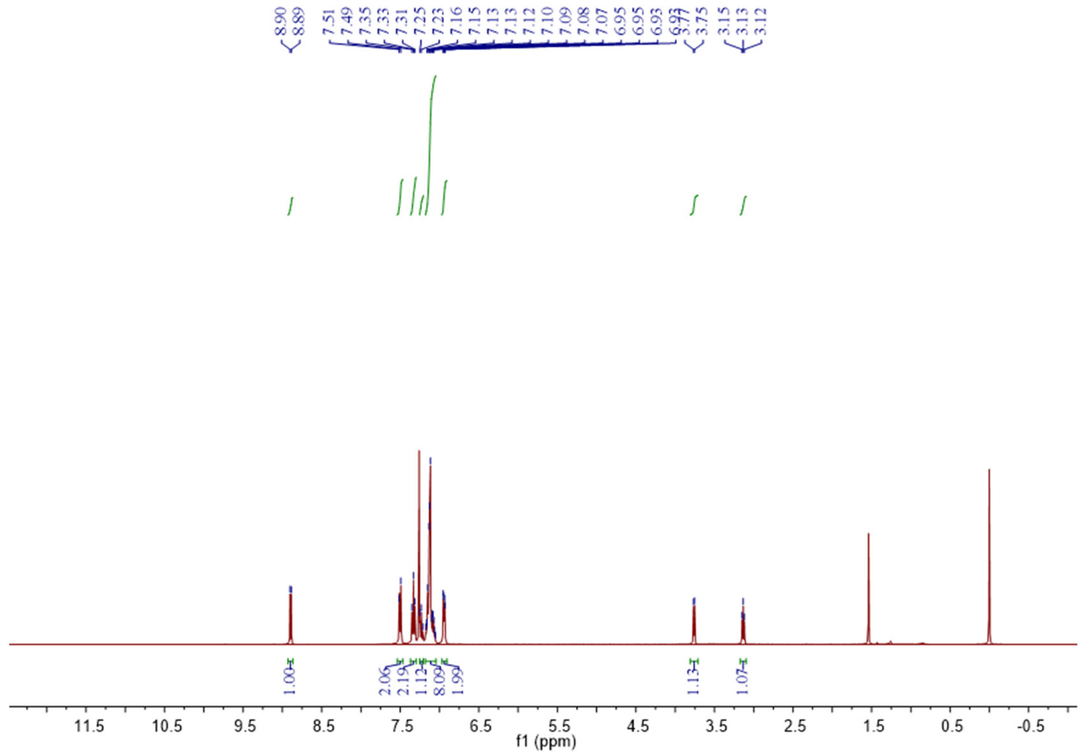
8.90
8.89
7.51
7.49
7.35
7.33
7.31
7.25
7.23
7.16
7.15
7.13
7.13
7.12
7.10
7.09
7.08
7.07
6.95
6.95
6.93
6.93
3.77
3.75
3.15
3.13
3.12
1.00
2.06
2.19
1.12
8.09
1.99
1.13
1.07
11.5
10.5
9.5
8.5
7.5
6.5
5.5
4.5
3.5
2.5
1.5
0.5
-0.5
f1 (ppm)

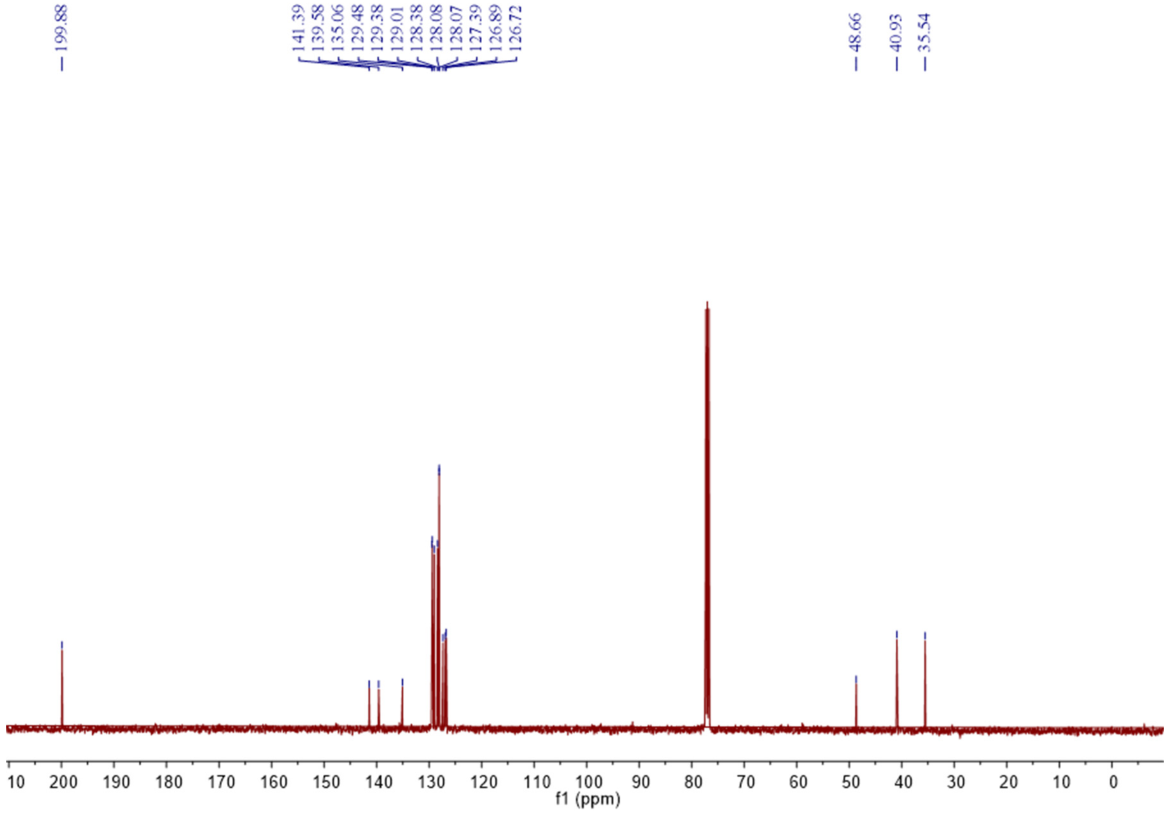
199.88
141.39
139.58
135.06
129.48
129.38
129.01
128.38
128.08
128.07
127.39
126.89
126.72
48.66
40.93
35.54
10
200
190
180
170
160
150
140
130
120
110
100
90
80
70
60
50
40
30
20
10
0
f1 (ppm)

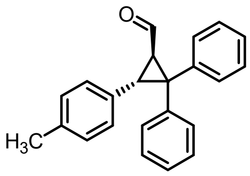
O
H3C

5b

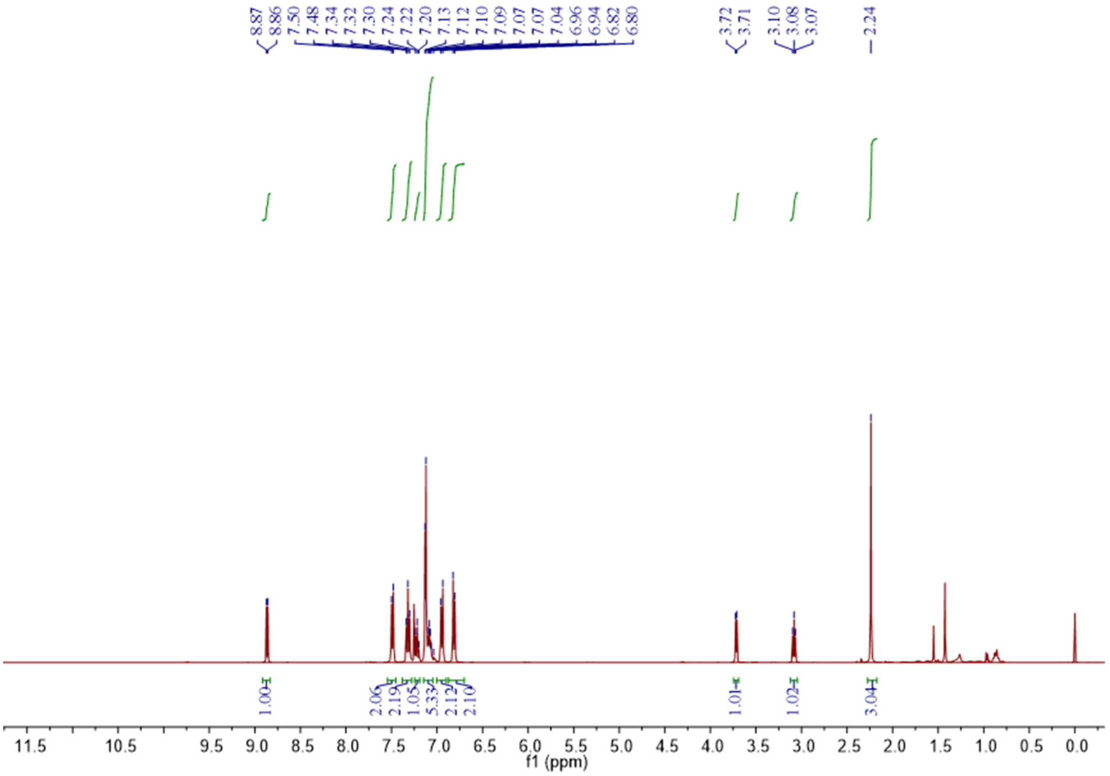
8.87
8.86
7.50
7.48
7.34
7.32
7.30
7.24
7.22
7.20
7.13
7.12
7.10
7.09
7.07
7.07
7.04
6.96
6.94
6.82
6.80
3.72
3.71
3.10
3.08
3.07
2.24
1.00
2.06
2.19
1.05
5.33
2.12
2.10
1.01
1.02
3.04
11.5 10.5 9.5 9.0 8.5 8.0 7.5 7.0 6.5 6.0 5.5 5.0 4.5 4.0 3.5 3.0 2.5 2.0 1.5 1.0 0.5 0.0
f1 (ppm)

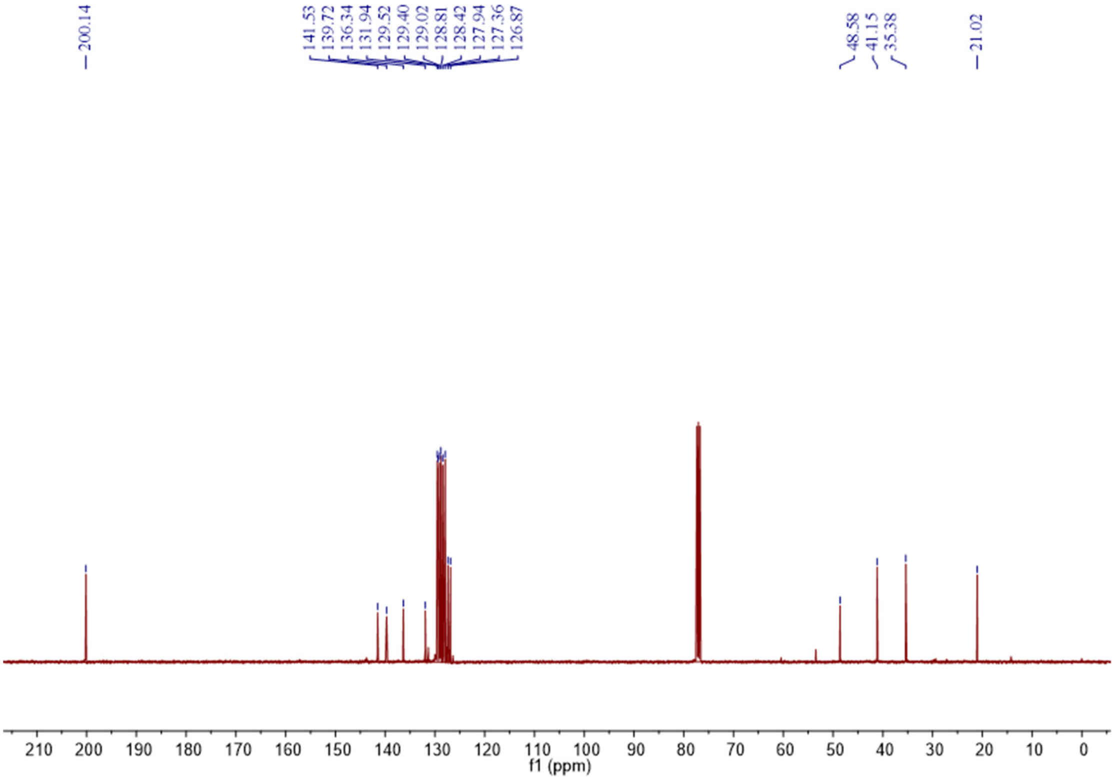
200.14
141.53
139.72
136.34
131.94
129.52
129.40
129.02
128.81
128.42
127.94
127.36
126.87
48.58
41.15
35.38
21.02
210 200 190 180 170 160 150 140 130 120 110 100 90 80 70 60 50 40 30 20 10 0
f1 (ppm)

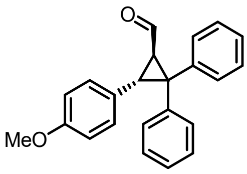
MeO
O

5c

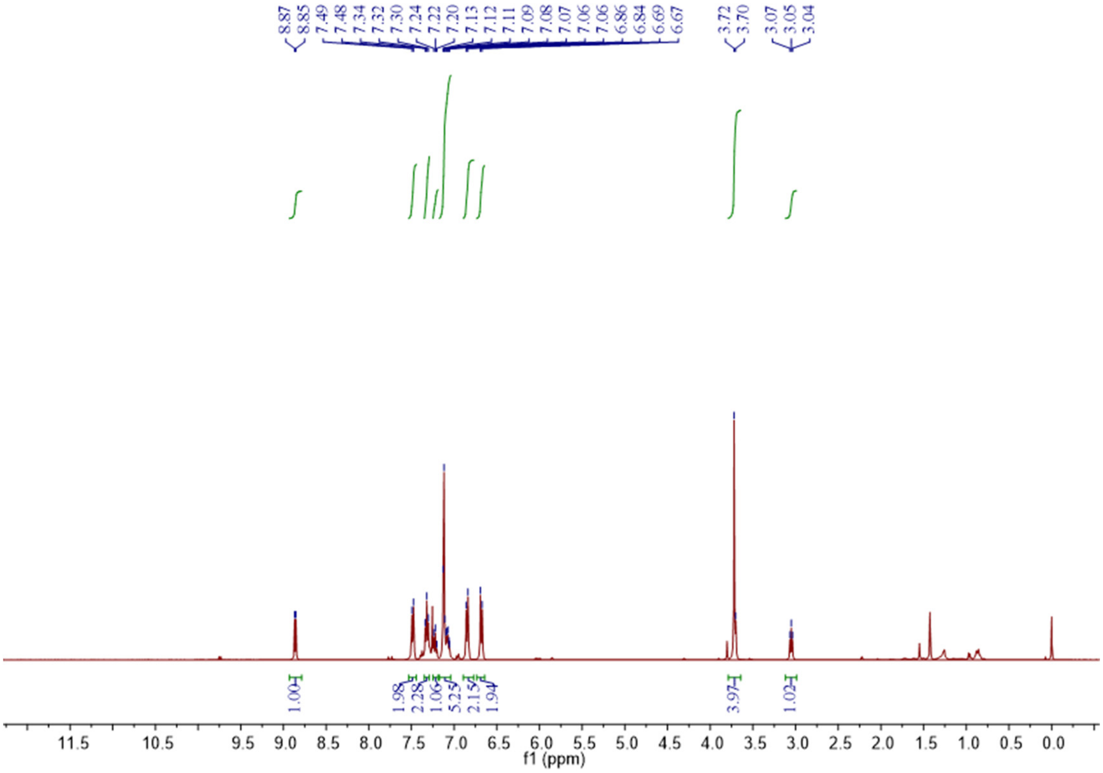
8.87
8.85
7.49
7.48
7.34
7.32
7.30
7.24
7.22
7.20
7.13
7.12
7.11
7.09
7.08
7.07
7.06
7.06
6.86
6.84
6.69
6.67
3.72
3.70
3.07
3.05
3.04
1.00
1.98
2.28
1.06
5.25
2.15
1.94
3.97
1.02
f1 (ppm)

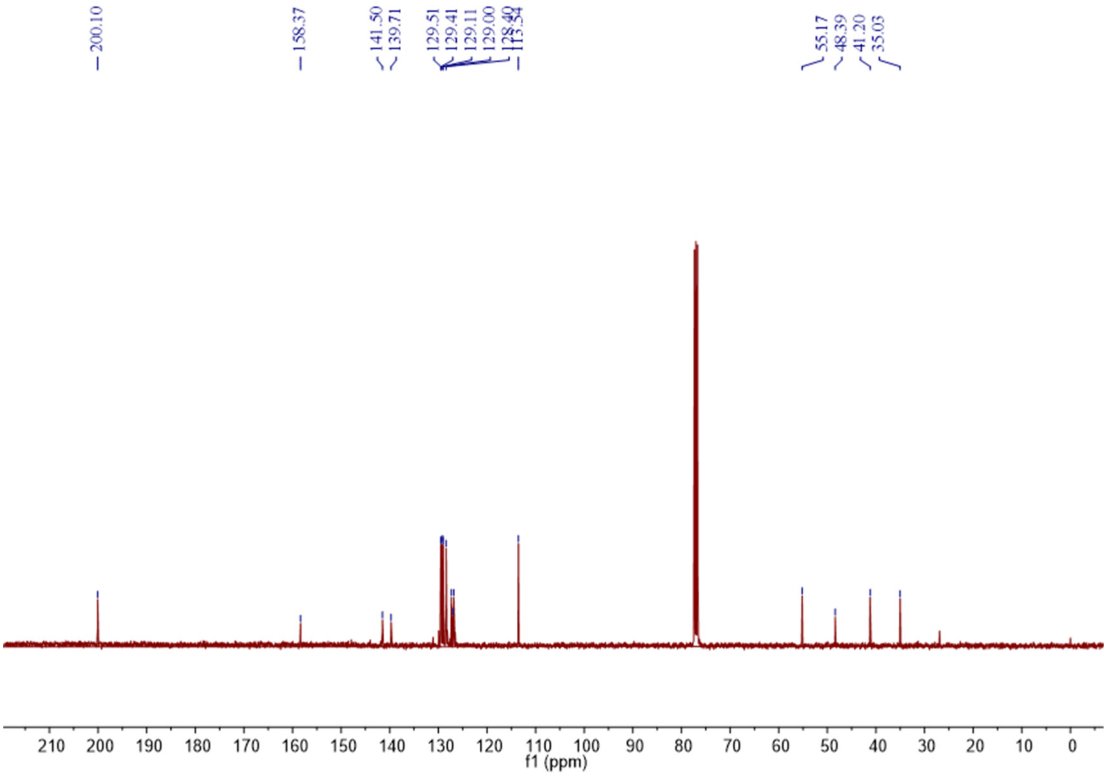
200.10
158.37
141.50
139.71
129.51
129.41
129.11
129.00
55.17
48.39
41.20
35.03
f1 (ppm)

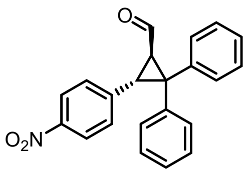

O
O_2N

5d

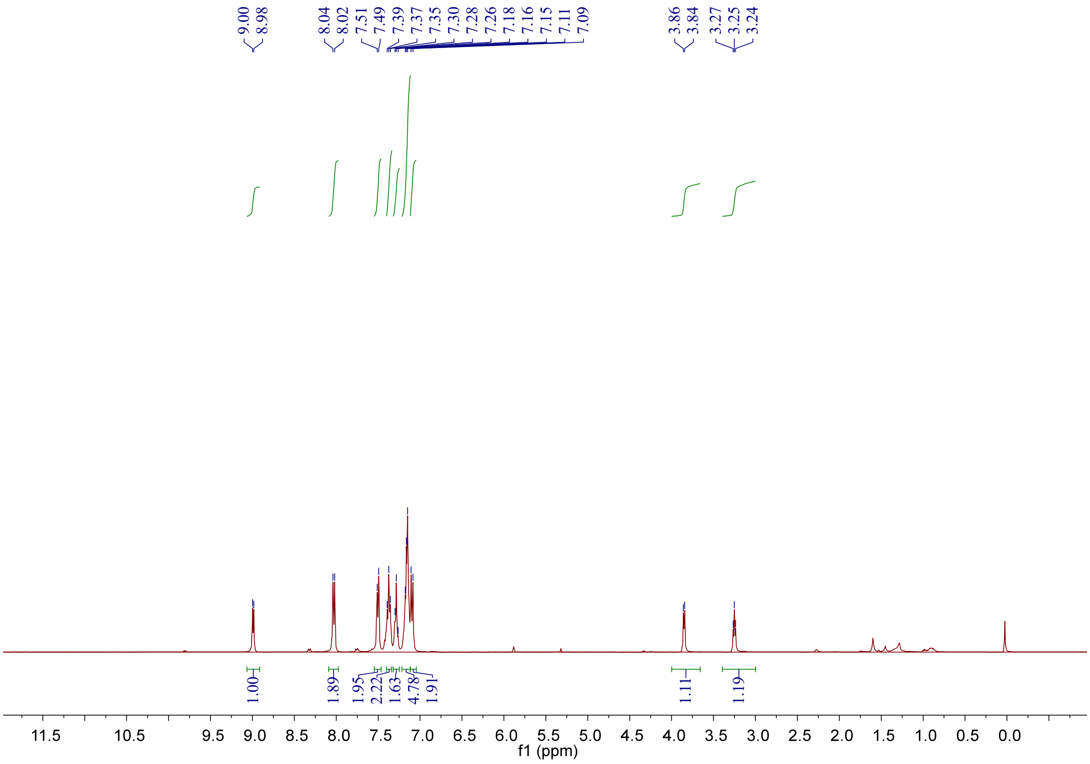

9.00
8.98
8.04
8.02
7.51
7.49
7.39
7.37
7.35
7.30
7.28
7.26
7.18
7.16
7.15
7.11
7.09
3.86
3.84
3.27
3.25
3.24
1.00
1.89
1.95
2.22
1.63
4.78
1.91
1.11
1.19
11.5 10.5 9.5 9.0 8.5 8.0 7.5 7.0 6.5 6.0 5.5 5.0 4.5 4.0 3.5 3.0 2.5 2.0 1.5 1.0 0.5 0.0
f1 (ppm)

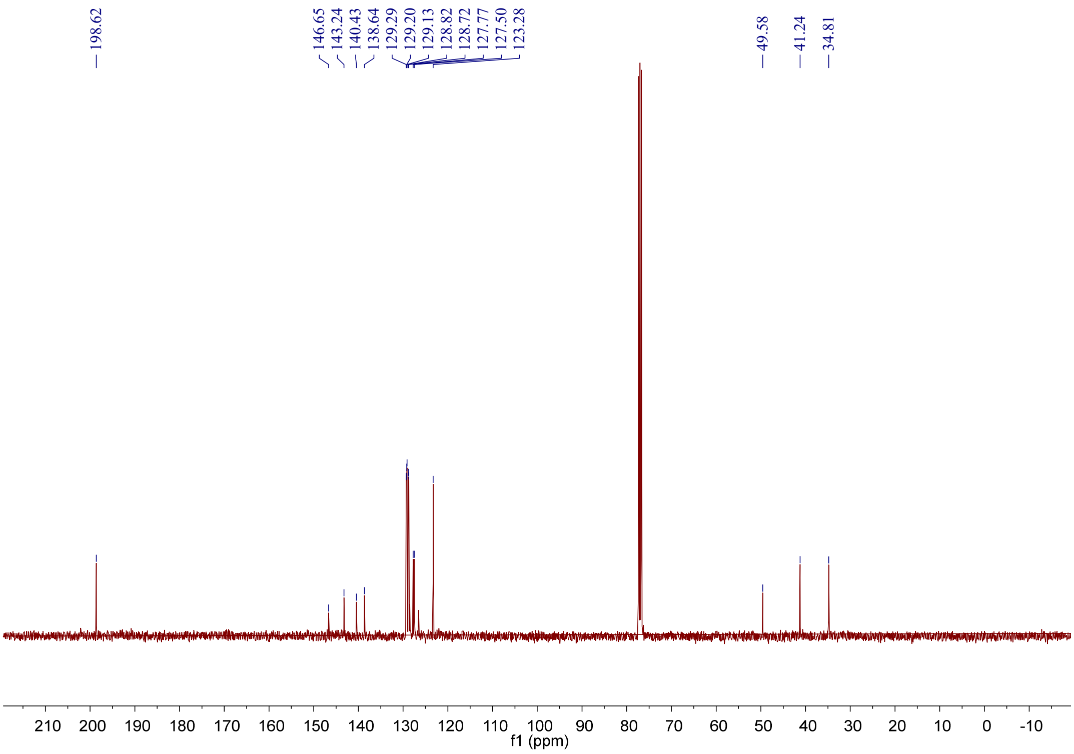

198.62
146.65
143.24
140.43
138.64
129.29
129.20
129.13
128.82
128.72
127.77
127.50
123.28
49.58
41.24
34.81
210 200 190 180 170 160 150 140 130 120 110 100 90 80 70 60 50 40 30 20 10 0 -10
f1 (ppm)

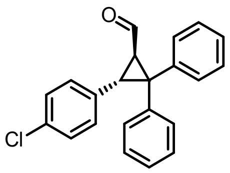
O
Cl

5e

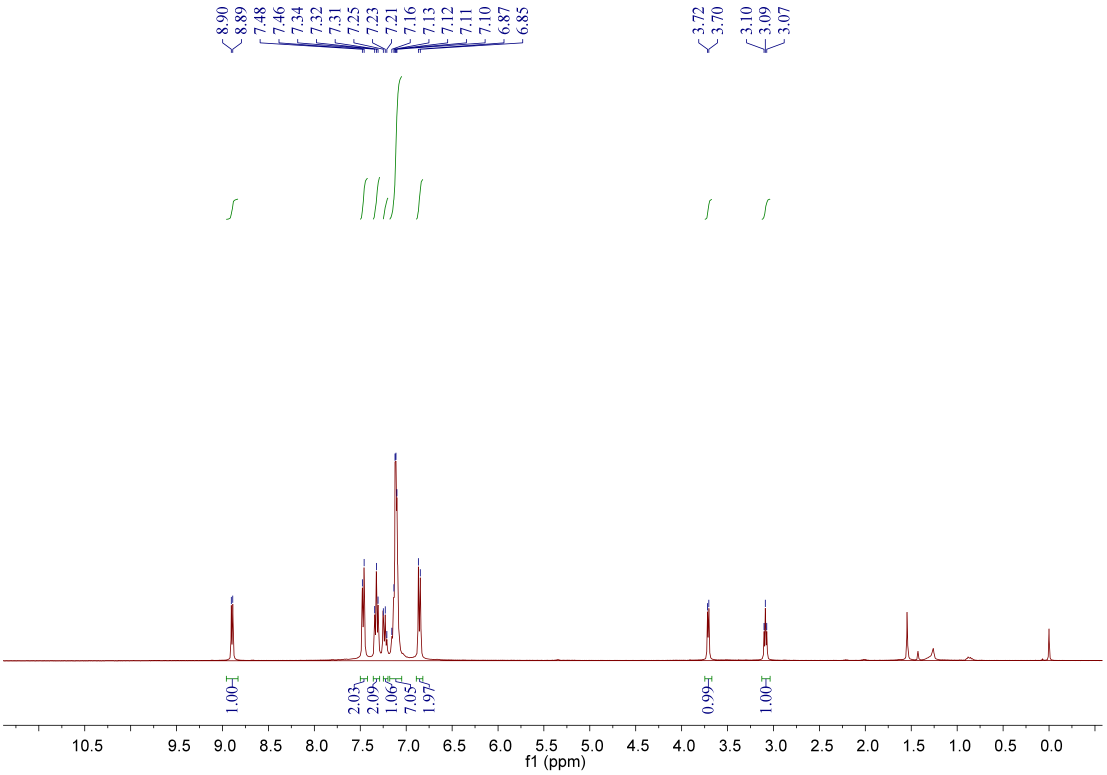
8.90
8.89
7.48
7.46
7.34
7.32
7.31
7.25
7.23
7.21
7.16
7.13
7.12
7.11
7.10
6.87
6.85
3.72
3.70
3.10
3.09
3.07
1.00
2.03
2.09
1.06
7.05
1.97
0.99
1.00
10.5 9.5 9.0 8.5 8.0 7.5 7.0 6.5 6.0 5.5 5.0 4.5 4.0 3.5 3.0 2.5 2.0 1.5 1.0 0.5 0.0
f1 (ppm)

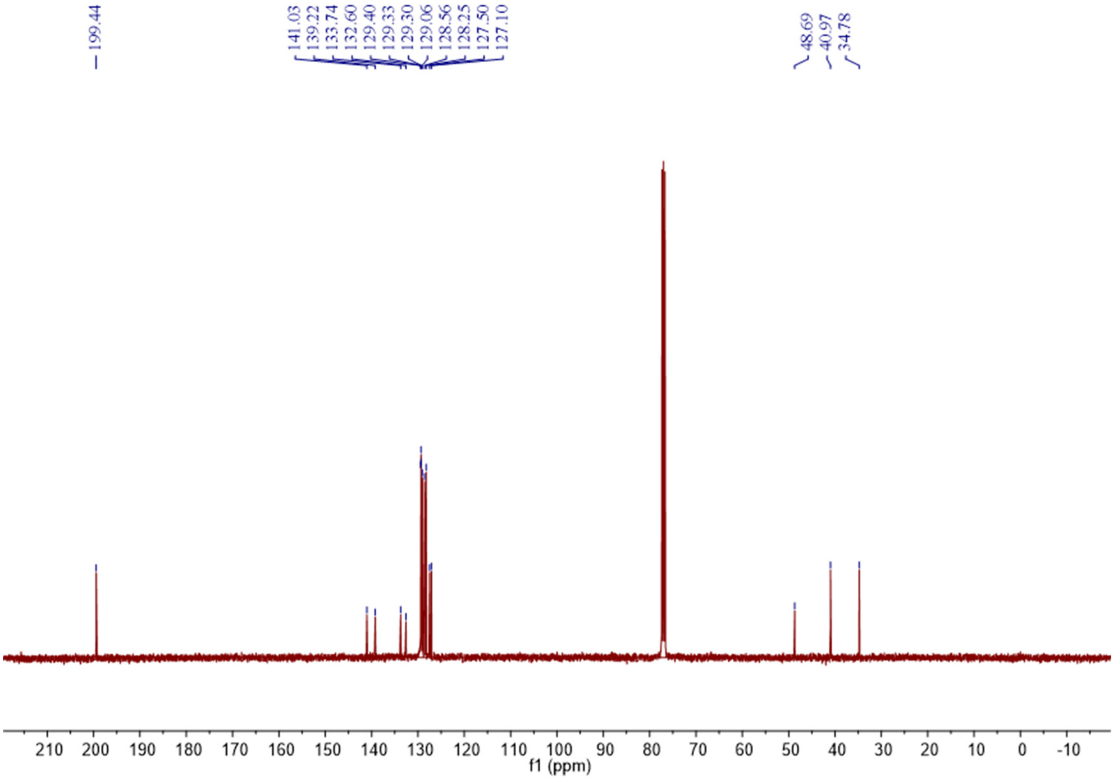
199.44
141.03
139.22
133.74
132.60
129.40
129.33
129.30
129.06
128.56
128.25
127.50
127.10
48.69
40.97
34.78
210 200 190 180 170 160 150 140 130 120 110 100 90 80 70 60 50 40 30 20 10 0 -10
f1 (ppm)

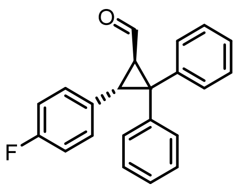

5f

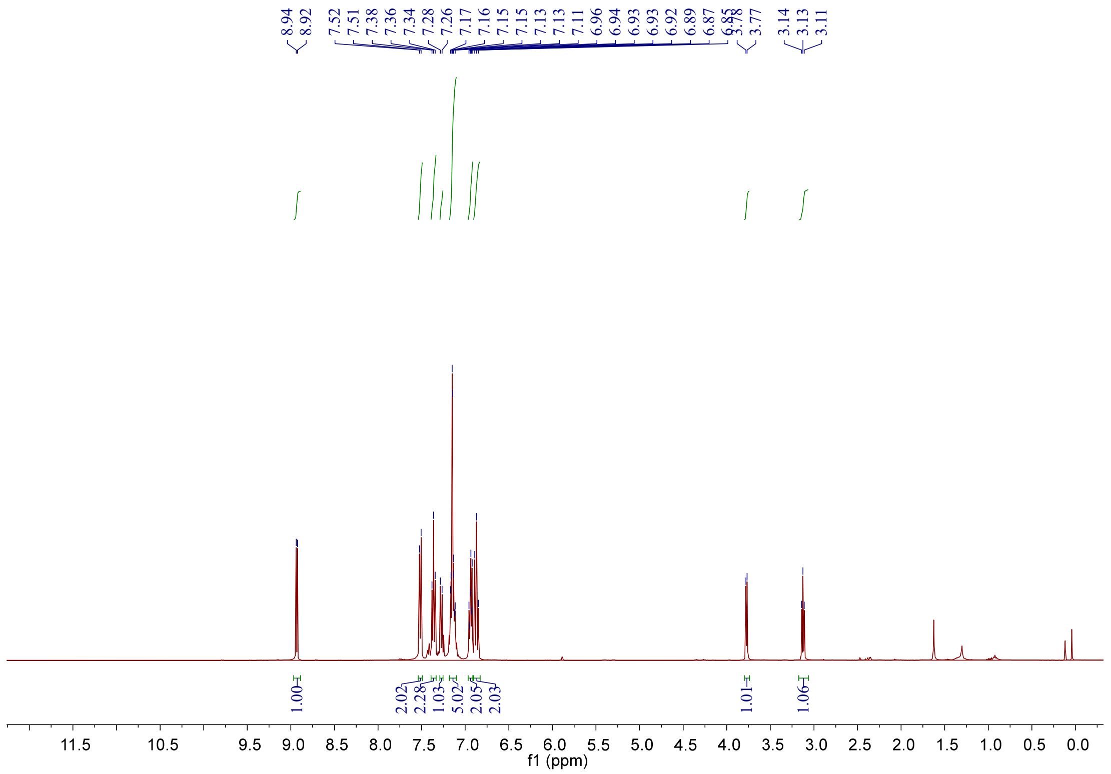

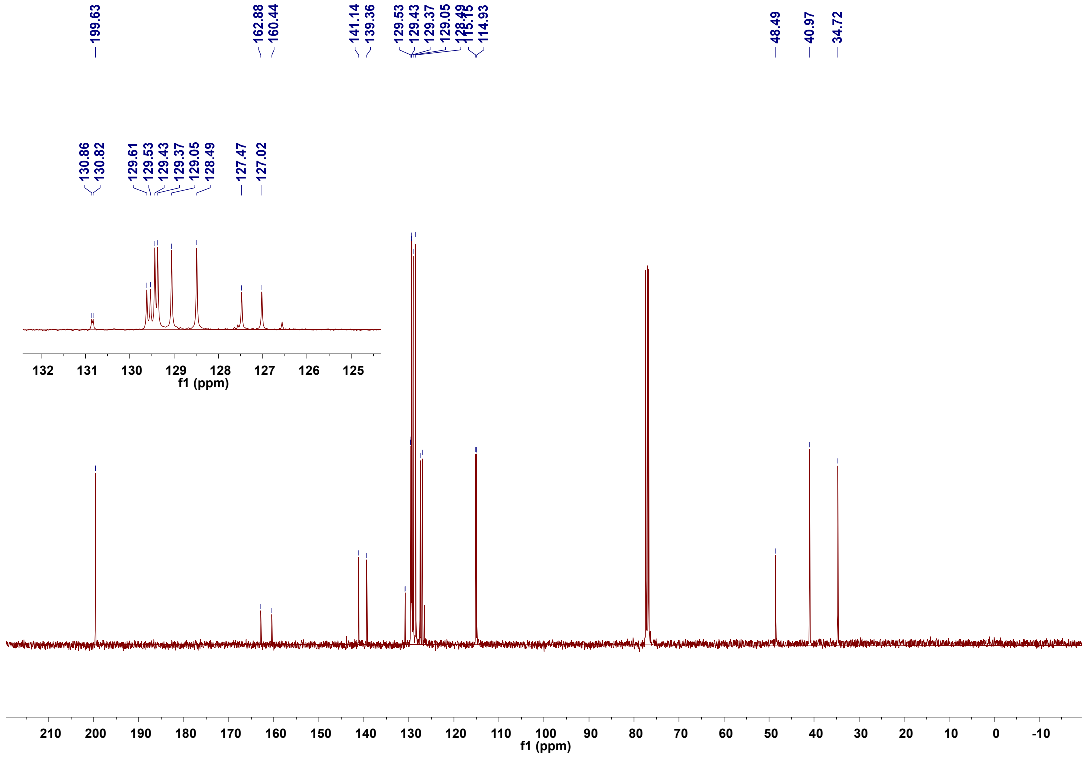

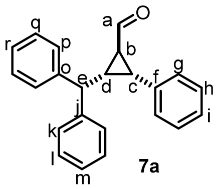

7a

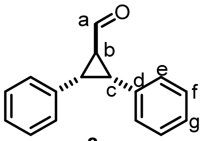

8

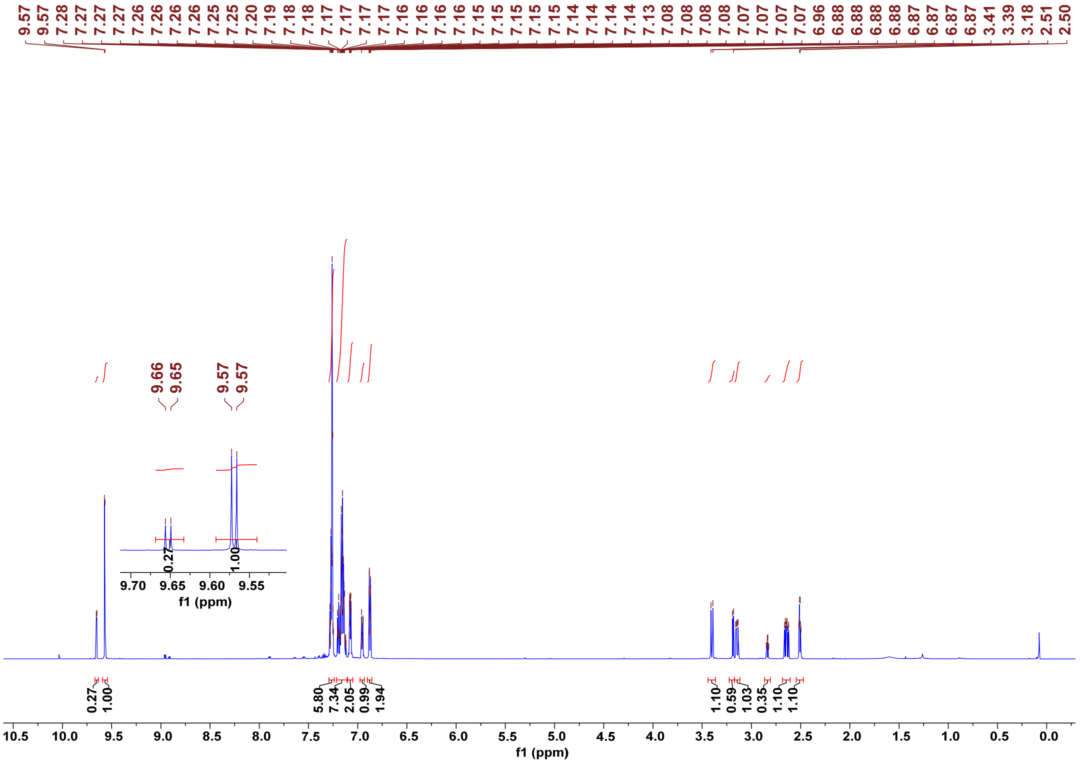

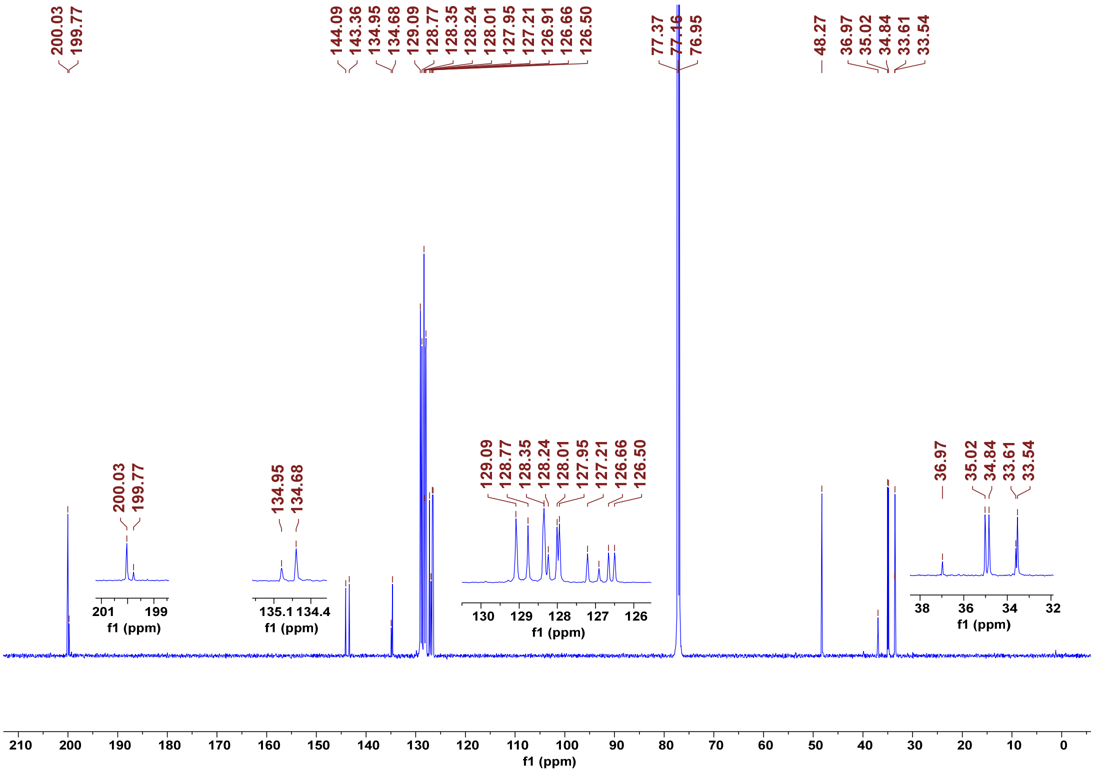

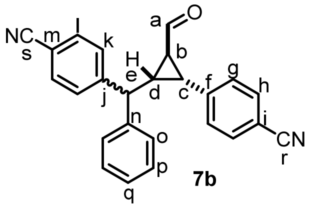

NC
CN
O
H
a
b
c
d
e
f
g
h
i
j
k
l
m
n
o
p
q
r
s

7b

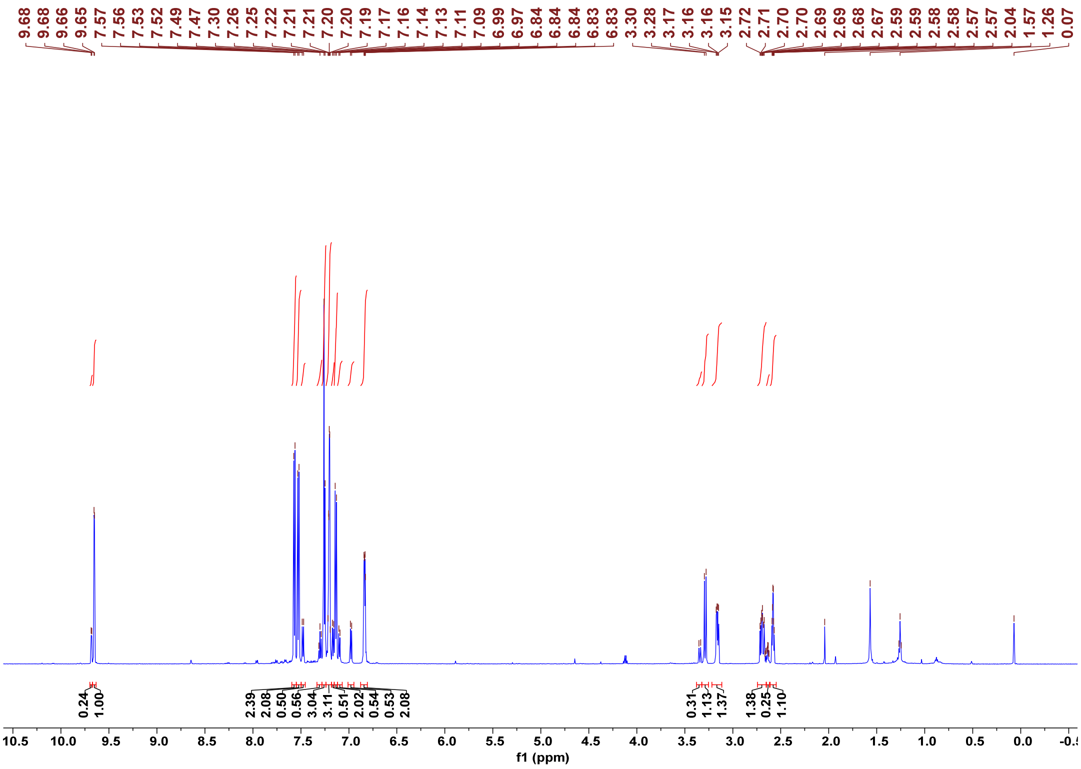

9.68 9.68 9.66 9.65 7.57 7.56 7.53 7.52 7.49 7.47 7.30 7.26 7.25 7.22 7.21 7.21 7.20 7.20 7.19 7.17 7.16 7.14 7.13 7.11 7.09 6.99 6.97 6.84 6.84 6.84 6.83 6.83 3.30 3.28 3.17 3.16 3.16 3.15 2.72 2.71 2.70 2.70 2.69 2.68 2.67 2.59 2.59 2.58 2.58 2.57 2.57 2.04 1.57 1.26 0.07
0.24
1.00
2.39
2.08
0.50
0.56
3.04
3.11
0.51
2.02
0.54
0.53
2.08
0.31
1.13
1.37
1.38
0.25
1.10
10.5 10.0 9.5 9.0 8.5 8.0 7.5 7.0 6.5 6.0 5.5 5.0 4.5 4.0 3.5 3.0 2.5 2.0 1.5 1.0 0.5 0.0
f1 (ppm)

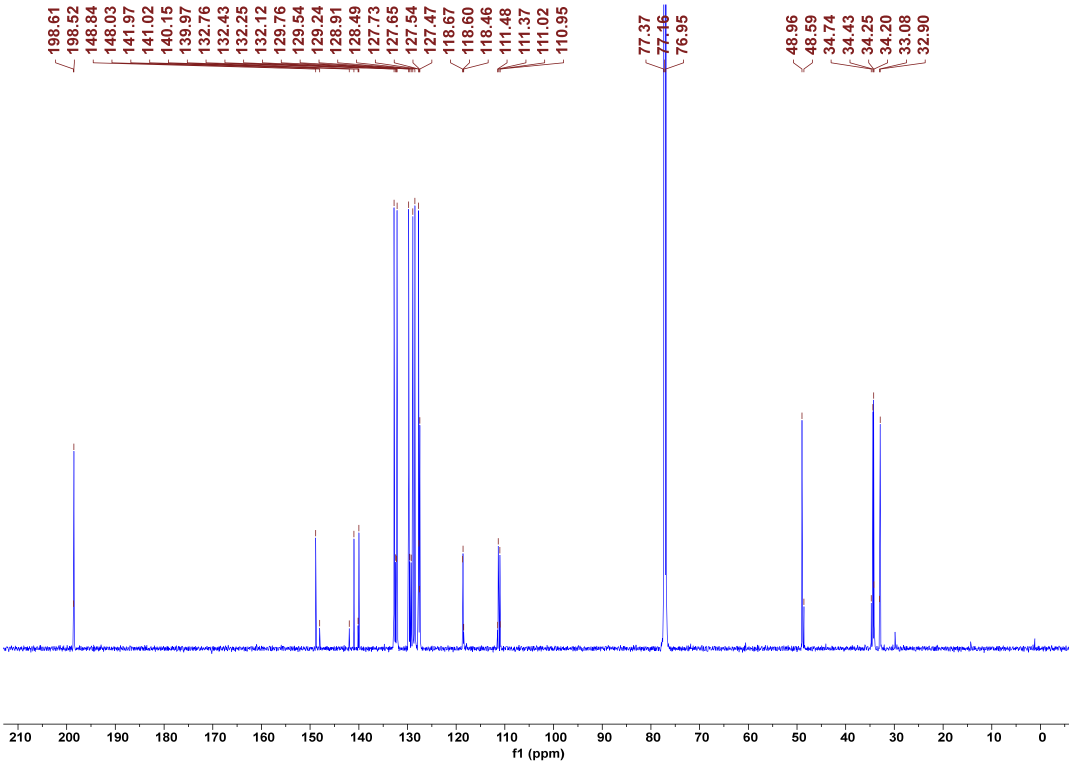

198.61 198.52 148.84 148.03 141.97 141.02 140.15 139.97 132.76 132.43 132.25 132.12 129.76 129.54 129.24 128.91 128.49 127.73 127.65 127.54 127.47 118.67 118.60 118.46 111.48 111.37 111.02 110.95
77.37 77.16 76.95
48.96 48.59 34.74 34.43 34.25 34.20 33.08 32.90
210 200 190 180 170 160 150 140 130 120 110 100 90 80 70 60 50 40 30 20 10 0
f1 (ppm)

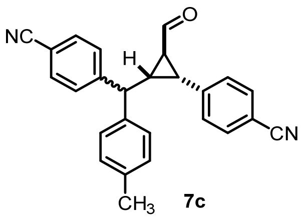
NC
H
O
CN
CH3

7c

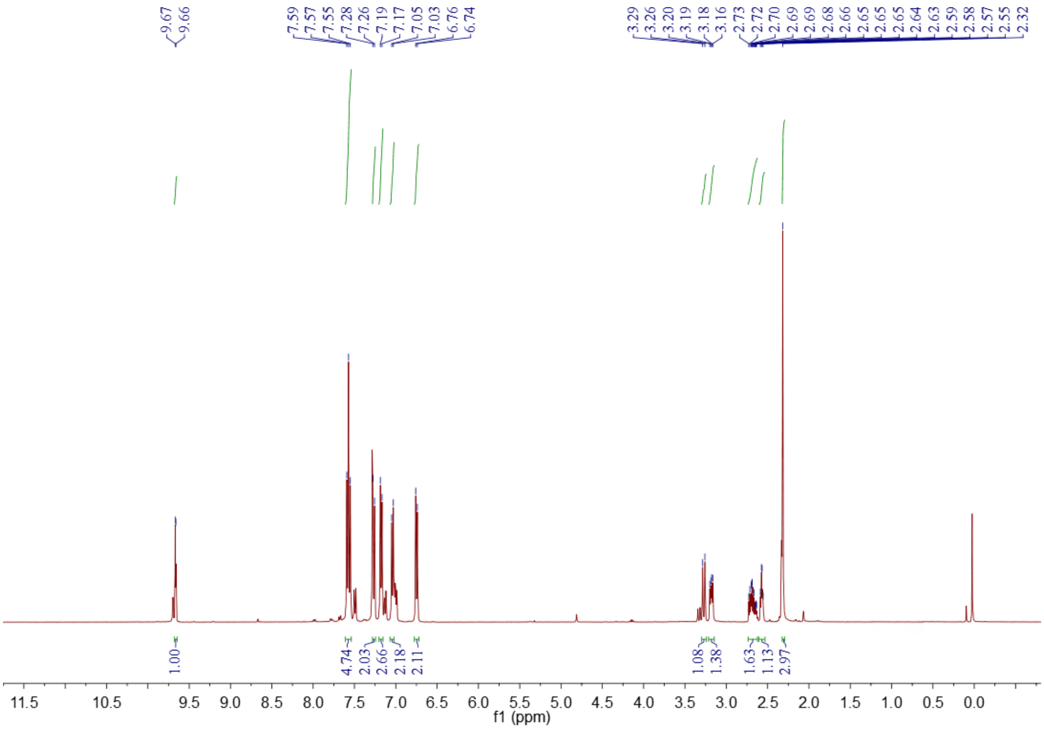
9.67
9.66
7.59
7.57
7.55
7.28
7.26
7.19
7.17
7.05
7.03
6.76
6.74
3.29
3.26
3.20
3.19
3.18
3.16
2.73
2.72
2.70
2.69
2.69
2.68
2.66
2.65
2.65
2.65
2.64
2.63
2.59
2.58
2.57
2.55
2.32
1.00
4.74
2.03
2.66
2.18
2.11
1.08
1.38
1.63
1.13
2.97
11.5 10.5 9.5 9.0 8.5 8.0 7.5 7.0 6.5 6.0 5.5 5.0 4.5 4.0 3.5 3.0 2.5 2.0 1.5 1.0 0.5 0.0
f1 (ppm)

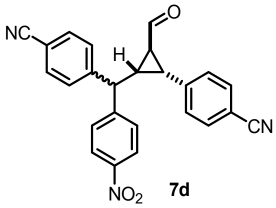

7d

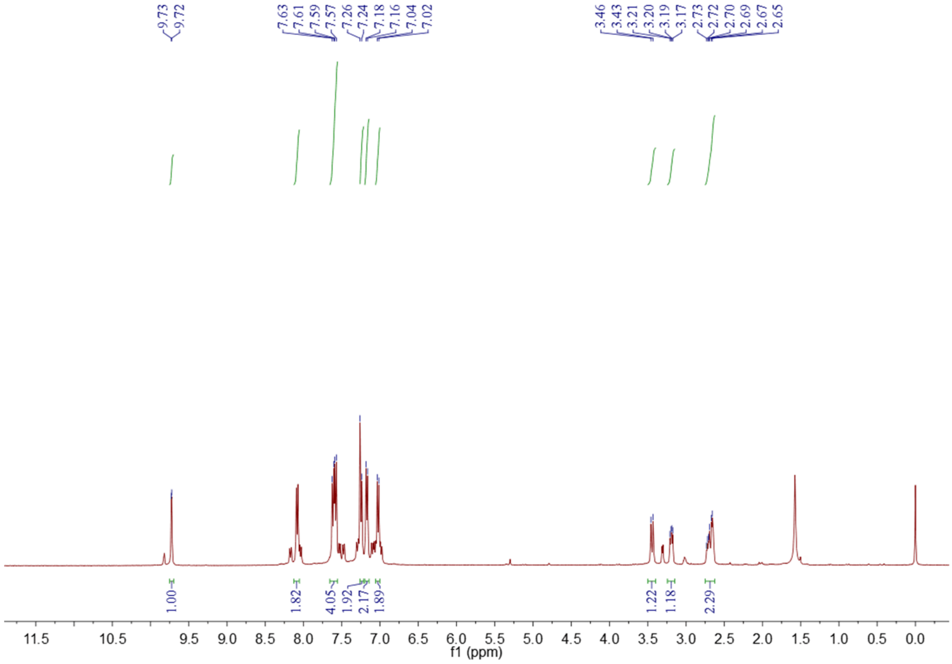

9.73
9.72
7.63
7.61
7.59
7.57
7.26
7.24
7.18
7.16
7.04
7.02
3.46
3.43
3.21
3.20
3.19
3.17
2.73
2.72
2.70
2.69
2.67
2.65
1.00
1.82
4.05
1.92
2.17
1.89
1.22
1.18
2.29
11.5
10.5
9.5
9.0
8.5
8.0
7.5
7.0
6.5
6.0
5.5
5.0
4.5
4.0
3.5
3.0
2.5
2.0
1.5
1.0
0.5
0.0
f1 (ppm)

12. Crystallographic Data

The crystal structure of **5c** could be obtained by the volatilization of solution of **5c** in hexane at ambient temperature. Crystallographic data for **5c** including cif file have been deposited with the Cambridge Crystallographic Data Centre with the numbers of 1963170. Copies of these data can be requested from, free of charge, the CCDC website at https://www.ccdc.cam.ac.uk/structures/.

Crystallographic data for **5c**:

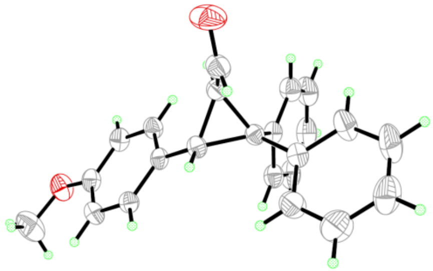

net formula	$C_{23}H_{20}O_2$
M_r/g mol^{-1}	328.39
crystal size/mm	0.2 × 0.05 × 0.02
T/K	293(2)
radiation	CuKα
diffractometer	'SuperNova, Dual, Cu at home/near, AtlasS2'
crystal system	monoclinic
space group	'I 2'
a/Å	13.7279(7)
b/Å	6.5640(4)
c/Å	20.4444(11)
α/°	90
β/°	93.440(5)
γ/°	90
V/Å^3	1838.92(18)
Z	4
calc. density/g cm^{-3}	1.186
μ/mm^{-1}	0.585
absorption correction	Multi-Scan
transmission factor range	N/A
refls. measured	4025
R_{int}	0.0237
mean σ(I)/I	0.0359
θ range	3.776–76.677
observed refls.	2954
x, y (weighting scheme)	0.048, 0.000
hydrogen refinement	C-H: constr

Flack parameter	0.0 (3)
refls in refinement	2954
parameters	228
restraints	1
$R(F_{obs})$	0.0374
$R_w(F^2)$	0.1030
S	1.033
shift/error$_{max}$	0.000
max electron density/e Å^{-3}	0.099
min electron density/e Å^{-3}	−0.113

Formula: $C_{23}H_{20}O_2$.

The crystal structure of **9** could be obtained by the diffusion of THF to the EtOH solution of **9** at ambient temperature. Crystallographic data for **9** including cif file have been deposited with the Cambridge Crystallographic Data Centre with the numbers of 1964337. Copies of these data can be requested from, free of charge, the CCDC website at https://www.ccdc.cam.ac.uk/structures/.

Crystallographic data for **9**:

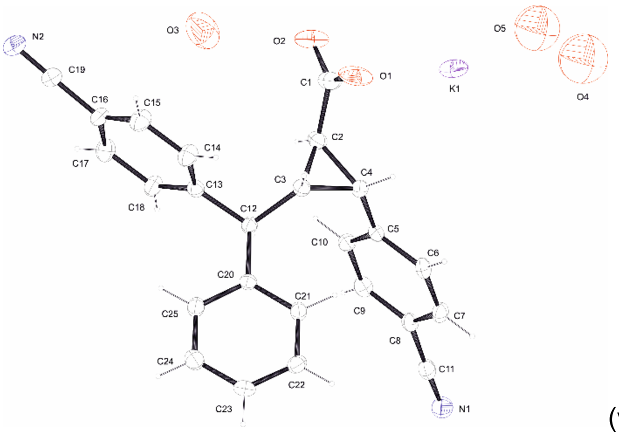

(vv019)

net formula	$C_{25}H_{20}KN_2O_{3.50}$
M_r/g mol^{-1}	443.53
crystal size/mm	0.100 × 0.060 × 0.030
T/K	100.(2)
radiation	MoKα
diffractometer	'Bruker D8 Venture TXS'
crystal system	monoclinic
space group	'P 1 21 1'
a/Å	10.0045(3)
b/Å	6.0051(2)
c/Å	19.7363(5)

α/°	90
β/°	97.1210(10)
γ/°	90
V/Å^3	1176.57(6)
Z	2
calc. density/g cm^{-3}	1.252
μ/mm^{-1}	0.255
absorption correction	Multi-Scan
transmission factor range	0.6704–0.7454
refls. measured	9838
R_{int}	0.0258
mean $\sigma(I)/I$	0.0460
θ range	3.516–26.371
observed refls.	4242
x, y (weighting scheme)	0.1032, 0.4254
hydrogen refinement	C-H: constr, O-H: not considered
Flack parameter	0.020(19)
refls in refinement	4796
parameters	288
restraints	1
$R(F_{obs})$	0.0539
$R_w(F^2)$	0.1615
S	1.057
shift/error$_{max}$	0.001
max electron density/e Å^{-3}	0.786
min electron density/e Å^{-3}	−0.373

Formula: $KC_{25}H_{17}N_2O_2 \cdot 1.5\ H_2O$. The water content is estimated since their sof's have been fixed to values obtained by free refinement and consideration of acceptable thermal parameters.

13. References

[S1] (a) K. A. Ahrendt, C. J. Borths, D. W. C. MacMillan, *J. Am. Chem. Soc.* **2000**, *122*, 4243-4244; (b) F. An, S. Paul, J. Ammer, A. R. Ofial, P. Mayer, S. Lakhdar, H. Mayr, *Asian J. Org. Chem* **2014**, *3*, 550-555; (c) U. Grošelj, A. Beck, W. B. Schweizer, D. Seebach, *Helv. Chim. Acta* **2014**, *97*, 751-796; (d) M. Silvi, C. Verrier, Y. P. Rey, L. Buzzetti, P. Melchiorre, *Nat. Chem.* **2017**, *9*, 868.

[S2] (a) T. Bug, M. Hartnagel, C. Schlierf, H. Mayr, *Chem. Eur. J.* **2003**, *9*, 4068-4076; (b) H. Jangra, Q. Chen, E. Fuks, I. Zenz, P. Mayer, A. R. Ofial, H. Zipse, H. Mayr, *J. Am. Chem. Soc.* **2018**, *140*, 16758-16772.

[S3] B. L. Ryland, S. D. McCann, T. C. Brunold, S. S. Stahl, *J. Am. Chem. Soc.* **2014**, *136*, 12166-12173.

[S4] Harder, E.; Damm, W.; Maple, J.; Wu, C.; Reboul, M.; Xiang, J. Y.; Wang, L.; Lupyan, D.; Dahlgren, M. K.; Knight, J.L.; Kaus, J. W.; Cerutti, D. S.; Krilov, G.; Jorgensen, W. L.; Abel, R.; Friesner, R. A. *J. Chem. Theory Comput.* **2016**, *12*, 281-296.

[S5] Schrödinger Release 2019-4: MacroModel, Schrödinger, LLC, New York, NY, 2019.

[S6] Gaussian 16, Revision A.03, Frisch, M. J.; Trucks, G. W.; Schlegel, H. B.; Scuseria, G. E.; Robb, M. A.; Cheeseman, J. R.; Scalmani, G.; Barone, V.; Petersson, G. A.; Nakatsuji, H.; Li, X.; Caricato, M.; Marenich, A. V.; Bloino, J.; Janesko, B. G.; Gomperts, R.; Mennucci, B.; Hratchian, H. P.; Ortiz, J. V.; Izmaylov, A. F.; Sonnenberg, J. L.; Williams-Young, D.; Ding, F.; Lipparini, F.; Egidi, F.; Goings, J.; Peng, B.; Petrone, A.; Henderson, T.; Ranasinghe, D.; Zakrzewski, V. G.; Gao, J.; Rega, N.; Zheng, G.; Liang, W.; Hada, M.; Ehara, M.; Toyota, K.; Fukuda, R.; Hasegawa, J.; Ishida, M.; Nakajima, T.; Honda, Y.; Kitao, O.; Nakai, H.; Vreven, T.; Throssell, K.; Montgomery, J. A., Jr.; Peralta, J. E.; Ogliaro, F.; Bearpark, M. J.; Heyd, J. J.; Brothers, E. N.; Kudin, K. N.; Staroverov, V. N.; Keith, T. A.; Kobayashi, R.; Normand, J.; Raghavachari, K.; Rendell, A. P.; Burant, J. C.; Iyengar, S. S.; Tomasi, J.; Cossi, M.; Millam, J. M.; Klene, M.; Adamo, C.; Cammi, R.; Ochterski, J. W.; Martin, R. L.; Morokuma, K.; Farkas, O.; Foresman, J. B.; Fox, D. J. Gaussian, Inc., Wallingford CT, 2016.

[S7] a) Becke, A. D. *J. Chem. Phys.* **1993**, *98*, 5648–5652; b) F. Weigend, R. Ahlrichs, *Phys. Chem. Chem. Phys.* **2005**, *7*, 3297-3305.

[S8] A. V. Marenich, C. J. Cramer, D. G. Truhlar, *J. Phys. Chem. B* **2009**, *113*, 6378-6396.

[S9] S. Grimme, S. Ehrlich, L. Goerigk, *J. Comput. Chem.* **2011**, *32*, 1456-1465.
